# Discovery of New Quinazolinone and Benzimidazole Analogs as Tubulin Polymerization Inhibitors with Potent Anticancer Activities

**DOI:** 10.3390/ph19010161

**Published:** 2026-01-15

**Authors:** Boye Jiang, Juan Zhang, Kai Shao, Conghao Gai, Bing Xu, Yan Zou, Yan Song, Qingjie Zhao, Qingguo Meng, Xiaoyun Chai

**Affiliations:** 1 Department of Organic Chemistry, School of Pharmacy, Second Military Medical University, Shanghai 200433, China; by18303453602@163.com (B.J.); shaokai_621@163.com (K.S.); c.gai2@outlook.com (C.G.); xubing_0520@163.com (B.X.); zouyan0622@126.com (Y.Z.); qjzhao_325@126.com (Q.Z.); 2 PLA Naval Medical Center, Second Military Medical University, Shanghai 200052, China; lyzj0706@163.com (J.Z.); songyan455@126.com (Y.S.); 3 School of Pharmacy, Yantai University, Yantai 264005, China

**Keywords:** tubulin, anticancer activity, molecular hybridization

## Abstract

**Background/Objectives:** Cancer persists as a leading concern in the current medical field, and current therapies are limited by toxicity, cost, and resistance. Targeted inhibition of tubulin polymerization is considered as a promising therapeutic strategy for cancer treatment. **Methods**: Thirty-one new tubulin polymerization inhibitors were designed via molecular hybridization techniques, and BLI technology was employed to quantitatively investigate their interactions with tubulin. Antiproliferative activities against MCF-7, MDA-MB-231, A549, and HeLa cell lines was evaluated using the CCK8 assay. Apoptosis induction and cell cycle arrest were analyzed by flow cytometry. The anti-tumor activity of compound **B6** was validated in a mouse melanoma tumor model. **Results**: Compounds exhibited varying degrees of antiproliferative activity against four tumor cell lines. Among them, compound **B6** was the most promising candidate and displayed strong broad-spectrum anticancer activity with an average IC_50_ value of 2 μM. The mechanism studies revealed that compound **B6** inhibited tubulin polymerization in vitro, disrupted cell microtubule networks, and arrested the cell cycle at G2/M phase. Furthermore, **B6** displayed significant in vivo antitumor efficacy in a melanoma tumor model with tumor growth inhibition rates of 70.21% (50 mg/kg). **Conclusions**: This work shows that **B6** is a promising lead compound deserving further investigation as a potential anticancer agent.

## 1. Introduction

Cancer represents a significant health burden and is the second leading cause of death worldwide after cardiovascular disease [1,2,3,4]. In 2020, it accounted for approximately 10 million deaths and 19.3 million new diagnoses. By 2040, global new cancer cases are projected to reach 28.4 million, an increase of 47% compared to 2020 [5]. Chemotherapy utilizing anticancer agents derived from natural and synthetic sources remains the primary treatment modality for cancer.

Microtubules, core components of the cytoskeleton, are ubiquitously distributed throughout the cytoplasmic matrix and play critical roles in maintaining cellular architecture and regulating cellular functions. Beyond providing mechanical support, they participate in dynamic remodeling such as structural reorganization mediated by dynamic instability and contribute to cellular morphological and structural integrity. As highly dynamic protein polymers, microtubules facilitate precise chromosome segregation during mitosis while coordinating spatiotemporal organelle transport and signal transduction pathways [6,7,8]. During mitosis, microtubules undergo extensive structural rearrangement to assemble a bipolar spindle. This process relies on precise regulation of microtubule dynamic instability to ensure accurate kinetochore-microtubule attachments and mediate equal sister chromatid separation. Notably, aberrant chromosome-spindle attachments or segregation errors can trigger sustained spindle assembly checkpoint activation, leading to G2/M phase arrest and prolonged mitotic arrest. In malignant cells, extended mitotic arrest may further activate intrinsic apoptosis pathways—the core antitumor mechanism by microtubule-targeting agents (MTAs) [9].

**Figure 1:**
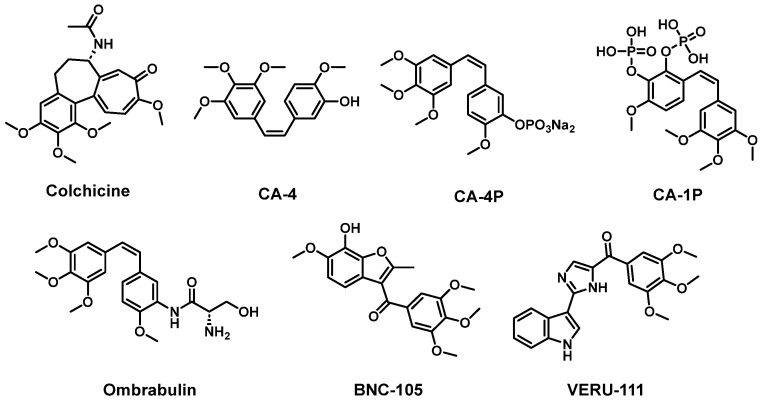
Representative tubulin inhibitors targeting the colchicine binding site.

Due to their essential mitotic functions, the microtubule system represents a major target for novel anticancer drugs. Currently, the most extensively studied MTAs can be broadly categorized into two classes: microtubule stabilizers (e.g., paclitaxel) and destabilizers (e.g., vinca alkaloids and colchicine) [10,11,12]. Researchers have investigated numerous tubulin inhibitors with various scaffolds (Figure 1). Combretastatin A4 (CA–4), a natural *cis*-stilbene derivative isolated from *Combretum caffrum*, exhibits dual antimitotic and vascular disrupting activities [13]. To address its poor aqueous solubility, the phosphate prodrug CA–4P was developed and has completed Phase II trials for anaplastic thyroid cancer [14]. Several structurally optimized CA-4 analogs are advancing clinically, including ombrabulin (phase II for NSCLC) [15], Oxi4503 (a safer CA–1 prodrug in phase II for relapsed/refractory AML) [16], and BNC-105 (Phase II for metastatic CRC) [17]. Notably, the oral candidate VERU–111 (ABI-231) shows promise for advanced prostate cancer, demonstrating potent antiproliferative effects in taxane-resistant models with favorable pharmacokinetics (21–50% oral bioavailability) [18]. Consequently, there is growing interest in developing innovative tubulin inhibitors for cancer treatment.

Quinazolinones, an important class of nitrogen-containing heterocyclic pharmacophores, hold extensive application value in medicinal chemistry. Their structural versatility and multifunctional modification sites render them privileged scaffolds for developing diverse therapeutic agents. Clinically approved quinazolinone derivatives include anticancer kinase inhibitors such as gefitinib and erlotinib [19]. Recently, microtubule-targeting quinazolinone-based compounds have been widely reported, exhibiting mechanisms distinct from traditional kinase inhibitors [20,21,22,23].

**Figure 2:**
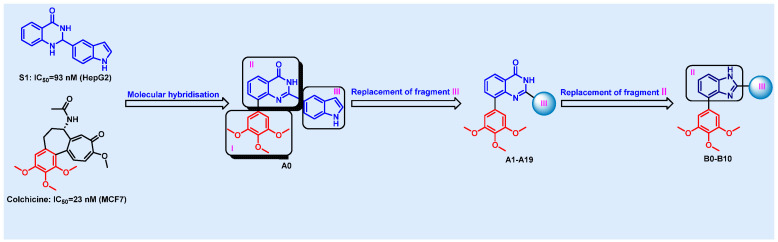
Design of target compounds by the ring-fusion and molecular hybridization strategy.

2-Substituted-2,3-dihydroquinoxaline-4(1*H*)-one derivatives (**S1**) inhibit microtubulin polymerization in vitro, disrupt the cellular microtubule network, arrest the cell cycle at the G2/M phase, and induce apoptosis by upregulating the expression of cleaved PARP-1 and caspase-3. Furthermore, in a nude mouse model, compound **S1** significantly inhibited the growth of HepG2 xenograft tumors via oral gavage administration without exhibiting marked toxicity. Compound **S1** demonstrates potential as a microtubulin polymerization inhibitor and a novel antitumor therapeutic agent. To explore more efficient anti-tumor drugs that target the colchicine-binding site of tubulin, we designed compounds based on chemical design strategies. In this study, compound **A0** was designed through molecular hybridization of colchicine with the quinazolinone derivative **S1** (acting at the colchicine-binding site of tubulin) [24]. As illustrated in Figure 2, compound **A0** can be divided into three distinct moieties: the 3,4,5-trimethoxyphenyl (TMP) group (fragment I), the heterocyclic core (fragment II), and the indole ring (fragment III). Given the high affinity exhibited by TMP at the colchicine-binding site, this pharmacophore has emerged as a favorable molecular skeleton for developing antimitotic agents and tubulin polymerization inhibitors [25,26].

Considering the poor interaction between compound **A0** and microtubule proteins and its anti-tumor activity in vitro, we first introduced aryl/heteroaromatic/cycloalkyl substituted analogs with different substitution modes through drug design theory to design compounds of the **A** series. Among them, compound **A5** has the strongest binding ability to microtubule proteins and exhibits certain anti-tumor activity against MCF-7 cells. Given the reports that fused heterocycles (such as thienopyridine) exhibit potent anti-tumor activity through multi-point microtubule protein interactions [27,28], we employed structure-based design to modify the quinazolinone core of **A5** while preserving the critical TMP motif and adjacent trifluoromethyl group. Through scaffold hopping, compound **B0** featuring a benzimidazole core was designed. We observed that among the **A**-series compounds, those bearing aromatic substituents (**A1**, **A5**, and **A12**) exhibited favorable binding affinity to microtubulin and anti-proliferative activity against MCF-7 cells. Consequently, aromatic substituents were similarly incorporated into the compounds of **B** series to identify optimal antitumor agents. This series aims to provide colchicine binding site inhibitors with enhanced selectivity and potency, laying the foundation for next-generation anticancer therapeutics.

## 2. Results and Discussion

### 2.1. Chemistry

**Scheme 1:**
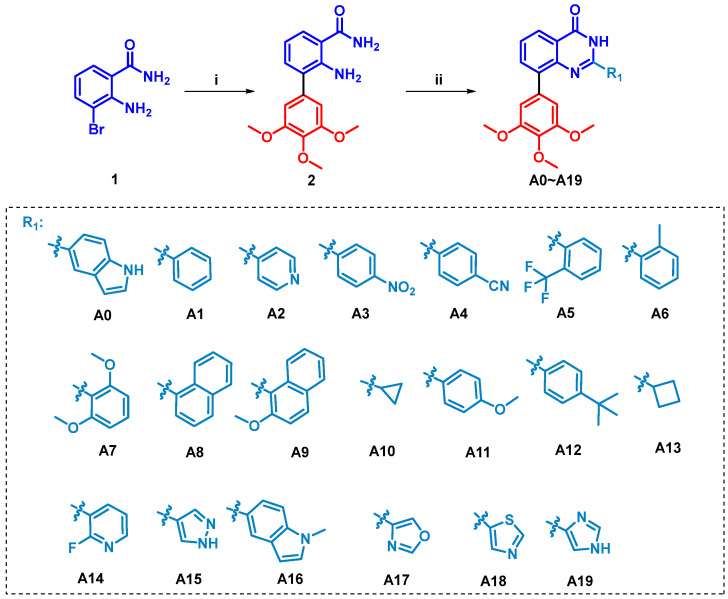
Reagents and conditions: (i) 3,4,5-trimethoxyphenylboronic acid, Pd(dppf)Cl_2_·CH_2_Cl_2_, K_2_CO_3_, THF/H_2_O, 75 °C; (ii) substituted aldehyde, DMSO, 120 °C.

The synthesis of **A0**–**A19** is described in Scheme 1. 2-Amino-3-bromobenzamide (**1**) and 3,4,5-trimethoxyphenylboronic acid were used as starting materials, and the intermediate **2** was obtained through Suzuki coupling [29]. Compound **2** was then cyclized with various substituted aromatic aldehydes to yield compounds **A0**–**A19** [30].

**Scheme 2:**
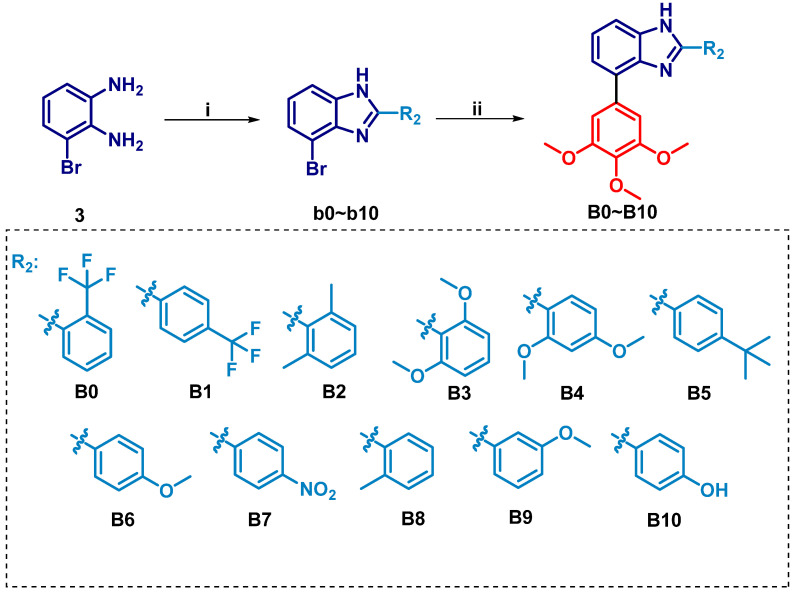
Reagents and conditions: (i) substituted aromatic aldehyde, TsOH, 1,4-dioxane, 95 °C; (ii) 3,4,5-trimethoxyphenylboronic acid, K_2_CO_3_, PdCl_2_(PPh_3_)_2_, DMF, 80 °C.

The synthesis of series **B** compounds (**B0**–**B10**) is illustrated in Scheme 2. 3-Bromo-1,2-benzenediamine reacted with different substituted aromatic aldehydes in 1,4-dioxane to obtain the intermediate **b0–b10**. Intermediates **b0–b10** and 3,4,5-trimethoxyphenylboronic acid underwent Suzuki couplings to obtain the target compounds **B0**–**B10** to explore the structural diversity.

### 2.2. Biology

#### 2.2.1. Binding Affinity to Tubulin Protein Analysis

To detect direct interactions between synthetic compounds and tubulin, bio-layer interferometry (BLI) experiments [31] were conducted using purified porcine brain tubulin immobilized on super sensing avidin (SSA) biosensors, with the affinity between the two quantified (Table 1). The strength of affinity was expressed via the binding dissociation equilibrium constant K_D_. A smaller K_D_ value indicates greater affinity between the compound and the tubulin protein.

Results indicate that within the concentration range of 10^−4^ to 10^−7^ mol·L^−1^, all target compounds except for **A10**, **A12**, **A16**, and **B2** exhibited clear concentration-dependent binding to tubulin. Moreover, most compounds exhibited strong binding affinity to tubulin protein, with compounds **A5**, **A15**, **B3**, **B4**, **B5**, and **B6** demonstrating superior binding potency to tubulin compared to colchicine.

#### 2.2.2. In Vitro Antiproliferative Activity

**Figure 3:**
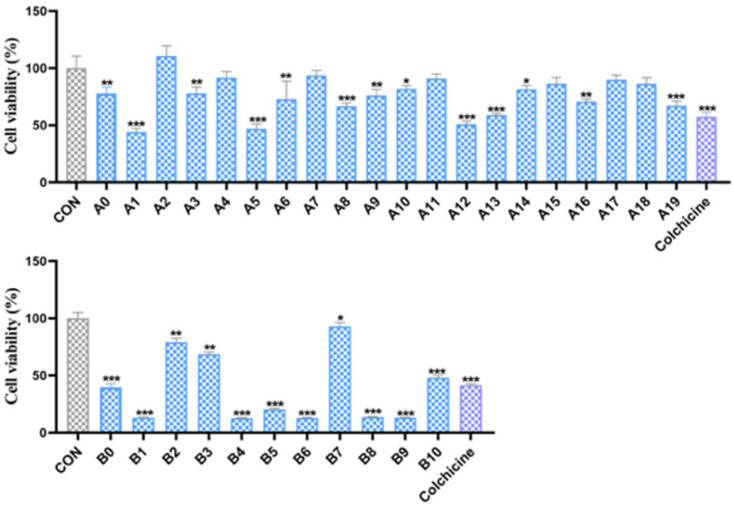
Inhibition of cell viability and/or proliferation in MCF-7 cells following treatment (48 h) with the tested compounds (50 μM) (*n* = 3, * *p* < 0.05, ** *p* < 0.01, and *** *p* < 0.001, when compared with the control group).

The in vitro cytotoxicity of the newly synthesized compounds **A0**–**A19** and **B0**–**B10** towards MCF-7 cells was evaluated using the CCK-8 assay, with colchicine (50 nM) serving as the positive control. As shown in Figure 3, compounds of the **B** series (50 μM) exhibited moderate to potent antiproliferative activity against MCF-7 cells, significantly outperforming those from compounds of the **A** series. Notably, compounds **B1**, **B4**–**B6**, **B8**, and **B9** exhibited a high inhibition rate against MCF-7 cells. Furthermore, we observed that introducing a methoxy group at the p-position of the benzene ring in fragment III (e.g., **B6**, inhibition rate: 88.13%) or a tert-butyl group (e.g., **B5**, inhibition rate: 79.64%) significantly enhanced activity, whereas nitro (e.g., **B7**, inhibition rate: 7.15%) and hydroxyl groups (e.g., **B10**, inhibition rate: 51.99%) were detrimental to activity.

Further analysis showed that among derivatives with the same substituents on fragment III, compounds of the **B** series characterized by 1*H* benzimidazole in fragment II exhibited significantly superior anti-tumor activity compared to the **A** series. For example, compounds **A5** and **B0** with adjacent trifluoromethyl groups showed inhibition rates of 53.12% and 60.28% on MCF-7 cells, respectively. Similarly, compounds **A11** and **B6**, both bearing methoxy groups in the p-position, exhibited comparable trends, with inhibition rates against MCF-7 cells of 9.14% and 88.13%, respectively. This indicates that fragments I and II are crucial for activity. Due to the differences in fragment II, the activity of B-series compounds was significantly better than that of **A**-series compounds. This further indicates that in the **B** series, the spatial conformation of the benzimidazole core and TMP fragment was closer to the colchicine binding site, thereby synergistically enhancing affinity.

Based on the preliminary screening results, we selected compounds **B1**, **B4**–**B6**, and **B8**–**B10** with the best antitumor activity in the **B** series for further evaluation of their in vitro antitumor activities. We determined the IC_50_ values of compounds **B1**, **B4**–**B6**, and **B8**–**B10** against MCF-7, MDA-MB-231, A549, and Hela cells through the CCK-8 method using colchicine as positive control drugs.

As shown in Table 2, except for compound **B10**, compounds **B1** and **B4**–**B9** exhibited high antiproliferative activity against four tumor cell lines. Among them, compound **B6** showed the best performance among the six compounds with IC_50_ values of 1.4 μM, 2.5 μM, 1.8 μM, 1.8 μM, and 0.4 μM against MCF-7, MDA-MB-231, A549, and Hela cells, respectively. While compounds **B1** and **B4**–**B9** showed similar antiproliferative activities against MCF-7, A549, and Hela cells (IC_50_ < 10 μM), the antiproliferative effects on MDA-MB-231 cells (IC_50_ > 10 μM) were slightly weaker than those on the other four tumor cell lines.

In summary, fragment I (trimethoxyphenyl) and fragment II (1*H*-benzimidazole) are the key pharmacophores for maintaining the antitumor activity of the derivatives, and their synergistic effect is the basis of this activity.

#### 2.2.3. In Vitro Inhibition of Tubulin Polymerization

**Figure 4:**
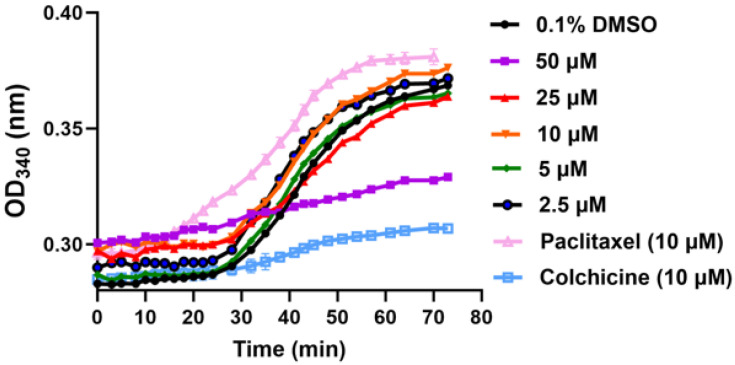
Inhibition of tubulin polymerization by compound **B6**. Data are expressed as the mean ± standard deviation from three independent experiment.

In light of the remarkable in vitro antiproliferative activities exhibited by the target compounds, we further investigated the ability of compound **B6** to inhibit tubulin polymerization; in this assay, colchicine (10 μM) was used as the positive control while paclitaxel (10 μM) acted as the negative control. As shown in Figure 4, the results indicate that at a concentration of 50 μM, the compound **B6** can significantly inhibit in vitro tubulin polymerization.

#### 2.2.4. Immunofluorescence Analysis

**Figure 5:**
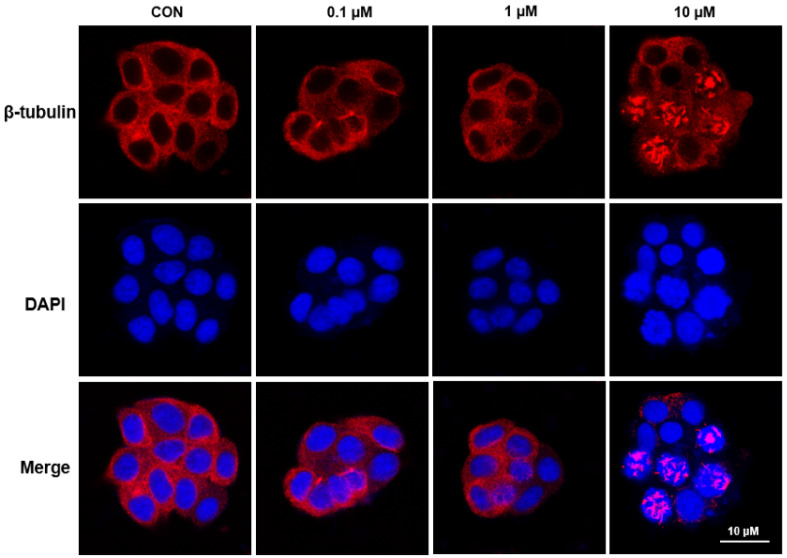
Impacts of compound **B6** on cellular microtubules. Microtubule structures were labeled with an anti-β-tubulin primary antibody (fluorescent red), while cell nuclei were stained with 4,6-diamidino-2-phenylindole (DAPI, fluorescent blue). Fluorescent microscopy images were acquired using a Leica confocal laser scanning microscope (Leica, Wetzlar, Germany).

Immunofluorescence staining assays were performed on MCF-7 cells to further characterize the effects of compound **B6** on microtubule dynamic equilibrium. As shown in Figure 5, the microtubule networks in the DMSO-treated control group presented a normal, ordered arrangement, where thin fibrous microtubules (red fluorescence) surrounded the cell nuclei (blue fluorescence). However, a 24 h treatment with compound **B6** at three dose levels (0.1, 1, and 10 μM) resulted in the disruption of the cytoplasmic microtubule network structure. These results indicate that compound **B6** dose-dependently leads to disintegration of the microtubule network in MCF-7 cells.

#### 2.2.5. Inhibited the Colony Formation of Cancer Cells

**Figure 6:**
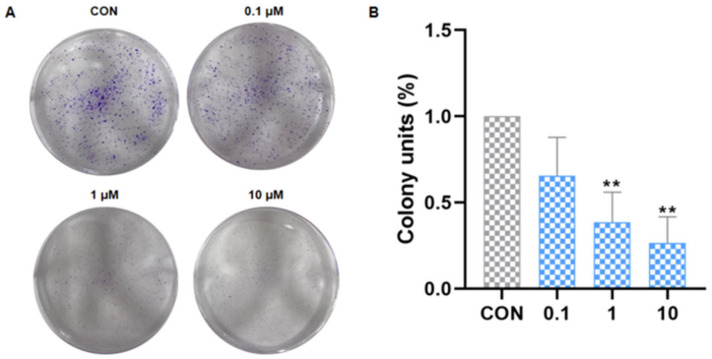
(**A**) Compound **B6** inhibited the colony formation of MCF-7 cells at different concentrations in a dose-dependent manner. (**B**) Number of colonies (*n* = 3, ** *p* < 0.01, when compared with the control group).

A colony formation assay was performed to evaluate the long-term inhibitory effects (14 days) of **B6** against MCF-7 cells at concentrations ranging from 0.1 to 10 μM, fluctuating around its IC_50_ value. The results showed that compound **B6** significantly reduced the number and area of MCF-7 cell colonies at a dose-dependent level (Figure 6). At a concentration of 10 μM, **B6** completely inhibited the clone formation and cell proliferation potential of MCF-7 cells.

#### 2.2.6. Inhibition of Cancer Cell Migration and Invasion

**Figure 7:**
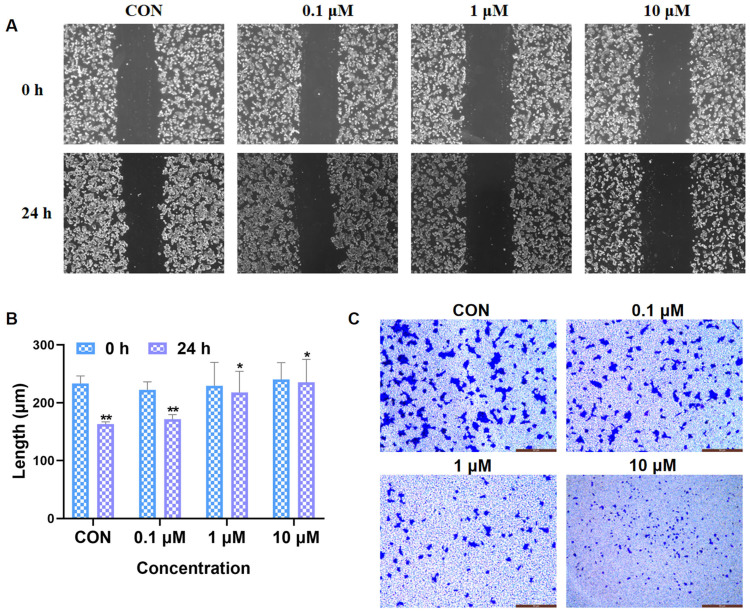
Effect of compound **B6** on MCF-7 cell migration and invasion. (**A**) Representative micrographs of the wound region in the scratch wound healing assay. Images were captured at 0 h and 24 h following treatment with compound **B6** at concentrations of 0, 0.1, 1, and 10 μM. (**B**) Histogram plots illustrate the scratch length measurements at 0 h and 24 h post-treatment with 0, 0.1, 1, and 10 μM compound **B6**. (**C**) The impact of compound **B6** on MCF-7 cell invasion was evaluated using Transwell assays. Images were acquired at 24 h after treatment with compound **B6** at doses of 0, 0.1, 1, and 10 μM (*n* = 3, * *p* < 0.05, and ** *p* < 0.01 vs. the control group).

Tubulin and microtubules play a crucial role in cell migration and motility. Therefore, we performed a wound healing assay to confirm the effects of compound **B6** in cell migration via microtubule destabilization. As shown in Figure 7A,B, the average wound closure rates of compound **B6** on MCF-7 cells after 24 h were 14.19 ± 4.93%, 7.70 ± 3.16% and 4.83 ± 2.11% (Figure 7B) at concentrations of 0.1, 1, and 10 μM, respectively. Compared with the untreated control group, the wound closure rate was drastically decreased by compound **B6** treatment, and compound **B6** was significantly cytotoxic to MCF-7 cells, rather than inhibiting wound closure at a concentration of 10 μM (Figure 7A). In addition, the results of the Transwell migration assay showed that the control group had more cells on the lower surface of the Transwell chamber (Figure 7C). In contrast, fewer cells invaded the lower surface of the Transwell chambers in the compound **B6** group. In particular, at a concentration of 10 μM, almost no cells were present on the lower surface of the Transwell chamber. This suggests that compound **B6** inhibits the migration of MCF-7 cells.

In conclusion, compound **B6** has the property of anti-invasion and migration of MCF-7 cells.

#### 2.2.7. Cell Cycle Analysis

**Figure 8:**
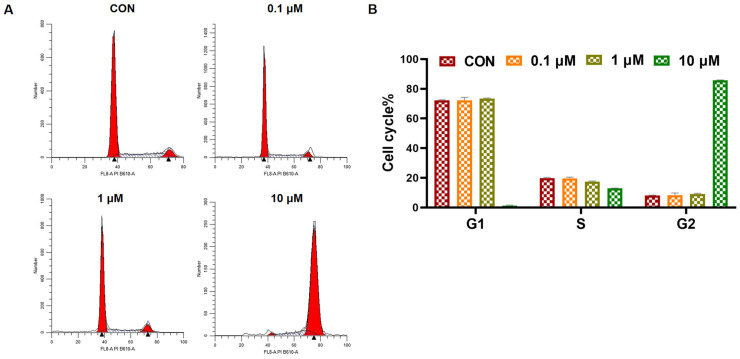
Cell cycle arrestment elicited by compound **B6**. (**A**) Compound **B6** triggered G2/M phase arrest in MCF-7 cells. (**B**) Histogram plots illustrate the proportion of cells distributed across different cell cycle phases following treatment with compound **B6**. All data are presented as the mean ± standard deviation derived from three independent experimental replicates.

Inhibitors of microtubule protein polymerization disrupt cellular mitosis and induce cell cycle arrest [32]. Therefore, flow cytometry analysis was conducted to assess the effect of compound **B6** on the cell cycle of MCF-7 cells. As shown in Figure 8, compared with the blank control group (in which 11.20% of cells were arrested in the G2/M phase), treatment of MCF-7 cells with different concentrations of compound **B6** (0.1 μM, 1 μM, and 10 μM) for 48 h resulted in an increase in the percentage of cells arrested in the G2/M phase from 12.77% to 84.47%. These results are consistent with the fundamental characteristics of CBSIs.

#### 2.2.8. Cell Apoptosis Analysis

**Figure 9:**
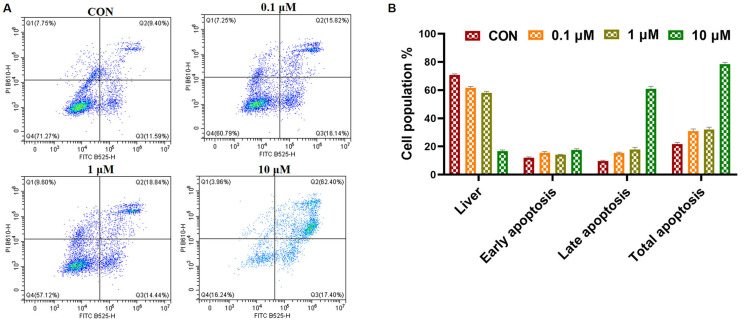
Effects of compound **B6** on MCF-7 cell apoptosis. (**A**) Compound **B6** induced apoptosis in MCF-7 cells. The percentages of cells in each stage of cell apoptosis were quantified by flow cytometry: necrosis cells (upper left quadrant); late apoptotic cells (upper right quadrant); live cells (bottom left quadrant); and early apoptotic cells (bottom right quadrant). (**B**) Histograms display the percentage of cell apoptosis. Data are expressed as mean ± standard deviation from three independent experiments.

It is well-established that mitotic arrest of tumor cells triggered by tubulin polymerization inhibitors is generally linked to cellular apoptosis. Accordingly, an Annexin V FITC/propidium iodide (PI) double-staining assay was conducted on MCF-7 cells to assess whether compound **B6** was capable of eliciting apoptotic responses in cancer cells. As shown in Figure 9, after treating MCF-7 cells with different concentrations of compound **B6** (0.1 μM, 1 μM, and 10 μM) for 48 h, the total proportion of apoptotic cells increased from 20.99% (control group) to 31.96% (0.1 μM), 33.28% (1 μM), and 79.80% (10 μM). These results indicate that compound **B6** could effectively induce the apoptosis of MCF-7 cells.

#### 2.2.9. Molecular Modeling Studies with Compound **B6** and Tubulin

**Figure 10:**
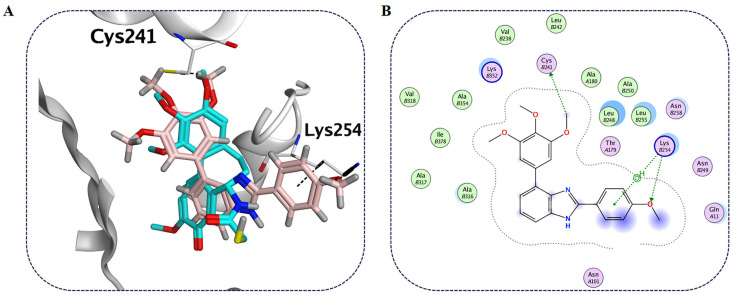
Molecular docking model (PDB ID: 1SA0). (**A**) 3D docking simulation of compound **B6** at the tubulin colchicine binding site. (**B**) 2D docking simulation of compound **B6** at the tubulin colchicine binding site.

To investigate the possible binding mode for this series of compounds, molecular docking simulation of compound **B6** in the active cavity of tubulin (PDB: 1SA0) was performed using MOE (Molecular Operating Environment) 2022.02. As depicted in Figure 10, compound **B6** adopted pretty similar location with colchicine in the colchicine-binding site, which was located on the interface between ɑ/β-subunits and extended slightly out toward β-subunit. Furthermore, compound **B6** was docked to the binding site of tubulin, surrounded by residues Lys352, Ala316, Cys241, Thr179, and Lys254. The methoxy of compound **B6** could form a hydrogen bond with Lys254. Besides the hydrogen interactions, there were some hydrophobic interactions with the surrounding residues. The hydrogen bonds and hydrophobic interactions played a key role in the binding of compound **B6** with tubulin.

#### 2.2.10. Molecular Dynamics Studies with Compound **B6** and Tubulin

To explore the stability of the compound-**B6**–tubulin complexes and the oscillations and conformational changes occurring during protein–ligand interactions, molecular dynamics stimulations were performed. Analysis of the root mean square deviation (RMSD) for the protein–ligand complex revealed that the system’s conformation progressively approached equilibrium and maintained fluctuations as the simulation progressed, indicating favorable kinetic stability. Concurrently, the number of hydrogen bond interactions between the two components exhibited dynamic fluctuations, reflecting the time-dependent dynamic plasticity of interactions at the binding interface. Furthermore, the root mean square fluctuation (RMSF) analysis of ligand atoms revealed significantly increased thermal motion flexibility in certain regions, reflecting conformational flexibility differences in the bound state. Analysis of protein residue RMSF and β-factor data revealed that the conformational flexibility in certain residue regions aligns with experimental thermal motion trends, with marked differences in residue flexibility observed across distinct secondary structural domains. Collectively, these findings systematically elucidate the dynamic structural characteristics of compound **B6**’s interaction with tubulin protein across dimensions including overall structural stability, intermolecular interactions, and local conformational flexibility. This provides a kinetic foundation for subsequent investigations into its molecular mechanism of action.

#### 2.2.11. In Vivo Antitumor Efficacy

Tubulin is highly conserved across virtually all cell types, rendering tubulin inhibitors typically broad-spectrum anticancer agents. The B16-F10 cell line exhibits high invasiveness, rapid growth, and readily forms subcutaneous xenografts. Consequently, the in vivo antitumor efficacy of compound **B6** was further evaluated in the mice melanoma tumor model, established by subcutaneous inoculation of B16-F10 cells into the mice. Preliminary experiments were also carried out to determine the cytotoxicity of **B6** in this cell line. The results of the CCK8 tests indicated an IC_50_ of 0.40 μM.

**Figure 11:**
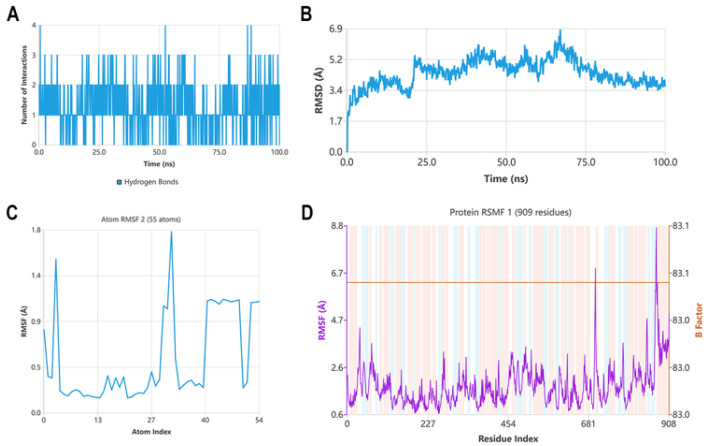
Molecular dynamics studies with compound **B6** and tubulin. (**A**) Hydrogen bond interactions in the 100 ns molecular dynamics simulation. The plot shows the time evolution of the number of hydrogen bond interactions between the ligand and protein from 0 to 100 ns. The *y*-axis represents the number of hydrogen bond interactions, and the *x*-axis denotes the simulation time in nanoseconds (ns). (**B**) Root means square deviation (RMSD) of the protein–ligand complex in 100 ns MD simulation. Time-dependent RMSD (in Å) of the complex is plotted against simulation time (ns). RMSD values reflect the structural deviation of the complex from its initial conformation during the 100 ns molecular dynamics simulation. (**C**) Root means square fluctuation (RMSF) of ligand atoms (55 atoms) from 100 ns MD simulation. RMSF values (in Å) for individual ligand atoms (atom index 0 to 54) are shown, indicating the flexibility of each atom in the ligand during the simulation. (**D**) Root means square fluctuation (RMSF) of protein residues (909 residues) and corresponding B-factor analysis. The purple curve represents RMSF values (in Å) of protein residues (residue index 0 to 908) from the 100 ns simulation, reflecting residue-level flexibility. The orange axis and line show the B-factor values, and the colored background (blue/pink) denotes secondary structure regions of the protein (β-sheets/α-helices, respectively).

**Figure 12:**
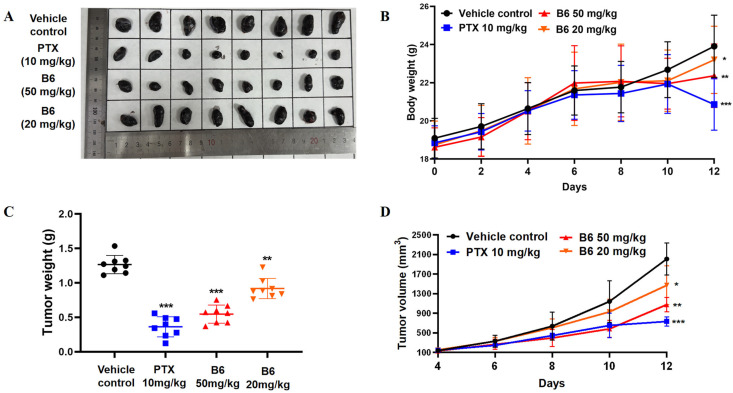
Compound **B6** exerts inhibitory effects on melanoma tumor growth in vivo. Mice were treated with vehicle control, paclitaxel (PTX, 10 mg/kg daily), or compound **B6** (20/50 mg/kg daily) for a 12-day period, after which the animals were euthanized, and the tumors were excised and weighed. (**A**) Representative images of tumors collected from mice on day 12 post-treatment initiation. (**B**) Variations in body weight of mice throughout the treatment course. (**C**) Weights of dissected tumors in each experimental group. (**D**) Dynamic changes in tumor volume of mice during treatment. * *p* < 0.05, ** *p* < 0.01, *** *p* < 0.001 vs. the vehicle control group.

**Figure 13:**
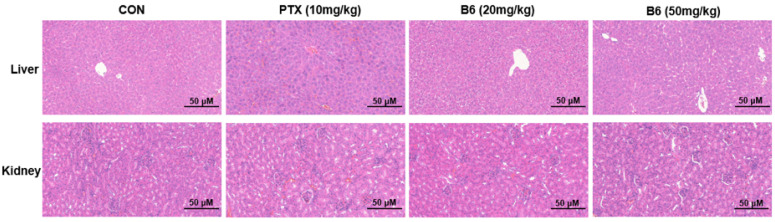
Histopathological sections of key visceral tissues (liver and kidney) were harvested from melanoma-bearing mice. The isolated tissues were subjected to hematoxylin and eosin (H&E) staining, and representative histological micrographs were acquired.

BALB/c mice transplanted with melanoma xenografts were administered vehicle control, paclitaxel (PTX, 10 mg/kg), or compound **B6** (20 and 50 mg/kg) by intraperitoneal (i.p.) injection at 2-day intervals over a 12-day treatment period. As depicted in Figure 11, compound **B6** exhibited potent in vivo anti-tumor activity, with a tumor growth inhibition (TGI) rate of 70.21% at the dose of 50 mg/kg—this efficacy was marginally lower than that of PTX (74.19%) at 10 mg/kg. Additionally, compound **B6** did not induce notable body weight reduction at 20 mg/kg, suggesting that the compound possessed favorable tolerability at low-dose regimens (Figure 12B). Furthermore, the hematoxylin and eosin (H&E) staining of liver and kidney showed that compound **B6** had no significant toxic effects on the major organs of mice (Figure 13).

## 3. Materials and Methods

### 3.1. General Chemistry Methods

General chemicals, supplied by Shanghai Taitan Technology Co., Ltd. (Shanghai, China), were commercially available in analytical purity and were not further purified. The melting points were measured with an SGW^®^ X-4 Amicroscopic (Shanghai INESA Physico, Shanghai, China) melting point apparatus. ^1^H and ^13^C NMR spectra were recorded on a Bruker Avance (Agilent, Shanghai, China) at 400 MHz and 500 MHz using CDCl_3_ or DMSO-*d6* as the solvent and tetramethylsilane as the internal standard. High-resolution mass spectra (HRMS) were measured using an Agilent Technologies 6538 UHD accurate-Mass Q-TOF MS spectrometer (Agilent, Shanghai, China) with electrospray ionization.

Synthesis of 2-amino-3′,4′,5′-trimethoxy-[1,1′-biphenyl]-3-carboxamide (**2**)

To a solution of 2-amino-3-bromobenzamide (200 mg, 0.93 mmol) in 8 mL of THF and 2 mL of H_2_O were added (3,4,5-trimethoxyphenyl) boronic acid (219 mg, 1.03 mmol), K_2_CO_3_ (386 mg, 2.79 mmol), and catalytic equivalent of 1,1′-*bis*(diphenylphosphino)ferrocene-palladium(II) dichloride dichloromethane complex. The mixture was stirred at 80 °C under a nitrogen atmosphere for 5 h. Then, the solvent was removed in vacuo, and the residue was diluted by H_2_O and extracted with EtOAc (3 × 10 mL). The combined organic layer was then washed with brine, dried over anhydrous Na_2_SO_4_, and concentrated in vacuo to provide the crude product, which was directly subjected to silica gel column chromatography eluted with petroleum PE/EtOAc (3/1, *v*/*v*) to give intermediate **2** as a yellow solid; yield: 38.2%, m.p. 154.0–155.7 °C; ^1^H NMR (500 MHz, DMSO-*d*_6_) (Figure S1) δ 7.85 (s, 1H), 7.57 (dd, *J* = 8.0, 1.5 Hz, 1H), 7.21 (s, 1H), 7.14 (dd, *J* = 7.3, 1.5 Hz, 1H), 6.65 (s, 2H), 6.61 (t, *J* = 7.7 Hz, 1H), 6.33 (s, 2H), 3.81 (s, 6H), 3.72 (s, 3H). ^13^C NMR (126 MHz, DMSO-*d*_6_) (Figure S2) δ 172.02, 153.59, 147.03, 137.04, 134.74, 133.36, 128.73, 128.46, 115.16, 115.04, 106.63, 60.44, 56.30. LCMS (ESI), *m*/*z*: calcd. for C_14_H_9_BrF_3_N_2_, [M+H]^+^ 303.1; found 303.0.

#### 3.1.1. General Procedures for the Preparation of Compounds **A0**–**A19**

To a 20 mL microwave reaction tube was added intermediate **2** (0.50 mmol), aromatic aldehyde (0.60 mmol), and DMSO (8 mL). The tube was sealed and the reaction was heated within a microwave reactor at 120 °C for 6 h. Then, the solvent was diluted by H_2_O and extracted with EtOAc (3 × 10 mL). The combined organic layer was then washed with brine, dried over anhydrous Na_2_SO_4_, and concentrated in vacuo to provide the crude product, which was directly subjected to silica gel column chromatography eluted with petroleum PE/EtOAc (1/1, *v*/*v*) to give corresponding pure compounds **A0**–**A19**.

2-(1*H*-indol-5-yl)-8-(3,4,5-trimethoxyphenyl)quinazolin-4(3*H*)-one(**A0**).

Yellow solid. Yield 25.3%. Mp 173.5–175.0 °C. ^1^H NMR (600 MHz, CDCl_3_) δ 8.60 (s, 1H), 8.00 (dd, *J* = 7.8, 1.4 Hz, 1H), 7.44–7.41 (m, 2H), 7.26 (s, 1H), 6.95 (t, *J* = 7.6 Hz, 1H), 6.60 (s, 2H), 6.56–6.54 (m, 1H), 5.98 (d, *J* = 12.4 Hz, 1H), 5.90 (s, 1H), 3.83 (s, 3H), 3.80 (s, 6H).^13^C NMR (151 MHz, CDCl_3_) δ 153.67, 144.77, 134.47, 128.18, 127.81, 125.69, 120.74, 120.06, 118.93, 111.90, 111.12, 105.89, 102.88, 60.88, 56.15. HRMS (ESI), *m*/*z*: calcd. for C_25_H_22_N_3_O_4_, [M+H]^+^ 428.1605; found: 428.1609.

2-Phenyl-8-(3,4,5-trimethoxyphenyl)quinazolin-4(3*H*)-one (**A1**).

Yellow solid. Yield 29.4%. Mp 189.2–191.0 °C. ^1^H NMR (500 MHz, DMSO-*d*_6_) δ 12.65 (s, 1H), 8.21–8.20 (m, 1H), 8.19–8.18 (m, 1H), 7.96 (dd, *J* = 7.5, 1.5 Hz, 1H), 7.60 (d, *J* = 7.8 Hz, 1H), 7.58–7.56 (m, 1H), 7.56 (s, 1H), 7.54 (s, 1H), 7.41–7.38 (m, 1H), 7.08 (s, 2H), 3.85 (s, 6H), 3.76 (s, 3H). ^13^C NMR (126 MHz, DMSO-*d*_6_) δ 162.93, 152.73, 151.57, 146.04, 138.92, 137.54, 135.68, 134.25, 133.34, 131.90, 129.12, 129.05, 128.12, 127.06, 126.85, 126.25, 125.72, 122.19, 108.80, 60.60, 56.39. HRMS (ESI), *m*/*z*: calcd. for C_23_H_21_N_2_O_4_, [M+H]^+^ 389.0556; found: 389.0551.

2-(Pyridin-4-yl)-8-(3,4,5-trimethoxyphenyl)quinazolin-4(3*H*)-one (**A2**).

Yellow solid. Yield 35.6%. Mp 167.3–168.9 °C. ^1^H NMR (500 MHz, DMSO-*d*_6_) δ 12.87 (s, 1H), 8.79 (d, *J* = 6.0 Hz, 2H), 8.21 (dd, *J* = 7.9, 1.5 Hz, 1H), 8.09 (dd, *J* = 4.6, 1.5 Hz, 2H), 7.98 (dd, *J* = 7.5, 1.5 Hz, 1H), 7.64 (t, *J* = 7.7 Hz, 1H), 7.06 (s, 2H), 3.85 (s, 6H), 3.77 (s, 3H). ^13^C NMR (126 MHz, DMSO-*d*_6_) δ 162.71, 152.78, 150.71, 149.77, 145.54, 140.61, 139.33, 137.60, 135.85, 134.01, 127.68, 125.75, 122.70, 121.86, 108.77, 60.61, 56.42. HRMS (ESI), *m*/*z*: calcd. for C_22_H_18_N_3_O_4_, [M-H]^−^ 388.1302; found: 388.1307.

2-(4-Nitrophenyl)-8-(3,4,5-trimethoxyphenyl)quinazolin-4(3*H*)-one (**A3**).

Yellow solid. Yield 10.6%. Mp 158.1–159.1 °C. ^1^H NMR (500 MHz, DMSO-*d*_6_) δ 12.93 (s, 1H), 8.42–8.37 (m, 4H), 8.22 (dd, *J* = 7.9, 1.5 Hz, 1H), 7.99 (dd, *J* = 7.5, 1.5 Hz, 1H), 7.65 (t, *J* = 7.7 Hz, 1H), 7.06 (s, 2H), 3.85 (s, 6H), 3.76 (s, 3H). ^13^C NMR (126 MHz, DMSO-*d*_6_) (Figure S10) δ 162.73, 152.82, 149.45, 139.34, 139.21, 135.92, 134.06, 129.56, 127.64, 125.75, 124.17, 108.75, 60.60, 56.48. HRMS (ESI), *m*/*z*: calcd. for C_23_H_18_N_3_O_6_, [M-H]^−^ 432.1196; found: 432.1213.

4-(4-Oxo-8-(3,4,5-trimethoxyphenyl)-3,4-dihydroquinazolin-2-yl)benzonitrile (**A4**).

Yellow solid. Yield 66.5%. Mp 167.1–169.2 °C. ^1^H NMR (500 MHz, DMSO-*d*_6_) δ 12.84 (s, 1H), 8.33 (d, *J* = 8.2 Hz, 2H), 8.21 (d, *J* = 7.8 Hz, 1H), 8.05 (d, *J* = 8.2 Hz, 2H), 7.98 (d, *J* = 7.1 Hz, 1H), 7.63 (t, *J* = 7.6 Hz, 1H), 7.05 (s, 2H), 3.84 (s, 6H), 3.76 (s, 3H). ^13^C NMR (126 MHz, DMSO-*d*_6_) δ 152.79, 150.20, 145.66, 139.25, 137.50, 135.79, 134.07, 133.04, 128.87, 127.56, 125.74, 122.45, 118.79, 108.78, 60.60, 56.46. HRMS (ESI), *m*/*z*: calcd. for C_24_H_18_N_3_O_4_, [M-H]^−^ 412.1302; found: 412.1318.

2-(2-(Trifluoromethyl)phenyl)-8-(3,4,5-trimethoxyphenyl)quinazolin-4(3*H*)-one (**A5**).

Yellow solid. Yield 16.2%. Mp 165.9–166.6 °C. ^1^H NMR (500 MHz, DMSO-*d*_6_) δ 12.76 (s, 1H), 8.22 (dd, *J* = 7.9, 1.6 Hz, 1H), 7.92 (dd, *J* = 7.6, 1.6 Hz, 1H), 7.82 (d, *J* = 7.3 Hz, 1H), 7.80–7.77 (m, 2H), 7.76–7.74 (m, 1H), 7.63 (t, *J* = 7.7 Hz, 1H), 6.88 (s, 2H), 3.74 (s, 6H), 3.68 (s, 3H). ^13^C NMR (126 MHz, DMSO-*d*_6_) δ 161.85, 152.64, 152.15, 145.81, 139.33, 137.44, 135.89, 134.16, 132.92, 131.13, 130.86, 127.24, 126.75, 126.71, 125.70, 122.37, 108.55, 60.42, 56.16, 21.21, 14.54. HRMS (ESI), *m*/*z*: calcd. for C_24_H_18_F_3_N_2_O_4_, [M-H]^−^ 455.1224; found: 455.1212.

2-(2-Methylphenyl)-8-(3,4,5-trimethoxyphenyl)quinazolin-4(3*H*)-one (**A6**).

White solid. Yield 25.4%. Mp 171.2–174.1 °C. ^1^H NMR (500 MHz, DMSO-*d*_6_) δ 12.53 (s, 1H), 8.22–8.19 (m, 1H), 7.89 (dd, *J* = 7.4, 1.6 Hz, 1H), 7.61–7.58 (m, 1H), 7.57–7.55 (m, 1H), 7.41 (td, *J* = 7.8, 1.3 Hz, 1H), 7.34–7.30 (m, 2H), 6.92 (s, 2H), 3.79 (s, 6H), 3.70 (s, 3H), 2.39 (s, 3H). ^13^C NMR (126 MHz, DMSO-*d*_6_) δ 162.47, 153.96, 152.62, 146.28, 139.33, 137.38, 136.66, 135.56, 134.77, 134.46, 131.02, 130.41, 129.76, 126.72, 126.13, 125.65, 122.08, 108.64, 60.48, 56.32, 20.35. HRMS (ESI), *m*/*z*: calcd. for C_24_H_21_N_2_O_4_, [M-H]^−^ 401.1506; found: 401.1506.

2-(2,6-Dimethoxyphenyl)-8-(3,4,5-trimethoxyphenyl)quinazolin-4(3*H*)-one (**A7**).

Yellow solid. Yield 12.1%. Mp 128.1–130.2 °C. ^1^H NMR (500 MHz, DMSO-*d*_6_) δ 12.40 (s, 1H), 8.17 (dd, *J* = 8.0, 1.6 Hz, 1H), 7.92 (dd, *J* = 7.5, 1.6 Hz, 1H), 7.59 (t, *J* = 7.7 Hz, 1H), 7.43 (t, *J* = 8.5 Hz, 1H), 6.96 (s, 2H), 6.78 (d, *J* = 8.5 Hz, 2H), 3.76 (s, 6H), 3.73 (s, 6H), 3.69 (s, 3H). ^13^C NMR (126 MHz, DMSO-*d*_6_) δ 162.43, 158.27, 152.58, 149.67, 146.44, 138.62, 137.32, 135.27, 134.03, 131.84, 126.78, 125.55, 122.43, 113.67, 108.59, 104.56, 60.43, 56.26, 56.09. HRMS (ESI), *m*/*z*: calcd. for C_25_H_25_N_2_O_6_, [M+H]^+^ 449.1708; found: 449.1707.

2-(Naphthalen-1-yl)-8-(3,4,5-trimethoxyphenyl)quinazolin-4(3*H*)-one (**A8**).

Yellow solid. Yield 31.4%. Mp 161.5–162.3 °C. ^1^H NMR (500 MHz, DMSO-*d*_6_) δ 12.75 (s, 1H), 8.29 (d, *J* = 8.4 Hz, 1H), 8.26 (dd, *J* = 8.0, 1.6 Hz, 1H), 8.11 (d, *J* = 8.3 Hz, 1H), 8.04 (d, *J* = 8.0 Hz, 1H), 7.93 (dd, *J* = 7.5, 1.6 Hz, 1H), 7.81 (dd, *J* = 7.1, 1.0 Hz, 1H), 7.64 (dd, *J* = 7.3, 1.3 Hz, 1H), 7.62 (dd, *J* = 3.3, 1.2 Hz, 1H), 7.61–7.58 (m, 1H), 7.56–7.52 (m, 1H), 6.97 (s, 2H), 3.70 (s, 6H), 3.64 (s, 3H). ^13^C NMR (126 MHz, DMSO-*d*_6_) δ 162.60, 153.32, 152.61, 146.29, 139.38, 137.28, 135.61, 134.43, 133.60, 132.45, 130.83, 130.70, 128.80, 128.15, 127.45, 126.87, 126.81, 125.86, 125.71, 125.52, 122.36, 108.57, 60.39, 56.29. HRMS (ESI), *m*/*z*: calcd. for C_27_H_21_N_2_O_4_, [M-H]^−^ 437.1507; found: 437.1519.

2-(2-Methoxynaphthalen-1-yl)-8-(3,4,5-trimethoxyphenyl)quinazolin-4(3*H*)-one (**A9**).

Yellow solid. Yield 39.3%. Mp 186.4–187.7 °C. ^1^H NMR (500 MHz, DMSO-*d*_6_) δ 12.60 (s, 1H), 8.26 (dd, *J* = 8.0, 1.6 Hz, 1H), 8.13 (d, *J* = 9.1 Hz, 1H), 8.00–7.96 (m, 1H), 7.94 (dd, *J* = 7.5, 1.6 Hz, 1H), 7.80 (d, *J* = 7.9 Hz, 1H), 7.64 (t, *J* = 7.7 Hz, 1H), 7.58 (d, *J* = 9.2 Hz, 1H), 7.49–7.46 (m, 1H), 7.44–7.40 (m, 1H), 6.93 (s, 2H), 3.90 (s, 3H), 3.62 (s, 6H), 3.61 (s, 3H). ^13^C NMR (126 MHz, DMSO-*d*_6_) δ 162.54, 155.16, 153.42, 152.55, 151.28, 146.47, 139.17, 137.19, 135.48, 134.23, 132.27, 131.99, 128.56, 127.96, 126.92, 125.67, 124.29, 124.23, 122.57, 117.92, 113.97, 108.48, 106.10, 60.34, 56.77, 56.09, 55.38. HRMS (ESI), *m*/*z*: calcd. for C_28_H_23_N_2_O_5_, [M-H]^−^ 467.1612; found: 467.1607.

2-Cyclopropyl-8-(3,4,5-trimethoxyphenyl)quinazolin-4(3*H*)-one (**A10**).

Yellow solid. Yield 29.7%. Mp 132.1–133.2 °C. ^1^H NMR (500 MHz, DMSO-*d*_6_) δ 12.54 (s, 1H), 8.08 (dd, *J* = 7.9, 1.6 Hz, 1H), 7.84 (dd, *J* = 7.4, 1.6 Hz, 1H), 7.45 (t, *J* = 7.7 Hz, 1H), 6.94 (s, 2H), 3.83 (s, 6H), 3.73 (s, 3H), 1.05–1.00 (m, 4H). ^13^C NMR (126 MHz, DMSO-*d*_6_) δ 162.27, 158.58, 152.53, 146.42, 137.69, 137.27, 135.41, 134.26, 125.63, 125.55, 121.81, 108.51, 60.53, 56.26, 14.10, 9.97. HRMS (ESI), *m*/*z*: calcd. for C_20_H_19_N_2_O_4_, [M-H]^−^ 351.1350; found: 351.1358.

2-(4-Methoxyphenyl)-8-(3,4,5-trimethoxyphenyl)quinazolin-4(3*H*)-one (**A11**).

White solid. Yield 12.7%. Mp 258.4–259.0 °C. ^1^H NMR (400 MHz, DMSO-*d*_6_) δ 12.50 (s, 1H), 8.19–8.17 (m, 2H), 8.17–8.14 (m, 1H), 7.92 (dd, *J* = 7.4, 1.5 Hz, 1H), 7.54 (t, *J* = 7.7 Hz, 1H), 7.10–7.07 (m, 2H), 7.06 (s, 2H), 3.85 (s, 6H), 3.84 (s, 3H), 3.76 (s, 3H). ^13^C NMR (101 MHz, DMSO-*d*_6_) δ 162.98, 162.39, 152.71, 151.18, 146.24, 138.65, 137.46, 135.63, 134.37, 129.83, 126.36, 125.70, 125.43, 121.88, 114.49, 108.72, 60.61, 56.39, 55.97. HRMS (ESI), *m*/*z*: calcd. for C_24_H_23_N_2_O_5_, [M+H]^+^ 419.1601; found: 419.1613.

2-(4-Tert-butylphenyl)-8-(3,4,5-trimethoxyphenyl)quinazolin-4(3*H*)-one (**A12**).

Yellow solid. Yield 25.7%. Mp 172.3–172.8 °C. ^1^H NMR (400 MHz, CDCl_3_) δ 10.15 (s, 1H), 8.35 (d, *J* = 7.6 Hz, 1H), 8.12 (d, *J* = 8.4 Hz, 2H), 7.91 (d, *J* = 7.0 Hz, 1H), 7.58 (d, *J* = 7.8 Hz, 1H), 7.55 (d, *J* = 8.3 Hz, 2H), 6.99 (s, 2H), 3.97 (s, 3H), 3.94 (s, 6H), 1.37 (s, 9H). ^13^C NMR (101 MHz, CDCl_3_) δ 165.05, 155.83, 152.76, 149.88, 146.71, 139.57, 136.23, 133.82, 129.30, 127.00, 126.81, 126.19, 125.94, 120.60, 108.32, 61.03, 56.29, 35.10, 31.14. HRMS (ESI), *m*/*z*: calcd. for C_27_H_29_N_2_O_4_, [M+H]^+^ 445.2122; found: 445.2115.

2-Cyclobutyl-8-(3,4,5-trimethoxyphenyl)quinazolin-4(3*H*)-one (**A13**).

Yellow solid. Yield 13.2%. Mp 220.1–221.3 °C. ^1^H NMR (400 MHz, DMSO-*d*_6_) δ 12.13 (s, 1H), 8.09 (dd, *J* = 7.9, 1.5 Hz, 1H), 7.89 (dd, *J* = 7.5, 1.6 Hz, 1H), 7.51 (t, *J* = 7.7 Hz, 1H), 7.05 (s, 2H), 3.83 (s, 6H), 3.73 (s, 3H), 2.43–2.32 (m, 2H), 2.26–2.17 (m, 2H), 2.05–1.86 (m, 2H), 1.84–1.75 (m, 1H). ^13^C NMR (101 MHz, DMSO-*d*_6_) δ 162.49, 158.66, 152.58, 146.17, 138.26, 137.36, 135.41, 134.26, 126.22, 125.57, 122.04, 108.72, 60.55, 56.25, 26.18, 18.01. HRMS (ESI), *m*/*z*: calcd. for C_21_H_23_N_2_O_4_, [M+H]^+^ 367.1652; found: 367.1672.

2-(2-Fluoropyridin-3-yl)-8-(3,4,5-trimethoxyphenyl)quinazolin-4(3*H*)-one (**A14**).

Yellow solid. Yield 14.7%. Mp 191.0–191.5 °C. ^1^H NMR (400 MHz, DMSO-*d*_6_) δ 12.77 (s, 1H), 8.44–8.41 (m, 1H), 8.37–8.31 (m, 1H), 8.21 (dd, *J* = 7.9, 1.5 Hz, 1H), 7.96 (dd, *J* = 7.5, 1.5 Hz, 1H), 7.64 (t, *J* = 7.7 Hz, 1H), 7.58–7.54 (m, 1H), 7.01 (s, 2H), 3.82 (s, 6H), 3.71 (s, 3H). ^13^C NMR (101 MHz, DMSO-*d*_6_) δ 162.12, 161.28, 158.90, 152.71, 150.21, 150.06, 148.25, 148.19, 145.85, 142.75, 139.18, 137.52, 135.82, 133.94, 127.54, 125.69, 122.67, 122.62, 122.37, 117.98, 117.70, 108.70, 60.50, 56.35, 55.37. HRMS (ESI), *m*/*z*: calcd. for C_22_H_19_FN_3_O_4_, [M+H]^+^ 408.1355; found: 408.1370.

2-(1*H*-pyrazol-4-yl)-8-(3,4,5-trimethoxyphenyl)quinazolin-4(3*H*)-one (**A15**).

Yellow solid. Yield 13.5%. Mp 286.5–287.1 °C. ^1^H NMR (400 MHz, DMSO-*d*_6_) δ 13.33 (s, 1H), 12.43 (s, 1H), 8.49 (s, 1H), 8.13 (dd, *J* = 7.9, 1.5 Hz, 1H), 8.08 (s, 1H), 7.89 (dd, *J* = 7.5, 1.5 Hz, 1H), 7.50 (t, *J* = 7.7 Hz, 1H), 7.03 (s, 2H), 3.84 (s, 6H), 3.76 (s, 3H). ^13^C NMR (101 MHz, DMSO-*d*_6_) δ 162.74, 152.67, 147.51, 146.61, 138.13, 137.47, 135.56, 134.30, 125.90, 125.68, 121.82, 117.00, 108.74, 60.59, 56.39. HRMS (ESI), *m*/*z*: calcd. for C_20_H_19_N_4_O_4_, [M+H]^+^ 379.1401; found: 379.1399.

2-(1-Methyl-1*H*-indol-5-yl)-8-(3,4,5-trimethoxyphenyl)quinazolin-4(3*H*)-one (**A16**).

Yellow solid. Yield 12.0%. Mp 232.4–233.0 °C. ^1^H NMR (400 MHz, CDCl_3_) δ 8.49 (s, 1H), 8.37 (d, *J* = 7.8 Hz, 1H), 8.08 (d, *J* = 7.8 Hz, 1H), 7.88 (d, *J* = 6.5 Hz, 1H), 7.52 (t, *J* = 7.6 Hz, 1H), 7.42 (d, *J* = 8.7 Hz, 1H), 7.15 (d, *J* = 3.0 Hz, 1H), 7.08 (s, 2H), 6.62 (d, *J* = 2.9 Hz, 1H), 3.98 (s, 3H), 3.96 (s, 6H), 3.85 (s, 3H). ^13^C NMR (101 MHz, CDCl_3_) δ 163.94, 152.72, 151.34, 146.87, 139.04, 138.56, 135.59, 134.27, 130.57, 128.62, 125.96, 123.82, 121.30, 120.83, 120.54, 109.83, 108.39, 102.55, 61.02, 56.26, 33.11, 29.72. HRMS (ESI), *m*/*z*: calcd. for C_26_H_24_N_3_O_4_, [M+H]^+^ 442.1761; found: 442.1773.

2-(Oxazol-4-yl)-8-(3,4,5-trimethoxyphenyl)quinazolin-4(3*H*)-one (**A17**).

White solid. Yield 17.5%. Mp 203.1–204.6 °C. ^1^H NMR (400 MHz, DMSO-*d*_6_) δ 8.81 (d, *J* = 0.5 Hz, 1H), 8.63 (d, *J* = 0.8 Hz, 1H), 8.16 (dd, *J* = 7.9, 1.5 Hz, 1H), 7.96 (dd, *J* = 7.5, 1.5 Hz, 1H), 7.58 (t, *J* = 7.7 Hz, 1H), 7.10 (s, 2H), 3.86 (s, 6H), 3.74 (s, 3H). ^13^C NMR (101 MHz, DMSO-*d*_6_) δ 166.85, 158.27, 157.47, 150.67, 150.03, 146.56, 143.27, 142.26, 140.39, 139.84, 138.55, 131.81, 130.49, 127.49, 113.52, 65.28, 61.13. HRMS (ESI), *m*/*z*: calcd. for C_20_H_18_N_3_O_5_, [M+H]^+^ 380.1241; found: 380.1248.

2-(Thiazol-5-yl)-8-(3,4,5-trimethoxyphenyl)quinazolin-4(3*H*)-one (**A18**).

White solid. Yield 29.9%. Mp 286.7–287.3 °C. ^1^H NMR (400 MHz, DMSO-*d*_6_) δ 9.30 (s, 1H), 8.95 (s, 1H), 8.20 (dd, *J* = 7.9, 1.4 Hz, 1H), 7.98 (dd, *J* = 7.5, 1.5 Hz, 1H), 7.62 (t, *J* = 7.7 Hz, 1H), 7.04 (s, 2H), 3.91 (s, 6H), 3.80 (s, 3H). ^13^C NMR (101 MHz, DMSO-*d*_6_) δ 167.05, 164.56, 157.45, 150.81, 150.53, 149.35, 143.35, 142.24, 140.61, 139.64, 138.59, 131.86, 130.56, 127.00, 113.40, 65.33, 61.13. HRMS (ESI), *m*/*z*: calcd. for C_20_H_18_N_3_O_4_S, [M+H]^+^ 396.1013; found: 396.1026.

2-(1*H*-imidazol-4-yl)-8-(3,4,5-trimethoxyphenyl)quinazolin-4(3*H*)-one (**A19**).

White solid. Yield 16.9%. Mp 241.0–242.2 °C. ^1^H NMR (400 MHz, DMSO-*d*_6_) δ 8.14 (dd, *J* = 7.9, 1.4 Hz, 1H), 7.92–7.91 (m, 1H), 7.90 (s, 1H), 7.82 (s, 1H), 7.50 (t, *J* = 7.7 Hz, 1H), 7.09 (s, 2H), 3.86 (s, 6H), 3.76 (s, 3H). ^13^C NMR (101 MHz, DMSO-*d*_6_) δ 166.55, 157.43, 152.69, 151.45, 142.80, 142.17, 142.03, 140.24, 139.87, 138.99, 130.66, 130.53, 127.04, 124.44, 113.50, 65.30, 61.13. HRMS (ESI), *m*/*z*: calcd. for C_20_H_19_N_4_O_4_, [M+H]^+^ 379.1401; found: 379.1409.

#### 3.1.2. General Procedure for the Synthesis of Intermediates **b**

To a solution of 3-bromo-1,2-benzenediamine (100 mg, 0.53 mmol) in 5 mL of dioxane were added aldehyde (0.67 mmol) and TsOH (0.08 mmol). The mixture was stirred at 95 °C under a nitrogen atmosphere for 6 h. Then, the solvent was removed in vacuo, and the residue was diluted by H_2_O and extracted with EtOAc (3 × 10 mL). The combined organic layer was then washed with brine, dried over anhydrous Na_2_SO_4_, and concentrated in vacuo to provide the crude product, which was directly subjected to silica gel column chromatography eluted with petroleum PE/EtOAc (2/1, *v*/*v*) to give corresponding pure intermediates **b**.

4-Bromo-2-(2-(trifluoromethyl)phenyl)-1*H*-benzo[*d*]imidazole (**b0**).

White solid. Yield 37.2%. Mp 178.3–179.5 °C. ^1^H NMR (600 MHz, CDCl_3_) δ 7.92 (d, *J* = 6.8 Hz, 1H), 7.81–7.78 (m, 1H), 7.64–7.58 (m, 3H), 7.50–7.47 (m, 1H), 7.19 (t, *J* = 7.9 Hz, 1H). ^13^C NMR (151 MHz, CDCl_3_) δ 148.44, 131.90, 131.16, 129.25, 127.53, 127.26, 127.06, 125.41, 125.37, 125.05, 123.20.

4-Bromo-2-(4-(trifluoromethyl)phenyl)-1*H*-benzo[*d*]imidazole (**b1**).

White solid. Yield 38.9%. Mp 176.3–177.9 °C. ^1^H NMR (500 MHz, DMSO-*d*_6_) δ 13.51 (s, 1H), 8.53 (s, 2H), 7.88 (d, *J* = 7.8 Hz, 1H), 7.81 (t, *J* = 7.8 Hz, 1H), 7.61 (s, 1H), 7.46 (dd, *J* = 7.7, 0.8 Hz, 1H), 7.19 (t, *J* = 7.9 Hz, 1H). ^13^C NMR (126 MHz, DMSO-*d*_6_) δ 150.88, 131.06, 130.74, 130.42, 130.16, 127.76, 127.12, 127.09, 125.60, 124.68, 123.43, 121.27, 112.50, 111.78.

4-Bromo-2-(2,6-dimethylphenyl)-1*H*-benzo[*d*]imidazole (**b2**).

Yellow solid. Yield 25.6%. Mp 99.7–101.2 °C. ^1^H NMR (500 MHz, DMSO-*d*_6_) δ 12.94 (s, 1H), 7.57 (s, 1H), 7.44 (dd, *J* = 7.8, 0.9 Hz, 1H), 7.34 (t, 1H), 7.21–7.20 (m, 2H), 7.17 (t, *J* = 7.9 Hz, 1H), 2.11 (s, 6H). ^13^C NMR (126 MHz, DMSO-*d*_6_) δ 152.49, 146.86, 146.42, 137.82, 131.65, 129.85, 127.78, 124.89, 124.79, 123.74, 123.70, 20.20.

4-Bromo-2-(2,6-dimethoxyphenyl)-1*H*-benzo[*d*]imidazole (**b3**).

Yellow solid. Yield 60.2%. Mp 112.8–114.8 °C. ^1^H NMR (500 MHz, DMSO-*d*_6_) δ 12.76 (s, 1H), 7.52–7.48 (t, *J* = 8.4 Hz, 2H), 7.41 (dd, *J* = 7.8, 0.8 Hz, 1H), 7.14 (t, *J* = 7.9 Hz, 1H), 6.82 (d, *J* = 8.5 Hz, 2H), 3.72 (s, 6H). ^13^C NMR (126 MHz, DMSO-*d*_6_) δ 159.37, 148.02, 135.55, 132.21, 124.34, 123.51, 121.59, 109.50, 104.57, 99.99, 56.25.

4-Bromo-2-(2,4-dimethoxyphenyl)-1*H*-benzo[*d*]imidazole (**b4**).

Orange solid. Yield 28.1%. Mp 83.6–84.2 °C. ^1^H NMR (500 MHz, DMSO-*d*_6_) δ 12.24 (s, 1H), 8.29 (d, *J* = 8.6 Hz, 1H), 7.60 (d, *J* = 7.9 Hz, 1H), 7.39 (d, *J* = 7.6 Hz, 1H), 7.11 (t, *J* = 7.9 Hz, 1H), 6.78 (d, *J* = 2.3 Hz, 1H), 6.75 (dd, *J* = 8.7, 2.3 Hz, 1H), 4.04 (s, 3H), 3.88 (s, 3H). ^13^C NMR (126 MHz, DMSO-*d*_6_) δ 162.86, 158.75, 150.40, 131.67, 124.68, 123.31, 110.88, 106.84, 99.04, 56.39, 56.01.

4-Bromo-2-(4-(tert-butyl)phenyl)-1*H*-benzo[*d*]imidazole (**b5**).

Orange solid. Yield 41.2%. Mp 216.3–218.1 °C. ^1^H NMR (500 MHz, DMSO-*d*_6_) δ 13.18 (s, 1H), 8.16 (d, *J* = 8.2 Hz, 2H), 7.61–7.58 (m, 2H), 7.57 (d, *J* = 7.9 Hz, 1H), 7.42 (dd, *J* = 7.8, 0.9 Hz, 1H), 7.15 (t, *J* = 7.9 Hz, 1H), 1.34 (s, 9H). ^13^C NMR (126 MHz, DMSO-*d*_6_) δ 153.53, 152.54, 127.32, 127.08, 126.24, 125.08, 123.98, 35.11, 31.43.

4-Bromo-2-(4-methoxyphenyl)-1*H*-benzo[*d*]imidazole **(b6**).

Orange solid. Yield 27.8%. Mp 209.5–210.2 °C. ^1^H NMR (500 MHz, DMSO*-d*_6_) δ 13.08 (s, 1H), 8.18 (d, *J* = 8.2 Hz, 2H), 7.54 (d, *J* = 7.8 Hz, 1H), 7.40 (dd, *J* = 7.8, 0.9 Hz, 1H), 7.15–7.12 (m, 3H), 3.86 (s, 3H). ^13^C NMR (126 MHz, DMSO*-d*_6_) δ 161.41, 152.60, 128.90, 124.93, 123.75, 122.52, 114.87, 55.84.

4-Bromo-2-(4-nitrophenyl)-1*H*-benzo[*d*]imidazole (**b7**).

Yellow solid. Yield 35.3%. Mp 269.9–270.5 °C. ^1^H NMR (500 MHz, DMSO*-d*_6_) δ 13.62 (s, 1H), 8.46 (d, *J* = 8.4 Hz, 2H), 8.41 (d, *J* = 8.9 Hz, 2H), 7.63 (d, *J* = 6.7 Hz, 1H), 7.49 (dd, *J* = 7.7, 0.8 Hz, 1H), 7.22 (t, *J* = 7.9 Hz, 1H). ^13^C NMR (126 MHz, DMSO*-d*_6_) δ 150.18, 148.51, 135.85, 128.26, 125.86, 125.83, 125.08, 125.03, 125.03, 124.73.

4-Bromo-2-(2-tolyl)-1*H*-benzo[*d*]imidazole (**b8**).

Yellow solid. Yield 44.0%. Mp 165.5–166.8 °C. ^1^H NMR (500 MHz, DMSO*-d*_6_) δ 12.97 (s, 1H), 7.75 (dd, *J* = 7.5, 0.9 Hz, 1H), 7.57 (d, *J* = 5.9 Hz, 1H), 7.46–7.43 (m, 2H), 7.42–7.37 (m, 2H), 7.18 (t, *J* = 7.9 Hz, 1H), 2.60 (s, 3H). ^13^C NMR (126 MHz, DMSO*-d*_6_) δ 153.19, 150.14, 142.53, 137.64, 135.58, 131.68, 130.15, 124.82, 124.03, 111.44, 21.26.

4-Bromo-2-(3-methoxyphenyl)-1*H*-benzo[*d*]imidazole (**b9**).

Yellow solid. Yield 44.5%. Mp 125.7–126.8 °C. ^1^H NMR (500 MHz, DMSO*-d*_6_) δ 7.85–7.83 (m, 1H), 7.81–7.80 (m, 1H), 7.60 (dd, *J* = 8.0, 0.8 Hz, 1H), 7.49 (t, *J* = 8.1 Hz, 1H), 7.45 (dd, *J* = 7.8, 0.8 Hz, 1H), 7.19 (t, *J* = 7.9 Hz, 1H), 7.13–7.10 (m, 1H), 3.89 (s, 3H). ^13^C NMR (126 MHz, DMSO*-d*_6_) δ 160.12, 152.26, 130.99, 130.64, 125.46, 124.37, 119.76, 116.91, 112.31, 55.87.

4-(4-Bromo-1*H*-benzo[*d*]imidazol-2-yl)phenol (**b10**).

White solid. Yield 44.5%. Mp 247.7–249.2 °C. ^1^H NMR (400 MHz, DMSO*-d*_6_) δ 12.99 (s, 1H), 10.04 (s, 1H), 8.04 (d, *J* = 8.6 Hz, 2H), 7.50 (d, *J* = 7.6 Hz, 1H), 7.40–7.36 (m, 1H), 7.11 (t, *J* = 7.8 Hz, 1H), 6.95 (d, *J* = 8.7 Hz, 2H). ^13^C NMR (126 MHz, DMSO*-d*_6_) δ 159.98, 153.02, 133.57, 132.75, 132.53, 132.00, 131.92, 129.28, 129.19, 129.03, 124.80, 123.56, 120.96, 116.19.

#### 3.1.3. General Procedures for the Preparation of Compounds **B0**–**B10**

Intermediate **b0**–**b10** (0.26 mmol) was dissolved in a mixed solvent of 4.5 mL dioxane and 0.5 mL H_2_O. To this solution were added (3,4,5-trimethoxyphenyl) boronic acid (67 mg, 0.32 mmol), K_2_CO_3_ (108 mg, 0.78 mmol), and catalytic equivalent of PdCl_2_(PPh_3_)_2_. The mixture was stirred at 80 °C for 4 h under a nitrogen atmosphere to maintain an inert environment. After the reaction, the solvents were evaporated under reduced pressure, and the resulting resides was quenched with H_2_O and extracted with EtOAc (3 × 10 mL). The combined organic layer was then washed with brine, dried over anhydrous Na_2_SO_4_, and concentrated in vacuo to provide the crude product, which was directly subjected to silica gel column chromatography eluted with petroleum PE/EtOAc (3/1, *v*/*v*) to give corresponding pure compounds **B0**–**B10**.

2-(2-(trifluoromethyl)phenyl)-4-(3,4,5-trimethoxyphenyl)-1*H*-benzo[*d*]imidazole (**B0**).

White solid. Yield 78.8%. Mp 178.2–179.5 °C. ^1^H NMR (600 MHz, CDCl_3_) δ 8.04 (s, 1H), 7.83 (t, *J* = 6.9 Hz, 1H), 7.78–7.57 (m, 3H), 7.41–7.39 (m, 2H), 6.95 (s, 2H), 3.91 (s, 6H), 3.86 (s, 3H).^13^C NMR (151 MHz, CDCl_3_) δ 153.77, 149.20, 137.77, 133.82, 132.92, 132.39, 130.18, 126.53 (d, *J* = 5.5 Hz), 123.59, 60.92, 56.0. HRMS (ESI), *m*/*z*: calcd. for C_23_H_20_F_3_N_2_O_3_, [M+H]^+^ 429.1421; found: 429.1418.

2-(3-(Trifluoromethyl)phenyl)-4-(3,4,5-trimethoxyphenyl)-1*H*-benzo[*d*]imidazole (**B1**).

White solid. Yield 79.3%. Mp 181.2–183.1 °C. ^1^H NMR (500 MHz, DMSO-*d*_6_) δ 13.32 (s, 1H), 8.60 (s, 1H), 8.53 (d, *J* = 5.8 Hz, 1H), 7.81–7.86 (m, 2H), 7.65 (s, 2H), 7.56 (d, *J* = 5.0 Hz, 2H), 7.34 (t, *J* = 7.5 Hz, 1H), 3.92 (s, 6H), 3.76 (s, 3H). ^13^C NMR (126 MHz, DMSO-*d*_6_) δ 153.53, 153.16, 149.98, 141.60, 137.53, 136.47, 133.91, 131.59, 130.65, 127.82, 126.68, 125.65, 123.48, 120.93, 120.82, 118.78, 118.71, 118.70, 115.71, 111.22, 106.76, 60.54, 56.27. HRMS (ESI), *m*/*z*: calcd. for C_23_H_20_F_3_N_2_O_3_, [M+H]^+^ 429.1421; found: 429.1436.

2-(2,6-Dimethoxyphenyl)-4-(3,4,5-trimethoxyphenyl)-1*H*-benzo[*d*]imidazole (**B2**).

Brown solid. Yield 57.8%. Mp 153.1–155.7 °C. ^1^H NMR (500 MHz, DMSO-*d*_6_) δ 12.66 (s, 1H), 9.21 (s, 1H), 7.31 (d, *J* = 1.4 Hz, 1H), 7.29 (d, *J* = 1.7 Hz, 1H), 7.19 (d, *J* = 7.1 Hz, 2H), 6.05 (s, 2H), 5.97 (s, 1H), 3.75 (s, 3H), 3.71 (s, 3H), 3.69 (s, 6H), 3.55 (s, 3H). ^13^C NMR (126 MHz, DMSO-*d*_6_) δ 187.55, 176.58, 157.74, 154.30, 153.77, 137.88, 137.42, 130.81, 129.58, 107.54, 93.31, 60.57, 60.51, 56.93, 56.27, 56.01, 20.35. HRMS (ESI), *m*/*z*: calcd. for C_24_H_25_N_2_O_3_, [M+H]^+^ 389.1860; found: 389.1877.

2-(2,6-Dimethylphenyl)-4-(3,4,5-trimethoxyphenyl)-1*H*-benzo[*d*]imidazole (**B3**).

Orange solid. Yield 55.0%. Mp 83.6–84.2 °C. ^1^H NMR (500 MHz, DMSO-*d*_6_) δ 12.49 (s, 1H), 7.64 (d, *J* = 1.6 Hz, 1H), 7.58 (d, *J* = 2.8 Hz, 1H), 7.56 (d, *J* = 1.7 Hz, 1H), 7.47 (t, *J* = 8.4 Hz, 2H), 7.27 (t, *J* = 7.7 Hz, 1H), 6.81 (d, *J* = 8.5 Hz, 2H), 3.86 (s, 6H), 3.73 (s, 3H), 3.72 (s, 6H). ^13^C NMR (126 MHz, DMSO-*d*_6_) δ 159.45, 137.43, 133.63, 132.81, 132.51, 132.49, 132.00, 131.92, 131.84, 129.27, 129.18, 110.26, 104.81, 60.52, 56.30. HRMS (ESI), *m*/*z*: calcd. for C_24_H_25_N_2_O_5_, [M+H]^+^ 421.1758; found: 421.1775.

2-(2,4-Dimethoxyphenyl)-4-(3,4,5-trimethoxyphenyl)-1*H*-benzo[*d*]imidazole (**B4**).

Orange solid. Yield 42.5%. Mp 141.8–143.2 °C. ^1^H NMR (500 MHz, DMSO-*d*_6_) δ 12.08 (s, 1H), 8.33 (d, *J* = 6.4 Hz, 1H), 7.65 (s, 1H), 7.59 (d, *J* = 7.9 Hz, 1H), 7.49 (d, *J* = 7.2 Hz, 1H), 7.26 (t, *J* = 7.8 Hz, 1H), 6.78 (dd, *J* = 5.2, 2.3 Hz, 2H), 6.76 (d, *J* = 2.4 Hz, 1H), 4.05 (s, 3H), 3.92 (s, 6H), 3.87 (s, 3H), 3.76 (s, 3H). ^13^C NMR (126 MHz, DMSO-*d*_6_) δ 162.55, 158.67, 153.14, 149.67, 140.70, 137.29, 134.41, 131.33, 122.38, 111.48, 106.84, 106.66, 99.12, 60.55, 56.34, 55.97. HRMS (ESI), *m*/*z*: calcd. for C_24_H_25_N_2_O_5_, [M+H]^+^ 421.1758; found: 421.1760.

2-(4-(Tert-butyl)phenyl)-4-(3,4,5-trimethoxyphenyl)-1*H*-benzo[*d*]imidazole (**B5**).

Yellow solid. Yield 81.2%. Mp 180.4–181.6 °C. ^1^H NMR (500 MHz, DMSO-*d*_6_) δ 13.00 (s, 1H), 8.18 (d, *J* = 5.3 Hz, 2H), 7.74–7.66 (m, 2H), 7.60 (d, *J* = 7.9 Hz, 2H), 7.53 (s, 2H), 7.30 (t, *J* = 7.7 Hz, 1H), 3.92 (s, 6H), 3.77 (s, 3H), 1.34 (s, 9H). ^13^C NMR (126 MHz, DMSO-*d*_6_) δ 153.16, 153.11, 137.39, 134.17, 127.89, 126.73, 126.27, 106.70, 60.54, 56.33, 35.08, 31.45. HRMS (ESI), *m*/*z*: calcd. for C_26_H_29_N_2_O_3_, [M+H]^+^ 417.2173; found: 417.2192.

2-(4-Methoxyphenyl)-4-(3,4,5-trimethoxyphenyl)-1*H*-benzo[*d*]imidazole (**B6**).

Orange solid. Yield 21.7%. Mp 151.7–152.6 °C. ^1^H NMR (500 MHz, DMSO-*d*_6_) δ 12.93 (s, 1H), 8.20 (d, *J* = 8.7 Hz, 2H), 7.53–7.38 (m, 4H), 7.28 (t, *J* = 7.8 Hz, 1H), 7.15–7.13 (m, 2H), 3.92 (s, 6H), 3.86 (s, 3H), 3.76 (s, 3H). ^13^C NMR (126 MHz, DMSO-*d*_6_) δ 161.24, 154.30, 153.77, 153.19, 137.39, 134.14, 128.73, 122.90, 114.87, 106.66, 93.30, 60.54, 56.34, 56.01, 55.83. HRMS (ESI), *m*/*z*: calcd. for C_23_H_23_N_2_O_4_, [M+H]^+^ 391.1653; found: 391.1673.

2-(4-Nitrophenyl)-4-(3,4,5-trimethoxyphenyl)-1*H*-benzo[*d*]imidazole (**B7**).

Yellow solid. Yield 55.4%. Mp 122.3–124.8 °C. ^1^H NMR (400 MHz, DMSO-*d*_6_) δ 13.46 (s, 1H), 8.49–8.44 (m, 4H), 7.63–7.55 (m, 4H), 7.39 (t, *J* = 7.6 Hz, 1H), 3.93 (s, 6H), 3.77 (s, 3H). ^13^C NMR (126 MHz, DMSO-*d*_6_) δ 153.76, 153.26, 148.34, 144.76, 136.45, 133.80, 124.82, 106.76, 98.36, 93.31, 91.73, 86.08, 85.66, 78.53, 75.09, 60.55, 60.22, 56.41, 56.02. HRMS (ESI), *m*/*z*: calcd. for C_22_H_20_N_3_O_5_, [M+H]^+^ 406.1398; found: 406.1416.

2-(O-tolyl)-4-(3,4,5-trimethoxyphenyl)-1*H*-benzo[*d*]imidazole (**B8**).

Orange solid. Yield 76.6%. Mp 155.7–157.1 °C. ^1^H NMR (500 MHz, DMSO-*d*_6_) δ 12.83 (s, 1H), 7.86 (s, 1H), 7.60–7.53 (m, 4H), 7.41 (s, 3H), 7.33 (t, *J* = 7.7 Hz, 1H), 3.90 (s, 6H), 3.75 (s, 3H), 2.77 (s, 3H). ^13^C NMR (126 MHz, DMSO-*d*_6_) δ 153.15, 152.24, 141.66, 137.67, 137.45, 135.65, 134.25, 132.01, 129.80, 129.57, 126.52, 123.18, 120.54, 110.88, 106.71, 60.54, 56.30, 21.97. HRMS (ESI), *m*/*z*: calcd. for C_23_H_23_N_2_O_3_, [M+H]^+^ 375.1704; found: 375.1722.

2-(3-Methoxyphenyl)-4-(3,4,5-trimethoxyphenyl)-1*H*-benzo[*d*]imidazole (**B9**).

Orange solid. Yield 62.5%. Mp 110.9–112.0 °C. ^1^H NMR (500 MHz, DMSO-*d*_6_) δ 13.06 (s, 1H), 9.22 (s, 1H), 7.85 (s, 1H), 7.66–7.61 (m, 1H), 7.50–7.47 (m, 2H), 7.32 (t, *J* = 7.7 Hz, 1H), 7.08 (dd, *J* = 7.9, 2.0 Hz, 1H), 6.07 (s, 1H), 5.96 (s, 1H), 3.93 (s, 6H), 3.88 (s, 3H), 3.76 (s, 3H). ^13^C NMR (126 MHz, DMSO-*d*_6_) δ 187.52, 176.56, 160.08, 157.71, 154.30, 153.78, 153.19, 137.44, 134.04, 131.85, 130.58, 123.30, 116.44, 107.51, 106.66, 93.31, 60.54, 56.90, 56.30, 56.01, 55.67. HRMS (ESI), *m*/*z*: calcd. for C_23_H_23_N_2_O_4_, [M+H]^+^ 391.1653; found: 391.1663.

4-(4-(3,4,5-Trimethoxyphenyl)-1*H*-benzo[*d*]imidazol-2-yl)phenol (**B10**).

White solid. Yield 42.4%. Mp 293.5–294.7 °C. ^1^H NMR (500 MHz, DMSO-*d*_6_) δ 12.79 (s, 1H), 9.97 (s, 1H), 8.07 (d, *J* = 8.8 Hz, 2H), 7.66–7.61 (m, 2H), 7.58–7.54 (m, 1H), 7.25 (t, *J* = 7.7 Hz, 2H), 6.95–6.93 (m, 2H), 3.91 (s, 6H), 3.76 (s, 3H). ^13^C NMR (126 MHz, DMSO-*d*_6_) δ 159.65, 153.12, 137.32, 134.31, 131.99, 131.92, 129.28, 129.18, 128.63, 122.58, 121.58, 116.15, 106.70, 60.54, 56.35. HRMS (ESI), *m*/*z*: calcd. for C_22_H_21_N_2_O_4_, [M+H]^+^ 377.1496; found: 377.1510.

### 3.2. Biological Evaluation

#### 3.2.1. Bio-Layer Interferometry

The binding affinity of corosolic acid to proteins was detected by the bio-layer interferometry (BLI) assay on an Octet RED96 System (ForteBio, Silicon Valle, CA, USA). Purified porcine brain tubulin was immobilized on ReadTM Streptavidin Biosensors using the biotinylation kit (GENEMORE, Shanghai, China) according to the manufacturer’s protocol. Various concentrations of the corosolic acid were added into the mobile phase, and the association between the immobilized protein and flowing corosolic acid was detected. All running buffers were PBS plus 0.1% DMSO and 0.02% Tween-20. The data were analyzed by ForteBio Data Analysis 9.0 software.

#### 3.2.2. Cytotoxicity Assay

The cytotoxicity of the test compounds on MCF-7, A549, MDA-MB-231, and Hela cells was determined using the CCK-8 (Beyotime, Shanghai, China) method. Cells were seeded at a density of 5000 cells/well in a 96-well plate. After removing the medium, 100 μL of medium containing different concentrations of the compound was added to each well, and the plate was incubated for 48 h at 37 °C with 5% CO_2_. The old medium was aspirated, and CCK-8 reagent diluted with the basic medium was added to each well, followed by an additional 1 h incubation. Absorbance was measured at 450 nm using a multifunctional microplate reader. The IC_50_ was calculated through nonlinear regression analysis using GraphPad Prism (8.0.2). All experiments were repeated at least three times.

#### 3.2.3. In Vitro Tubulin Polymerization Assay

Pig brain microtubule protein (Cytoskeleton, Denver, CO, USA) was solubilized in EM buffer supplemented with 5% glycerol (80 mM PIPES, 2 mM GTP, 2 mM MgCl_2_, and 0.5 mM EGTA, pH 6.9). Subsequently, the test compounds were formulated in the same EM buffer to generate a concentration gradient of 50, 25, 10, 5, and 2.5 μM, aliquoted into a 384-well plate, and pre-incubated at 37 °C. Finally, the test compound solutions were added to the tubulin mixture, and alterations in fluorescence intensity were tracked using a multifunction microplate reader at an excitation wavelength of 355 nm and an emission wavelength of 420 nm, with readings recorded every 60 s over a 60 min period.

#### 3.2.4. Immunofluorescence Staining

MCF-7 cells were plated onto glass-bottomed dishes and subsequently exposed to 0.1% DMSO as the vehicle control or compound **B6** at concentrations of 0.1, 1, and 10 μM for a 24 h treatment period. The cells were immobilized using 4% paraformaldehyde solution and then permeabilized in PBS supplemented with 0.2% Triton X-100 (Beyotime, Shanghai, China). After undergoing blocking at room temperature for 30 min with 100 μL of goat serum albumin, the cells were incubated with an anti-β-tubulin primary antibody (Cell Signaling Technology, Danvers, MA, USA) at 37 °C for 1 h. Next, the cells were rinsed three times with PBS and stained with a fluorescent secondary antibody. Cell nuclei were counterstained with 4,6-diamidino-2-phenylindole (DAPI). Fluorescent signals in the cells were ultimately observed and imaged under a fluorescence microscope.

#### 3.2.5. Colony Formation Assay

MCF-7 cells (1000 cells per well) were plated in 6-well culture plates. Following a 24 h incubation at 37 °C, the culture medium was replaced with fresh medium supplemented with compound **B6** at prespecified concentrations. After 24 h of drug exposure, the medium was switched back to regular culture medium. The cells were further incubated for an additional 10–14 days, after which the formed colonies were immobilized with 4% paraformaldehyde for 30 min and dyed with 0.1% crystal violet for 15 min. Subsequent to rinsing with PBS, the colonies were imaged for further analysis.

#### 3.2.6. Transwell Assay

MCF-7 cells were seeded at a density of 5 × 10^5^ cells/well in a 6-well plate and cultured at 37 °C for 24 h. Different concentrations of compound **B6** (0.1, 1, and 10 μM) were then added for 24 h of treatment. The cells were digested to create a single-cell suspension. The serum-free cell suspension was added to the upper chamber of a Transwell insert, while the lower chamber contained normal culture medium with serum as a chemotactic inducer. The setup was incubated for 48 h. The medium in the upper chamber was removed, and the chamber was gently washed three times with pre-cooled PBS along the walls. Cells were fixed with pre-cooled methanol for 15 min and stained with crystal violet solution, protected from light for 30 min. Non-migrated cells in the upper chamber were wiped off with a sterile damp cotton swab. The number of cells that migrated through the membrane was observed and counted under an inverted fluorescence microscope. Finally, the number of migrated cells in the experimental group was compared with the control group.

#### 3.2.7. Wound Healing Assay

MCF-7 cells were seeded and incubated overnight in a 6-well plate. A scratch was made on the confluent monolayer using a 200 μL pipette tip. The wound was then washed twice with medium to remove debris and dislodged cells. Different concentrations of compound **B6** (0, 0.1, 1, and 10 μM) were added. Images were captured at 0 h and 24 h using phase contrast microscopy, and the migration distance of cells in the wound area was measured manually.

#### 3.2.8. Analysis of Cell Cycle

MCF-7 cells were seeded at a density of 5 × 10^5^ cells/well in a 6-well plate and cultured overnight. Cells were treated with compound **B6** (0.1, 1, and 10 μM) for 48 h. The cells were collected by centrifugation for 5 min and then fixed overnight at 4 °C with pre-cooled 70% ethanol (freshly prepared using PBS buffer). After fixation, the cell pellet was washed once with pre-cooled PBS, and then 0.5 mL of propidium iodide (PI) staining solution (Beyotime, China) was added to each tube of cell samples. The cell pellet was slowly and thoroughly resuspended using a vortex mixer and stained at 37 °C in the dark for 30 min. Subsequently, red fluorescence signals were detected using a flow cytometer at an excitation wavelength of 488 nm while also collecting light scattering data.

#### 3.2.9. Analysis of Cancer Cell Apoptosis

MCF-7 cells were seeded at a density of 5 × 10^5^ cells per well in a 6-well plate and cultured overnight. The cells were treated with compound **B6** at concentrations of 0.1, 1, and 10 μM for 48 h. After treatment, adherent cells were digested and centrifuged to collect the cell pellet, which was washed once with PBS. Annexin-FITC binding solution was gently added to each sample tube to resuspend the cells. Finally, Annexin-FITC (Beyotime, China) and propidium iodide (PI) staining solutions (Beyotime, China) were added to the sample tubes for dual staining incubation in the dark at room temperature for 10–20 min and analyzed with a flow cytometer.

#### 3.2.10. Molecular Docking

The molecular docking was conducted using MOE (Molecular Operating Environment) 2022.02. The crystal structure of tubulin (PDB ID: 1SA0) was downloaded from the Protein Data Bank (www.rcsb.org) and processed using the Preparation module to simplify the computational model (including residue repair and removal of water molecules). During the docking process, 30 conformations were generated, which were then scored and ranked. The docking scores were checked to select the conformation with the best binding energy, and a visual analysis of the ligand’s binding mode at the binding site was performed to observe key interactions, such as hydrogen bonds and hydrophobic interactions.

#### 3.2.11. Molecular Dynamics Studies

Molecular Dynamics (MD) Simulations. The molecular dynamics simulations were performed on the systems of the tubulin–B6 complex (PDB: 1SA0). The system was solvated in an explicit water box (TIP3P) and neutralized with appropriate counterions using System Builder [33] utilities. Energy minimization and equilibration under NPT were performed. Unrestrained MD production runs of 100 ns per system were conducted at 300 K temperature and 1.01325 bar pressure using the Desmond [33] utilities package. Coordinates were saved every 100 ps interval for analysis. Conformational dynamics were analyzed over whole trajectory by Maestro [34].

#### 3.2.12. In Vivo Antitumor Evaluation

All animal experiments were performed following the protocols evaluated and approved by the Institutional Animal Care and Use Committee (IACUC) of Second Military Medical University (Ethics Approval Number: 82204347). Male BALB/c mice, 6−8 weeks old, purchased from Ziyuan (Zhejiang, China) Biotechnology Co., Ltd., were used to study the inhibition effect of compound **B6** on the subcutaneous transplanted model of melanoma cells. Mice were housed in a controlled environment (temperature: 22 ± 2 °C, humidity: 50 ± 10%) with a 12 h light/dark cycle and provided with standard laboratory diet and water ad libitum, along with environmental enrichment such as nesting material and shelters.

Logarithmic growth phase B16-F10 cells (4 × 10^6^ per mL) were suspended in PBS before injecting into mice. Tumors were established by inoculating subcutaneously with 200 μL containing 5 × 10^5^ cells into each mouse. Mice were randomly divided into control or treatment groups (*n* = 8). Compound **B6** or paclitaxel was dissolved in sterile saline (with 5% DMSO and 35% PEG-400) solution to produce the desired concentrations. The vehicle control formulation was prepared using the identical composition of sterile saline supplemented with 5% DMSO and 35% PEG-400, with no additional test compounds. Drugs at designated doses (paclitaxel at 10 mg/kg; compound **B6** at 20 and 50 mg/kg) or vehicle control were delivered via i.p. injection once every 48 h over a 12-day treatment period.

To evaluate acute toxic effects, the body weights of the experimental mice were tracked continuously throughout the entire study. Tumor volume was determined with a caliper and calculated using the formula a × b^2^ × 0.5, in which a denotes the longer tumor diameter and b signifies the shorter one. At the end of the 12-day treatment period, all mice were sacrificed, and tumors were excised and weighed for TGI calculation. The TGI percentage was calculated as TGI (%) = [1 − Wt/Wv] × 100%, where Wt is the mean tumor weight of treatment groups and Wv is that of the vehicle control group. Livers and kidneys collected from the mice were fixed in 4% paraformaldehyde, processed into paraffin sections, stained with H&E, and observed and photographed under a microscope.

## 4. Conclusions

In summary, we employed a molecular hybridization strategy to successfully design and synthesize 31 new quinazolinone and thienopyridine analogs as tubulin inhibitors. BLI results indicate that within the concentration range of 10^−4^ to 10^−7^ mol·L^−1^, compounds **A4**, **A5**, **A15**, **B3**, **B4**, **B5**, and **B6** demonstrated superior binding potency to tubulin compared to colchicine. The antiproliferative activities against the MCF-7, MDA-MB-231, A549, and HeLa cell lines indicate that compound **B6** was the most promising candidate and displayed strong broad-spectrum anticancer activity with an average IC_50_ value of 2 μM. In vitro microtubule polymerization tests showed that at a concentration of 50 μM, compound **B6** significantly inhibited in vitro tubulin polymerization. Mechanistic investigations demonstrated that compound **B6** suppressed tubulin polymerization, blocked the cell cycle at the G2/M phase, and triggered apoptotic cell death in MCF-7 cells. Molecular docking results showed that the imidazole ring of **B6** formed a key hydrogen bond with tubulin βLys2549, and the rest of the structure was well encapsulated in the hydrophobic pocket formed by tubulin residues, indicating high affinity. This mode of action may be the reason for **B6**’s high antitumor activity. Furthermore, compound **B6** exhibited remarkable in vivo anti-tumor activity in a melanoma tumor model, achieving a notable tumor growth inhibition (TGI) rate of 70.21% at a dosage of 50 mg/kg and showing no obvious toxic effects. Taken together, the present study demonstrates that compound **B6** represents a promising lead molecule worthy of further exploration as a potential anticancer therapeutic.

## Figures and Tables

**Table 1:** Binding affinity values of test compounds for tubulin protein (K_D_ values [μM] were measured via the biolayer interferometry assay).

Compounds	K_D_ (μM)	Compounds	K_D_ (μM)
Colchicine	3.30 ± 1.20	**A15**	1.90 ± 0.60
**A0**	10.6 ± 1.80	**A16**	ND
**A1**	7.50 ± 2.00	**A17**	8.90 ± 1.00
**A2**	22.00 ± 3.60	**A18**	3.30 ± 0.70
**A3**	16.00 ± 1.80	**A19**	9.20 ± 1.60
**A4**	1.40 ± 1.00	**B0**	4.20 ± 1.02
**A5**	0.72 ± 0.80	**B1**	12.00 ± 1.20
**A6**	4.90 ± 1.70	**B2**	NB
**A7**	56.00 ± 3.80	**B3**	0.58 ± 0.50
**A8**	17.00 ± 2.40	**B4**	2.20 ± 1.10
**A9**	3.70 ± 2.70	**B5**	2.90 ± 1.00
**A10**	NB	**B6**	0.27 ± 0.30
**A11**	79.00 ± 2.30	**B7**	14.00 ± 1.60
**A12**	ND	**B8**	7.50 ± 0.80
**A13**	22.00 ± 1.10	**B9**	9.70 ± 1.20
**A14**	13.00 ± 1.90	**B10**	3.50 ± 0.90

ND = not detected; NB = no binding or very weak binding.

**Table 2:** In vitro antiproliferative activities of target compounds against four cancer cell lines ^a^.

Compounds	Structure of Fragment III	MCF-7	MDA-MB-231	A549	Hela
		IC_50_/μM	IC_50_/μM	IC_50_/μM	IC_50_/μM
**B1**	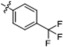	3.80 ± 1.40	12.10 ± 1.50	2.40 ± 1.10	3.60 ± 0.80
**B4**	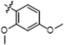	7.60 ± 1.10	23.20 ± 1.10	4.80 ± 1.00	5.10 ± 1.20
**B5**	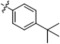	6.60 ± 1.20	17.20 ± 1.20	9.60 ± 1.50	4.10 ± 1.40
**B6**	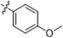	1.40 ± 0.90	2.50 ± 0.70	1.80 ± 0.60	1.80 ± 1.10
**B8**	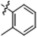	4.20 ± 0.90	12.60 ± 1.20	5.80 ± 1.10	4.40 ± 0.90
**B9**	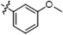	3.60 ± 1.20	7.80 ± 0.90	5.50 ± 1.20	3.60 ± 0.90
**B10**	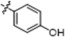	32.30 ± 1.70	41.90 ± 1.70	17.30 ± 1.40	24.90 ± 1.50
Colchicine	/	0.023 ± 0.0090	0.022 ± 0.0060	0.080 ± 0.010	0.004 ± 0.0040

^a^ Cells were treated with varying concentrations of the test compounds for a continuous 48 h period prior to the assessment of cell viability using the CCK-8 assay. IC_50_ values are expressed as the mean ± SD (standard deviation) from a minimum of three independent experimental runs.

## Data Availability

The original contributions presented in this study are included in the article/Supplementary Material. Further inquiries can be directed to the corresponding authors.

## Supplementary Materials

The following supporting information can be downloaded at: https://www.mdpi.com/article/10.3390/ph19010161/s1, Figures S1–S148: Analytical data for the synthesized compounds; In vitro antiproliferative activities of target compounds against B16-F10 cells and HUVEC cells.
