# Supplementary material for: Discovery of New Quinazolinone and Benzimidazole Analogs as Tubulin Polymerization Inhibitors with Potent Anticancer Activities
Source: Pharmaceuticals (Basel). 2026 Jan 15;19(1):161. doi: 10.3390/ph19010161 (PMC12844652; doi:10.3390/ph19010161)
Supplement: Supplementary file 1 [file pharmaceuticals-19-00161-s001.zip › pharmaceuticals-3999374-supplementary.pdf]

Supporting Information

Discovery of New Quinazolinone and Benzimidazole Analogues as Tubulin Polymerization Inhibitors with Potent Anticancer Activities

Boye Jiang [1,3,†], Juan Zhang [2,†], Kai Shao [1], Conghao Gai [1], Bing Xu [1], Yan Zou [1], Yan Song [2], Zhao Qingjie [1], Qingguo Meng [3,*] and Xiaoyun Chai [1,*]

[1] Department of Organic Chemistry, School of Pharmacy, Second Military Medical University, Shanghai 200433, China

[2] PLA Naval Medical Center, Second Military Medical University, Shanghai 200052, China

[3] School of Pharmacy, Yantai University, Yantai 264005, China

[*] Correspondence: qinggmeng@163.com (Q.M.); chaixy1207@163.com (X.C.)

[†] These authors contributed equally to this work.

Table of Content

In vitro antiproliferative activities of target compounds against B16-F10 cells-1

In vitro antiproliferative activities of compound **B6** against HUVEC cells------2

^{1}H NMR, ^{13}C NMR, HRMS and HPLC spectra analysis of the synthesized compounds---3

In vitro antiproliferative activities of target compounds against B16-F10 cells

Compd.	Structure of Fragment III	B16-F10 IC_{50} /μM
**B1**	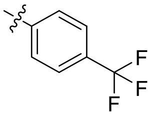	0.90 ± 1.30
**B4**	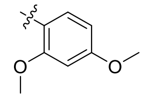	0.80 ± 1.10
**B5**	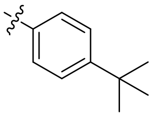	0.70 ± 0.70
**B6**	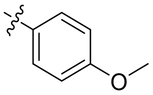	0.40 ± 0.80
**B8**	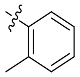	3.10 ± 1.30
**B9**	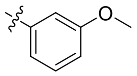	2.10 ± 1.30
**B10**	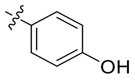	10.00 ± 1.80
Colchicine	/	0.034 ± 0.0050

In vitro antiproliferative activities of compound **B6** against HUVEC cells

Compd.	Structure of Fragment III	HUVEC IC_{50} /μM
**B6**	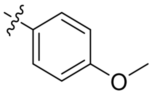	15.6 ± 1.20

Experimental characterization data for products:

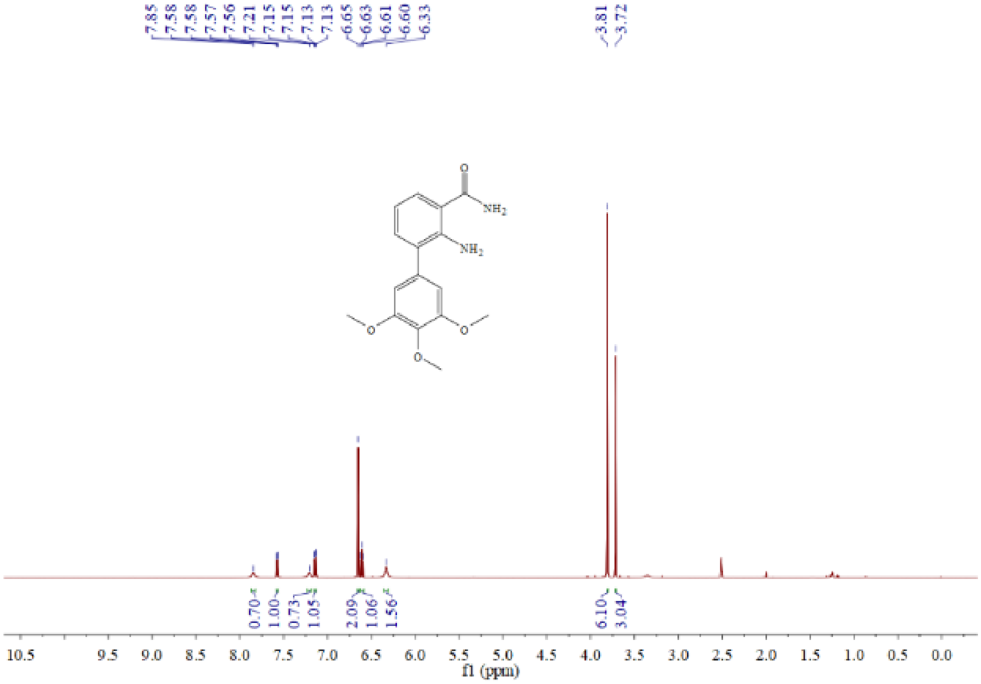

Figure S1: ^{1}H NMR spectrum of 2.

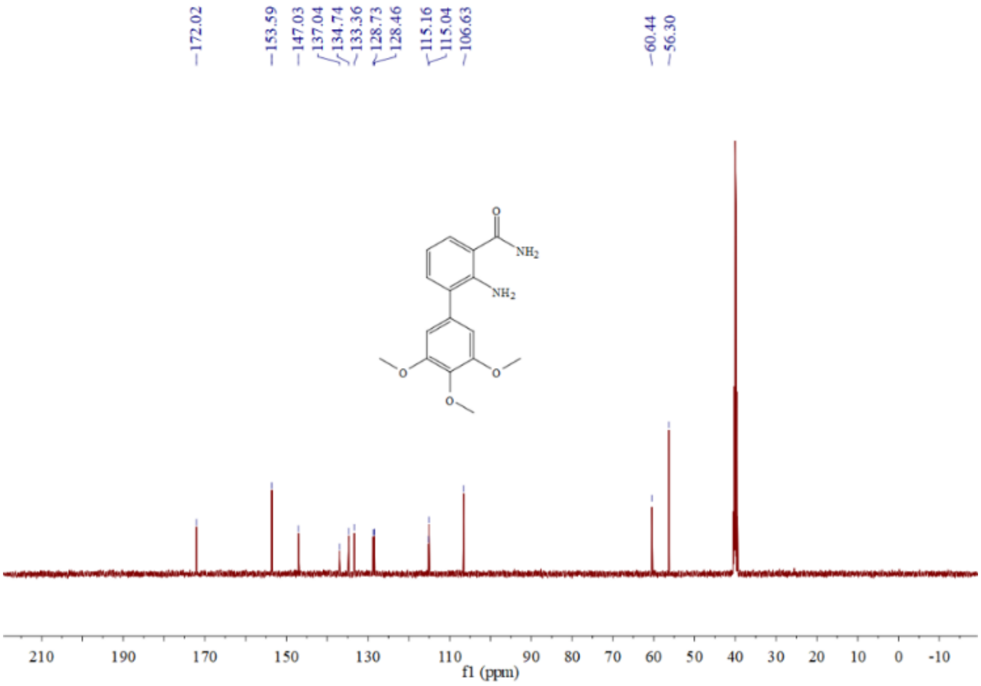

Figure S2: ^{13}C NMR spectrum of 2.

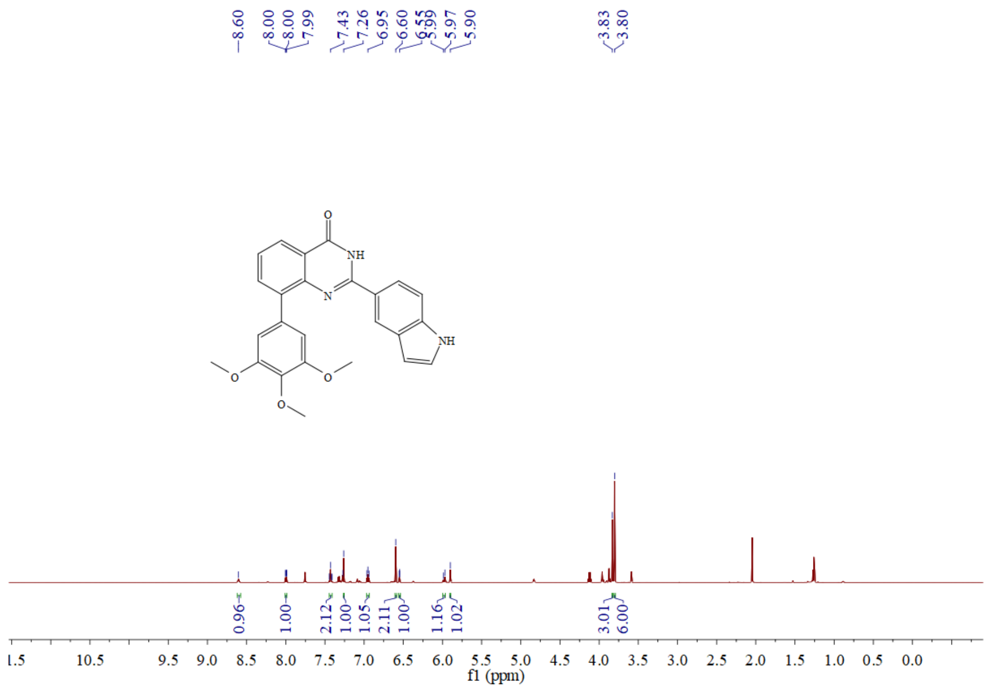

Figure S3: ^{1}H NMR spectrum of A0.

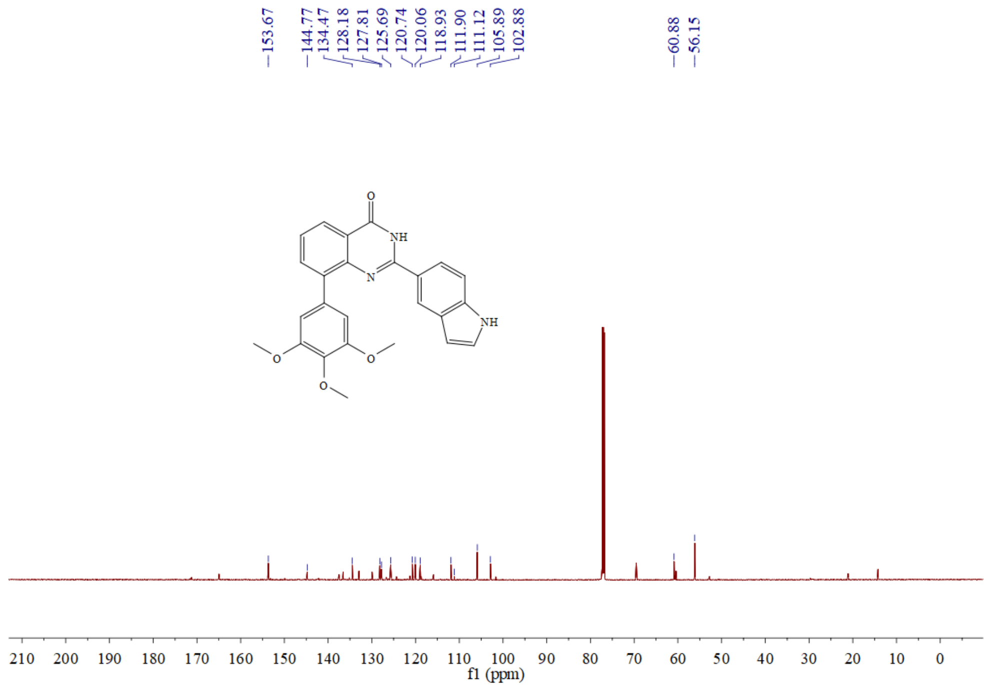

Figure S4: ^{13}C NMR spectrum of A0.

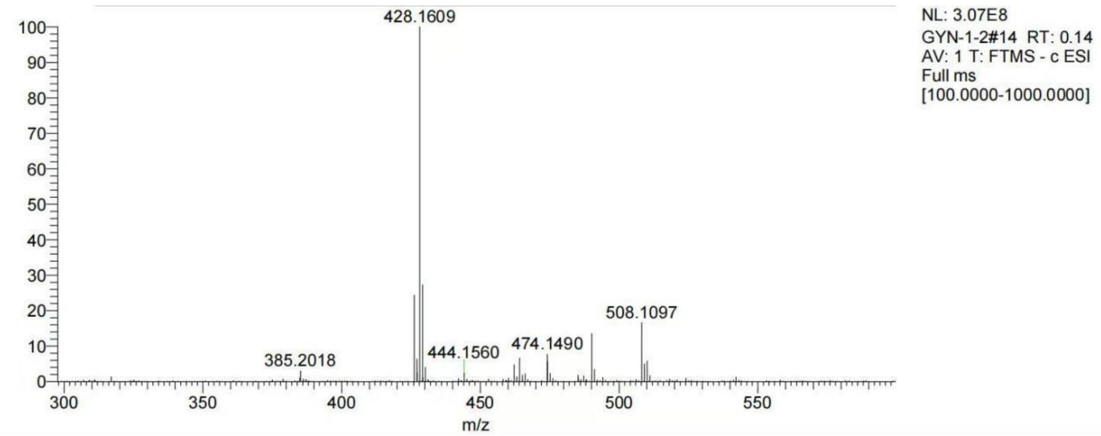

Figure S5: HR MS spectrum of A0.

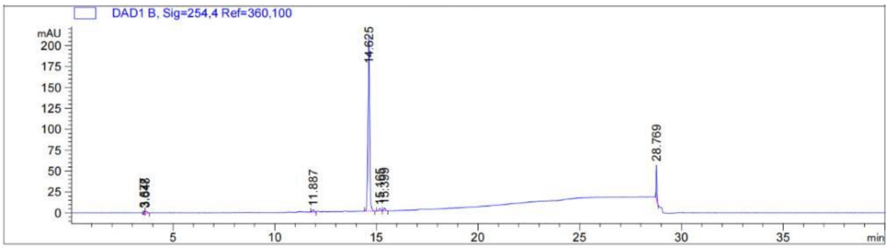

Signal 2: DAD1 B, Sig=254,4 Ref=360,100

Peak #	Retention time [min]	Type	Peak Width [min]	Peak area [mAU*s]	Peak height [mAU]	Peak area %
1	3.577	BV	0.0554	12.86306	3.62314	0.9030
2	3.648	VB	0.1027	22.06337	3.03805	1.5489
3	11.887	BV	0.0979	16.87668	2.66343	1.1848
4	14.625	BB	0.0896	1218.31396	210.11617	85.5307
5	15.165	BV	0.0850	18.09220	3.15140	1.2701
6	15.399	VB	0.0881	20.43691	3.50198	1.4348
7	28.769	BB	0.0440	115.77110	42.16410	8.1276
Total amount :				1424.41729	268.25825	

Figure S6: HPLC spectrum of A0.

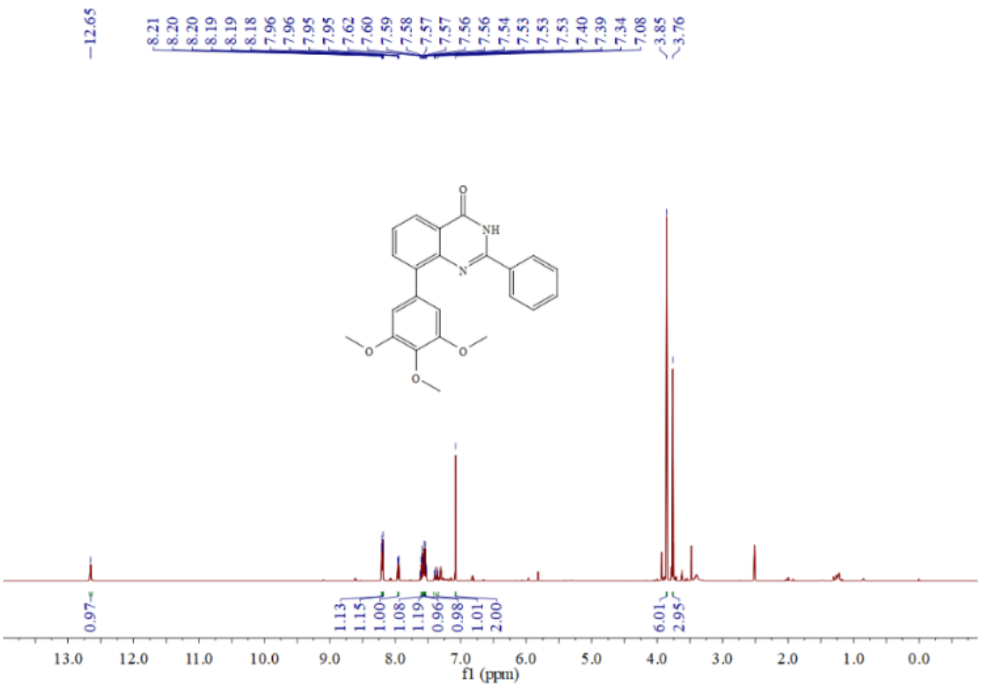

Figure S7: ^{1}H NMR spectrum of A1.

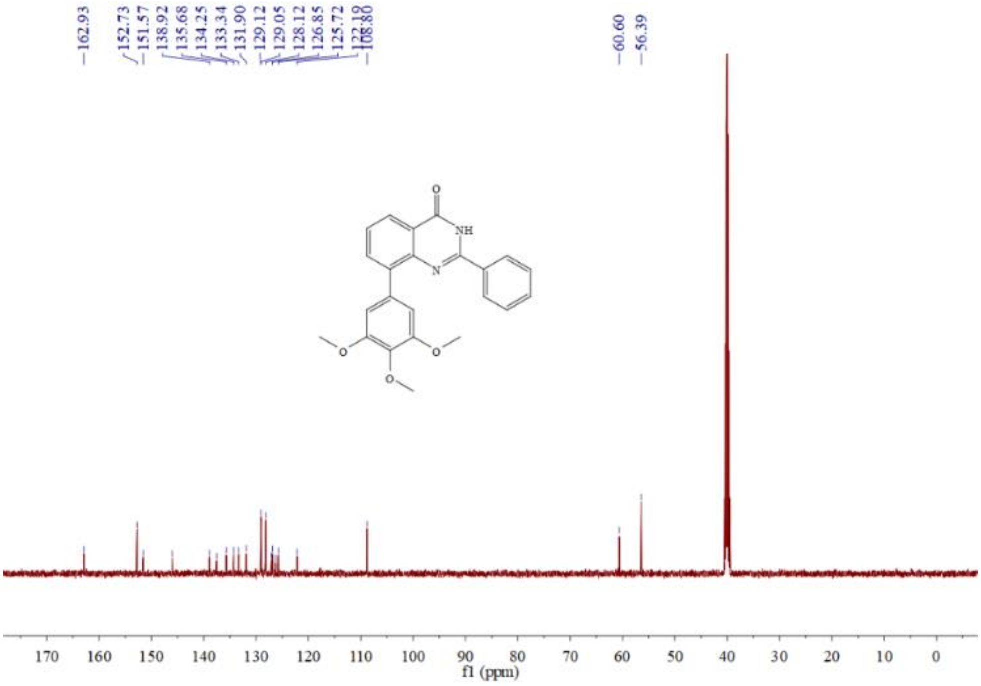

Figure S8: ^{13}C NMR spectrum of A1.

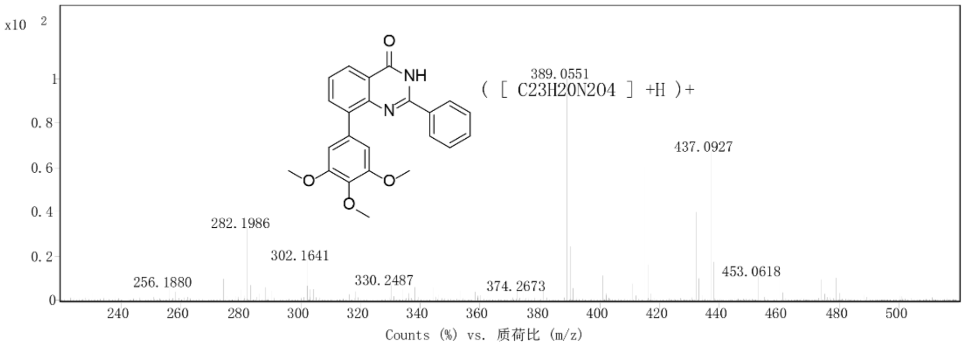

Figure S9: HR MS spectrum of A1.

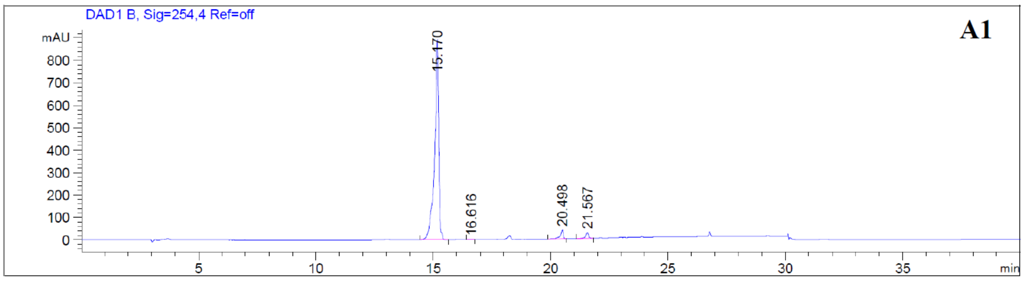

Signal 2: DAD1 B, Sig=254,4 Ref=off

Peak #	Retention time [min]	Type	Peak Width [min]	Peak area [mAU*s]	Peak height [mAU]	Peak area %
1	15.170	BB	0.1623	1.05655e4	891.14899	95.0356
2	16.616	BB	0.1163	11.82137	1.49365	0.1063
3	20.498	BB	0.1189	329.51501	38.87626	2.9640
4	21.567	BB	0.1139	210.57343	26.16139	1.8941

Total amount: 1.11174e4 957.68029

Figure S10: HPLC spectrum of A1.

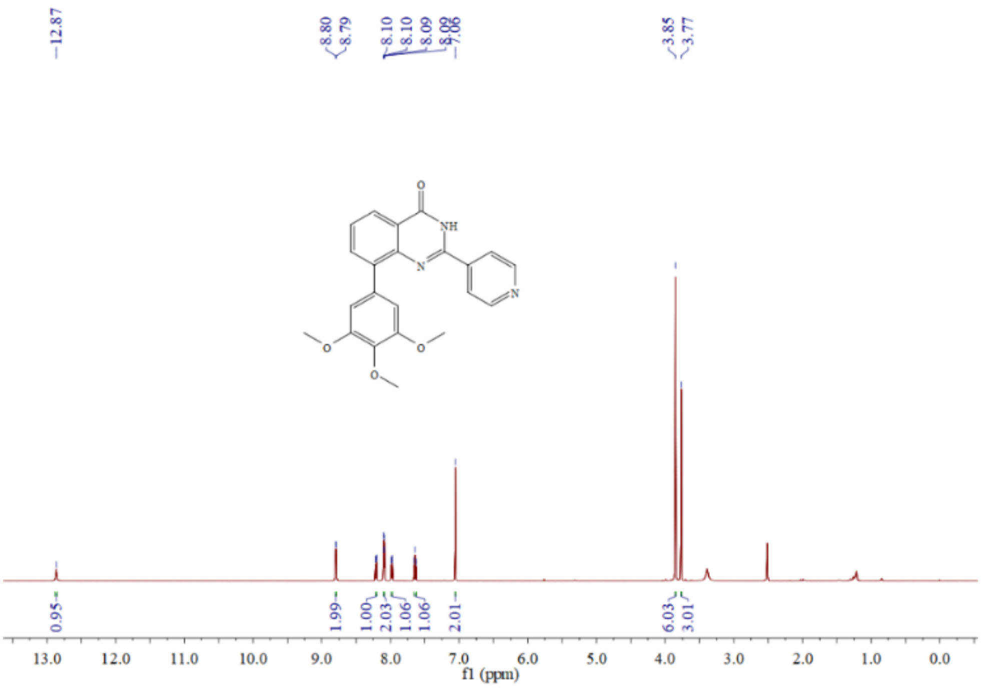

Figure S11: ^{1}H NMR spectrum of A2.

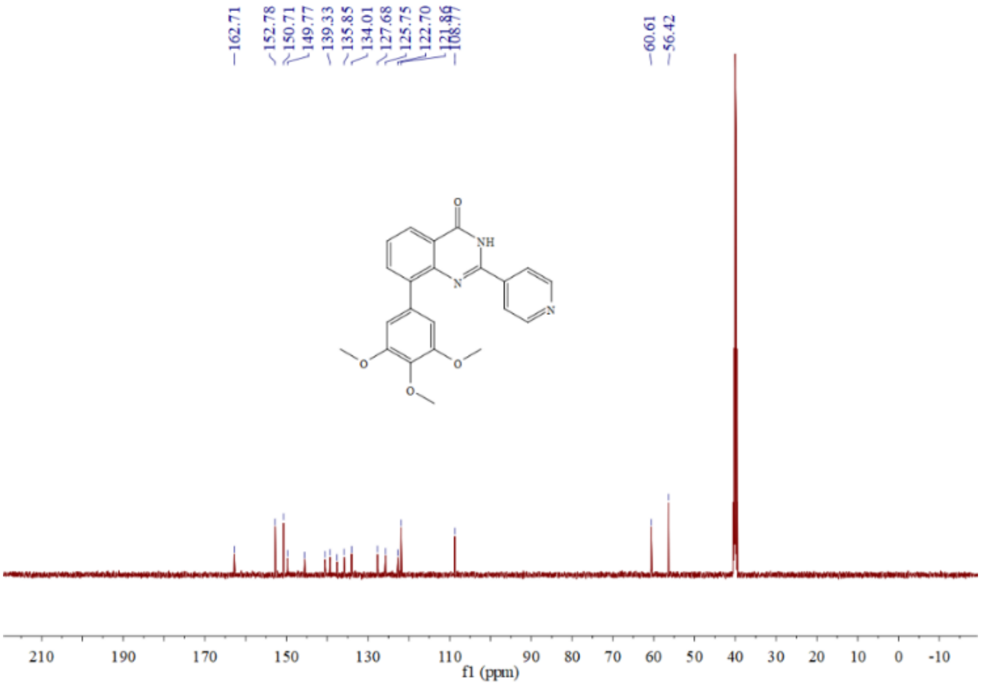

Figure S12: ^{13}C NMR spectrum of A2.

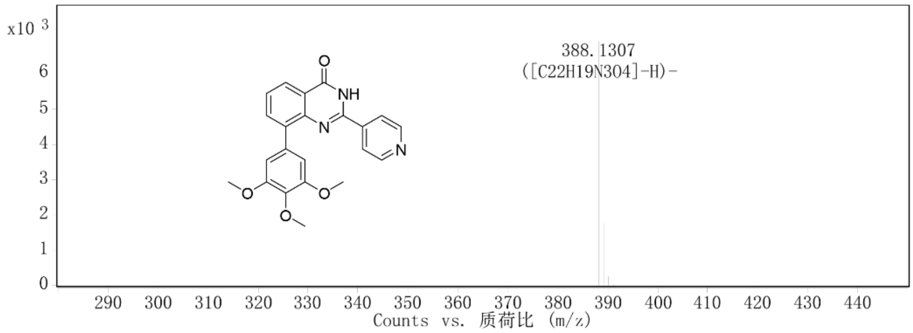

Figure S13: HR MS spectrum of A2.

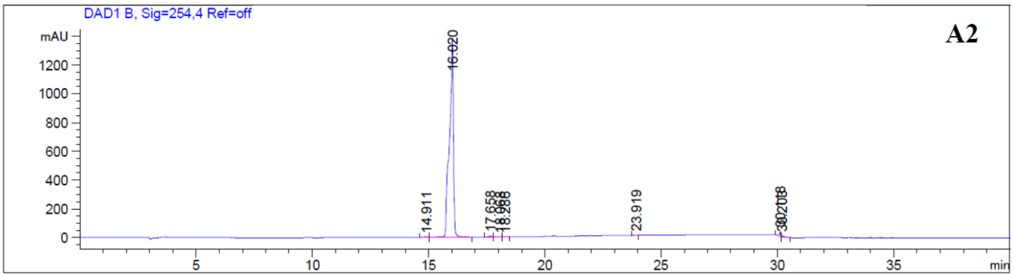

Signal 2: DAD1 B, Sig=254,4 Ref=off

Peak #	Retention time [min]	Type	Peak Width [min]	Peak area [mAU*s]	Peak height [mAU]	Peak area %
1	14.911	BB	0.1450	47.92796	4.47278	0.2859
2	16.020	BB	0.1605	1.63567e4	1378.21045	97.5752
3	17.658	BV	0.1062	54.33441	7.52447	0.3241
4	18.068	VV	0.1568	26.59374	2.20389	0.1586
5	18.286	VB	0.1086	17.85921	2.52136	0.1065
6	23.919	BB	0.0853	11.43639	1.98204	0.0682
7	30.118	BB	0.0620	164.02446	38.30077	0.9785
8	30.203	BB	0.1322	84.29947	9.43841	0.5029
Total amount:				1.67632e4	1444.65416	

Figure S14: HPLC spectrum of A2.

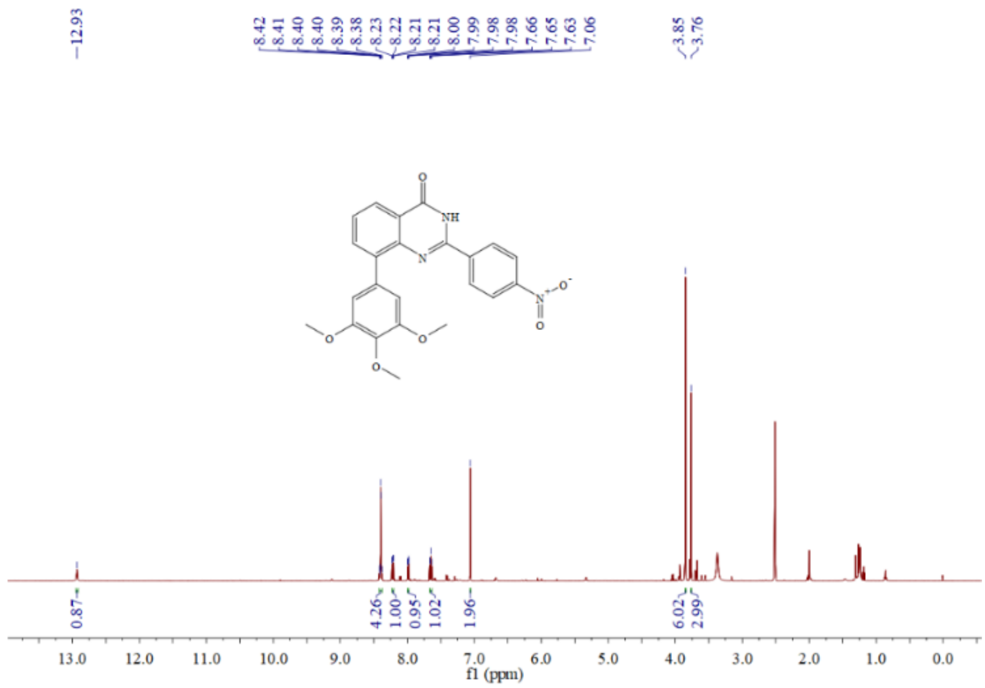

Figure S15: ¹H NMR spectrum of A3.

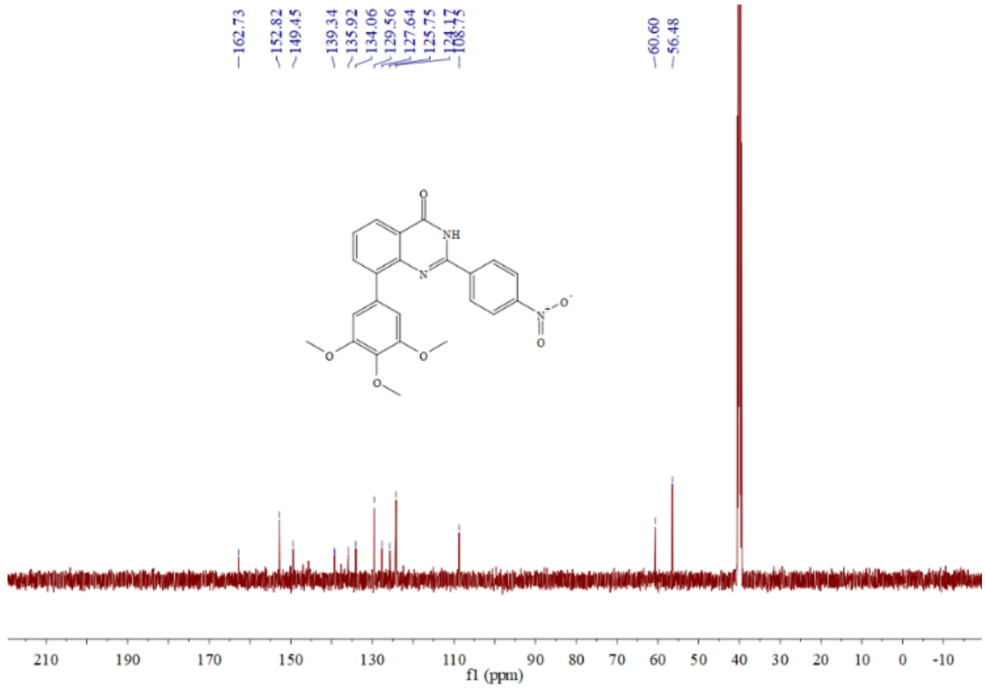

Figure S16: ¹³C NMR spectrum of A3.

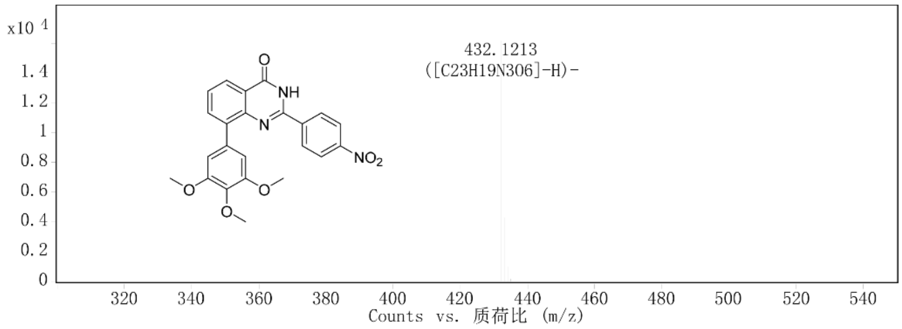

Figure S17: HR MS spectrum of A3.

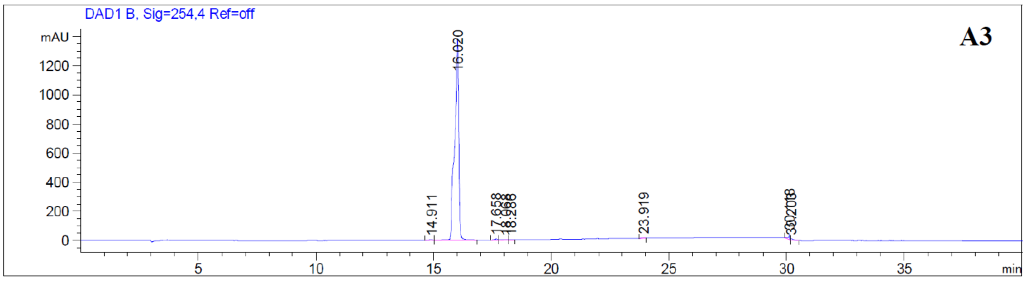

Signal 2: DAD1 B, Sig=254,4 Ref=off

Peak #	Retention time [min]	Type	Peak Width [min]	Peak area [mAU*s]	Peak height [mAU]	Peak area %
1	14.911	BB	0.1450	47.92796	4.47278	0.2859
2	16.020	BB	0.1605	1.63567e4	1378.21045	97.5752
3	17.658	BV	0.1062	54.33441	7.52447	0.3241
4	18.068	VV	0.1568	26.59374	2.20389	0.1586
5	18.286	VB	0.1086	17.85921	2.52136	0.1065
6	23.919	BB	0.0853	11.43639	1.98204	0.0682
7	30.118	BB	0.0620	164.02446	38.30077	0.9785
8	30.203	BB	0.1322	84.29947	9.43841	0.5029
Total amount:				1.67632e4	1444.65416	

Figure S18: HPLC spectrum of A3.

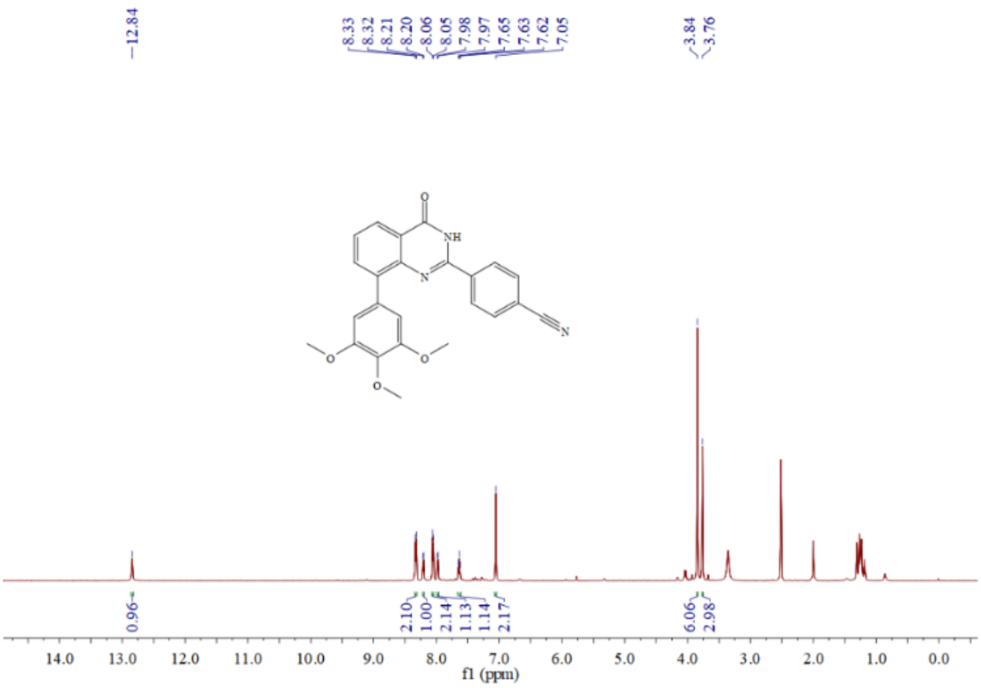

Figure S19: ^{1}H NMR spectrum of A4.

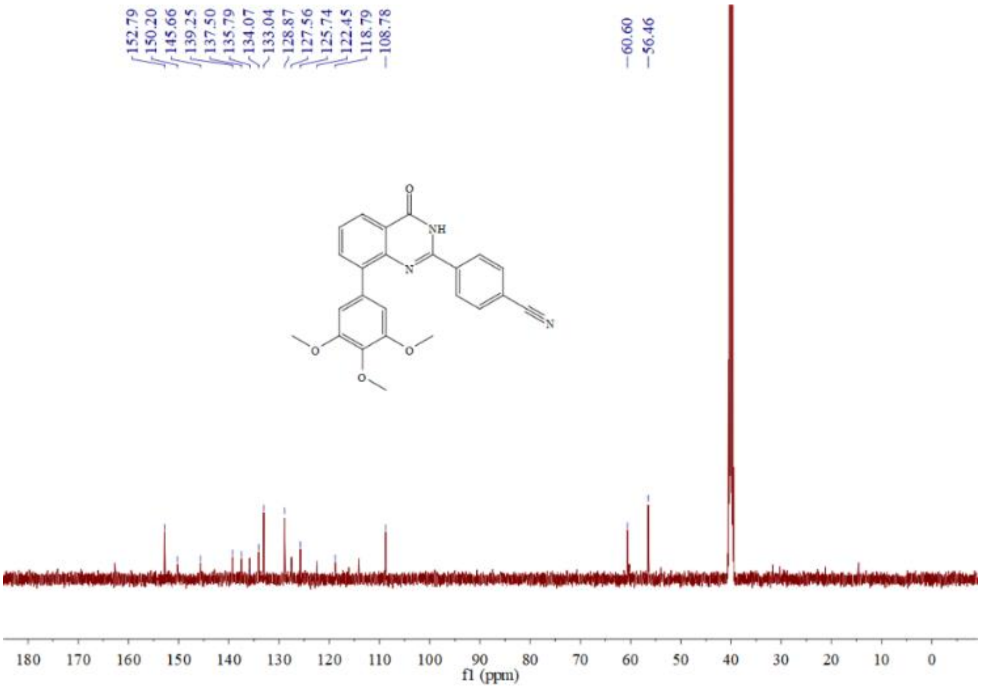

Figure S20: ^{13}C NMR spectrum of A4.

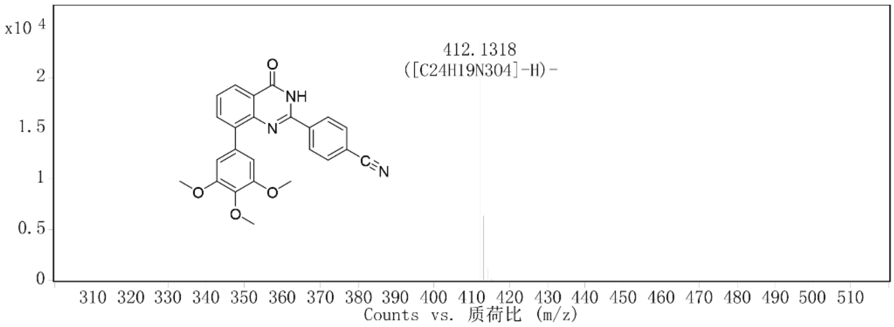

Figure S21: HR MS spectrum of A4.

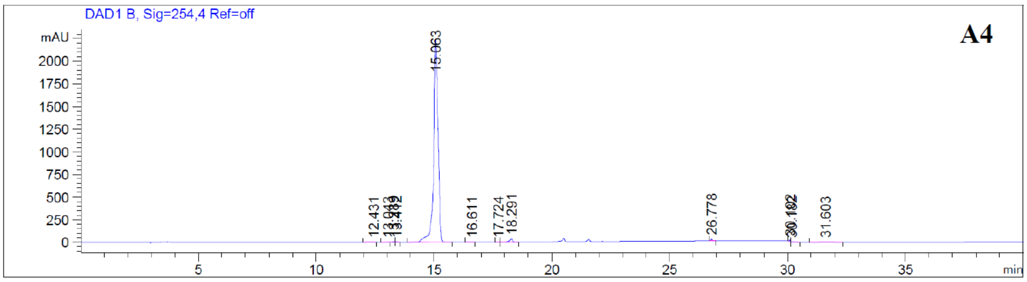

Signal 2: DAD1 B, Sig=254,4 Ref=off

Peak #	Retention time [min]	Type	Peak Width [min]	Peak area [mAU*s]	Peak height [mAU]	Peak area %
1	12.431	BB	0.1761	51.65749	3.76430	0.1752
2	13.043	BV E	0.1537	12.08028	1.05430	0.0410
3	13.289	VV R	0.1232	76.45148	8.81511	0.2592
4	13.412	VB	0.0843	53.89606	9.48398	0.1828
5	15.063	VB R	0.1875	2.86498e4	2236.23657	97.1525
6	16.611	BB	0.1477	14.96601	1.43421	0.0508
7	17.724	BB	0.0929	7.19364	1.18355	0.0244
8	18.291	BB	0.1287	307.23431	32.96414	1.0418
9	26.778	BB	0.0788	105.43825	20.91617	0.3575
10	30.102	BB	0.0455	56.16903	19.57383	0.1905
11	30.182	BB	0.1326	75.40273	8.41111	0.2557
12	31.603	BB	0.4075	79.20998	2.31335	0.2686
Total amount:				2.94895e4	2346.15062	

Figure S22: HPLC spectrum of A4.

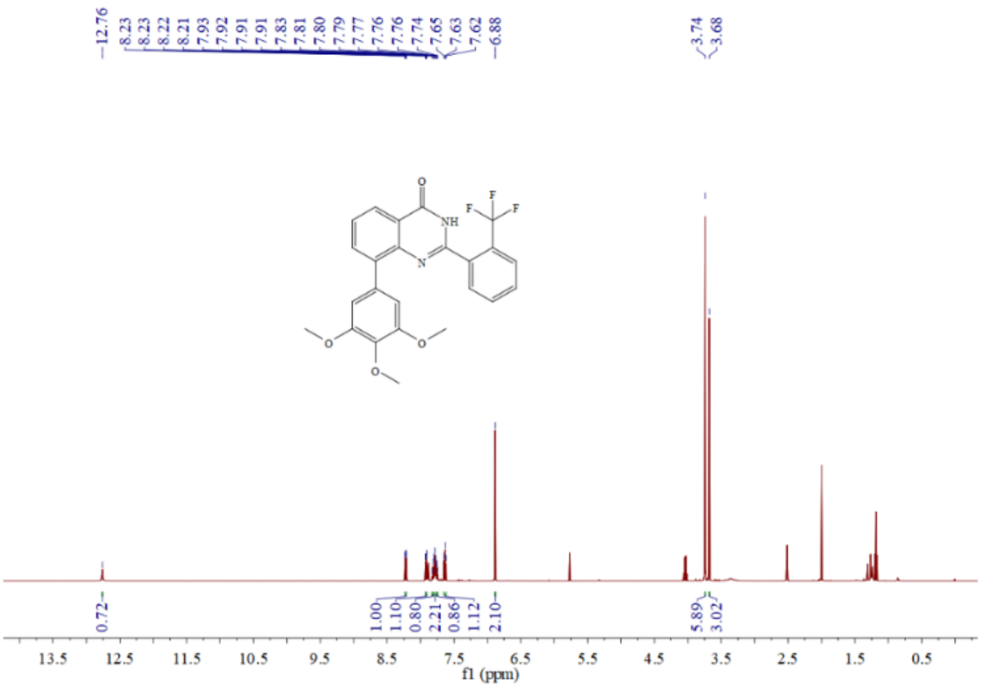

Figure S23: ^{1}H NMR spectrum of A5.

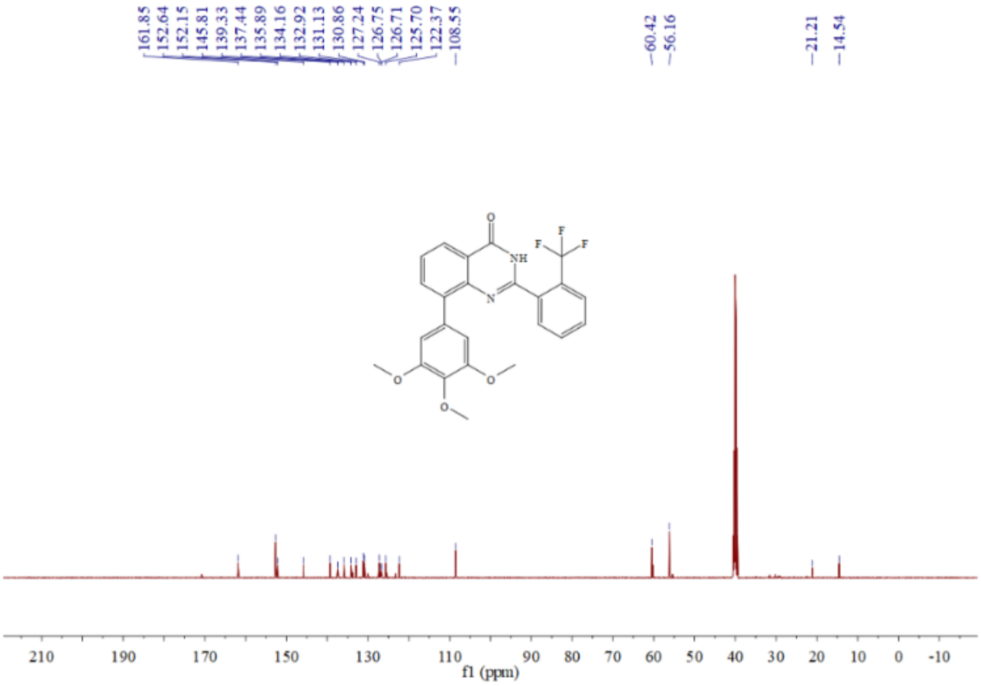

Figure S24: ^{13}C NMR spectrum of A5.

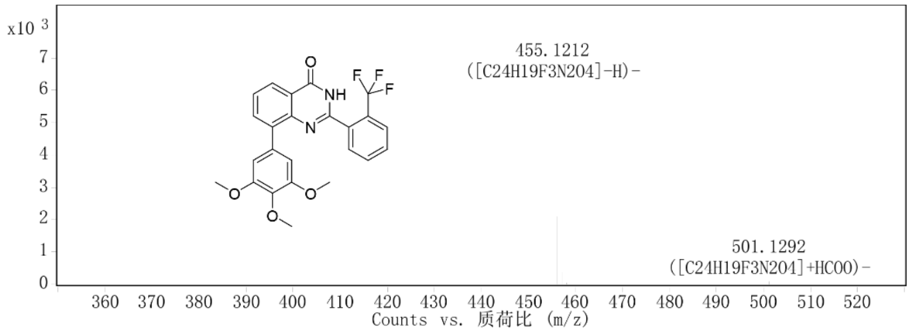

Figure S25: HR MS spectrum of A5.

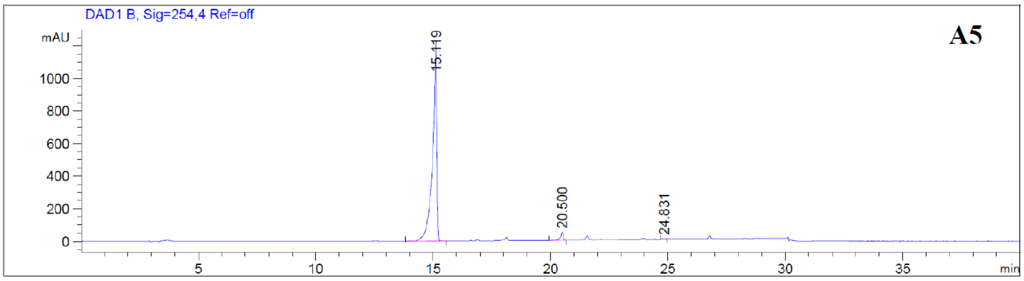

Signal 2: DAD1 B, Sig=254,4 Ref=off

Peak #	Retention time [min]	Type	Peak Width [min]	Peak area [mAU*s]	Peak height [mAU]	Peak area %
1	15.119	VB R	0.1609	1.49160e4	1234.78906	97.3808
2	20.500	BB	0.1159	393.29068	47.86544	2.5676
3	24.831	BB	0.1066	7.89247	1.14263	0.0515

Total amount: 1.53172e4 1283.79713

Figure S26: HPLC spectrum of A5.

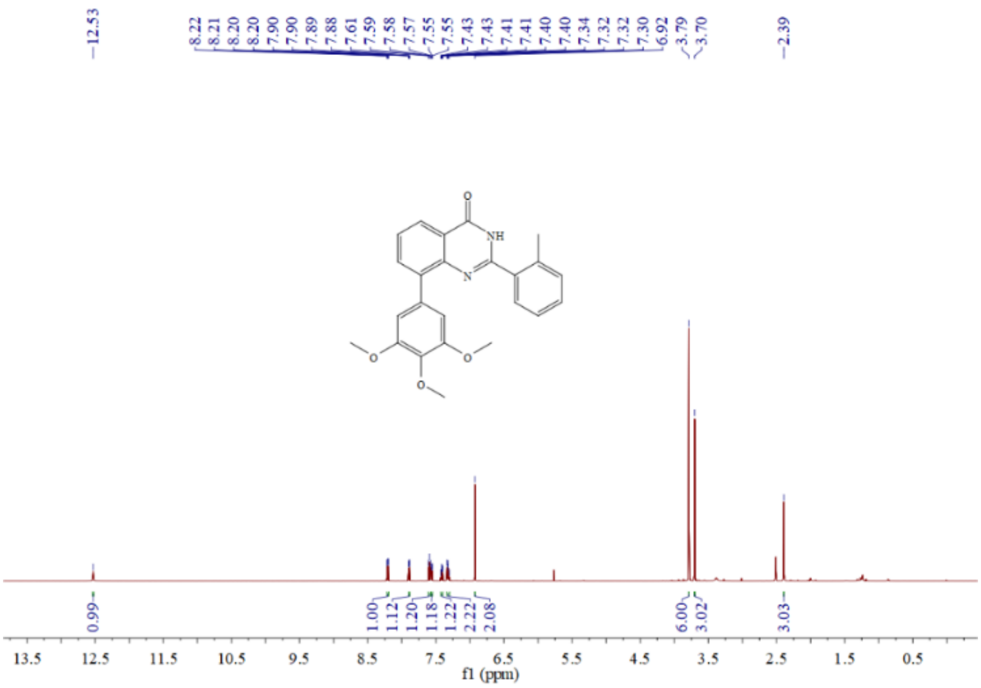

Figure S27: ^{1}H NMR spectrum of A6.

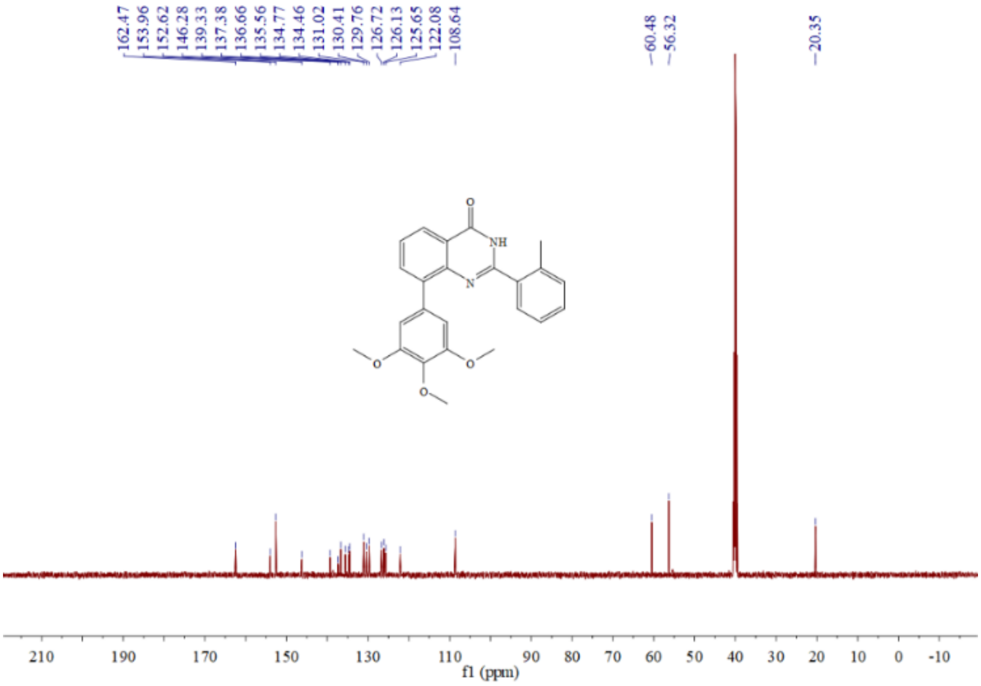

Figure S28: ^{13}C NMR spectrum of A6.

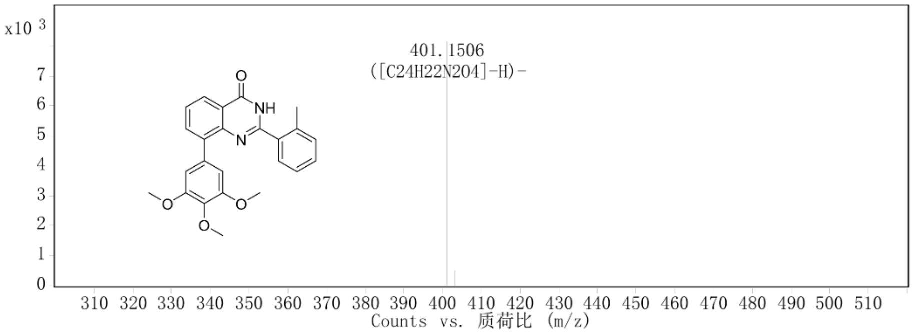

Figure S29: HR MS spectrum of A6.

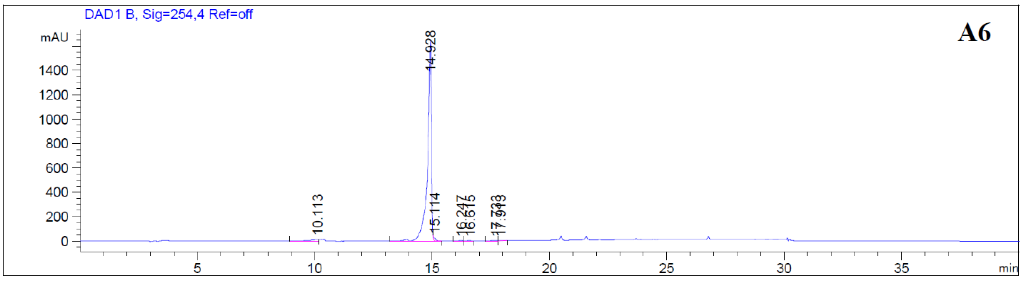

Signal 2: DAD1 B, Sig=254,4 Ref=off

Peak #	Retention time [min]	Type	Peak Width [min]	Peak area [mAU*s]	Peak height [mAU]	Peak area %
1	10.113	BV	0.3463	336.17511	12.47972	1.7837
2	14.928	VV R	0.1480	1.82801e4	1651.56262	96.9916
3	15.114	VB E	0.0850	136.68124	23.78471	0.7252
4	16.247	BV	0.1189	30.78428	3.63042	0.1633
5	16.615	VB	0.1480	24.24508	2.24558	0.1286
6	17.733	BV	0.1522	17.58724	1.55182	0.0933
7	17.913	VB	0.1435	21.51887	2.00073	0.1142
Total amount:				1.88471e4	1697.25560	

Figure S30: HPLC spectrum of A6.

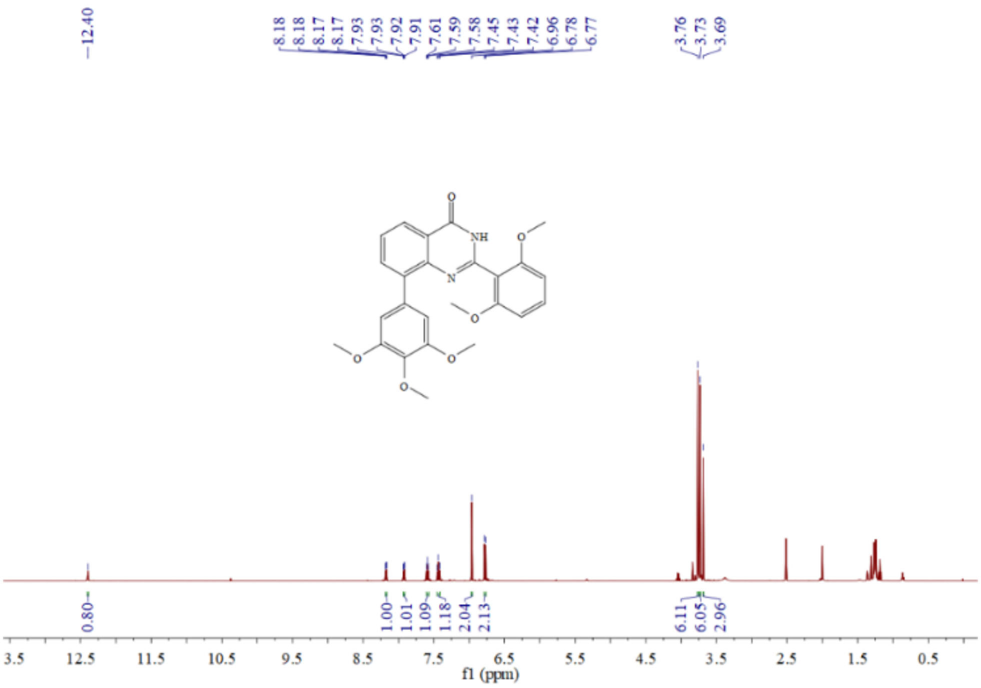

Figure S31: ^{1}H NMR spectrum of A7.

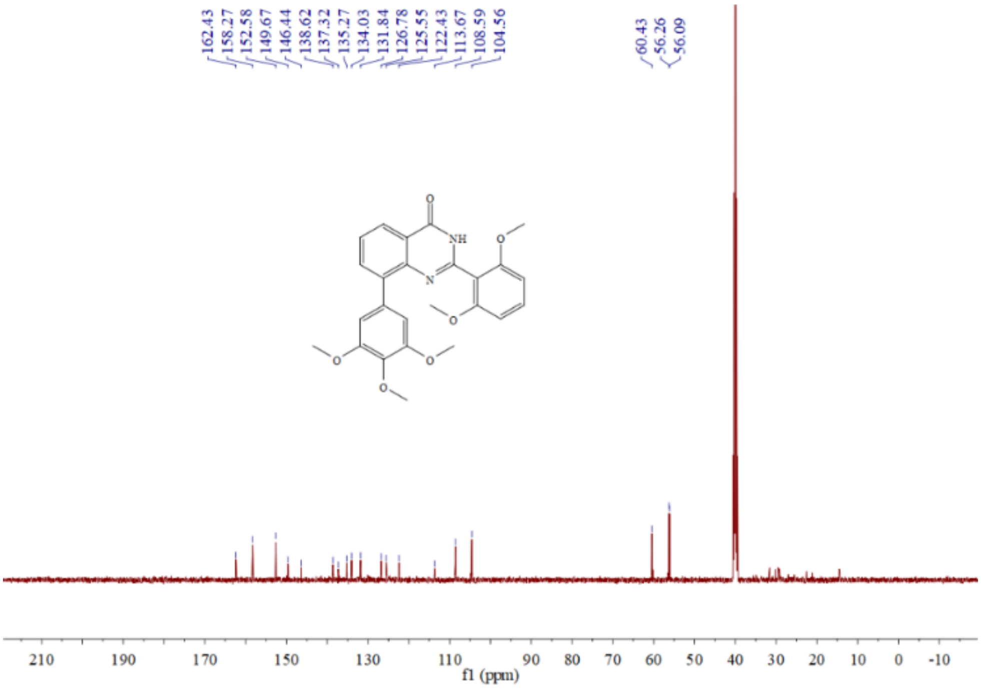

Figure S32: ^{13}C NMR spectrum of A7.

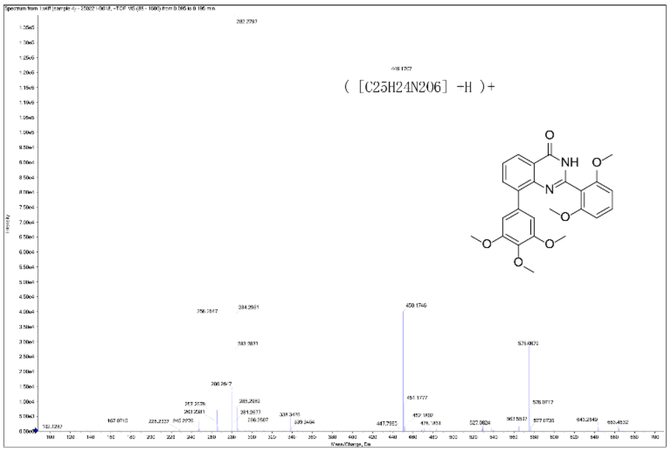

Figure S33: HR MS spectrum of A7.

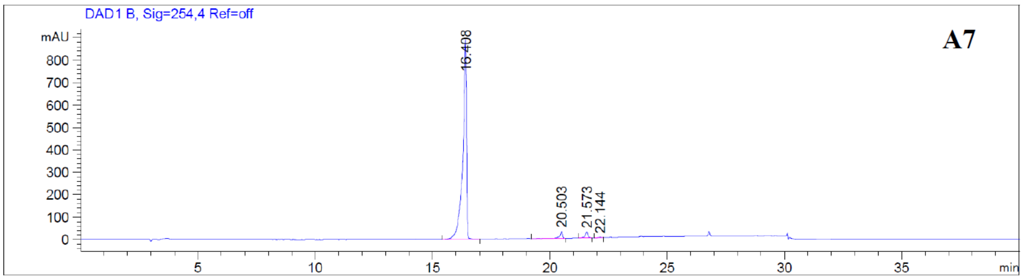

Signal 2: DAD1 B, Sig=254,4 Ref=off

Peak #	Retention time [min]	Type	Peak Width [min]	Peak area [mAU*s]	Peak height [mAU]	Peak area %
1	16.408	BV R	0.1455	9615.29492	892.91205	95.0322
2	20.503	VB R	0.1205	275.00085	29.88650	2.7180
3	21.573	BB	0.1119	211.11147	26.81009	2.0865
4	22.144	BB	0.1052	16.52444	2.21014	0.1633

Total amount: 1.01179e4 951.81878

Figure S34: HPLC spectrum of A7.

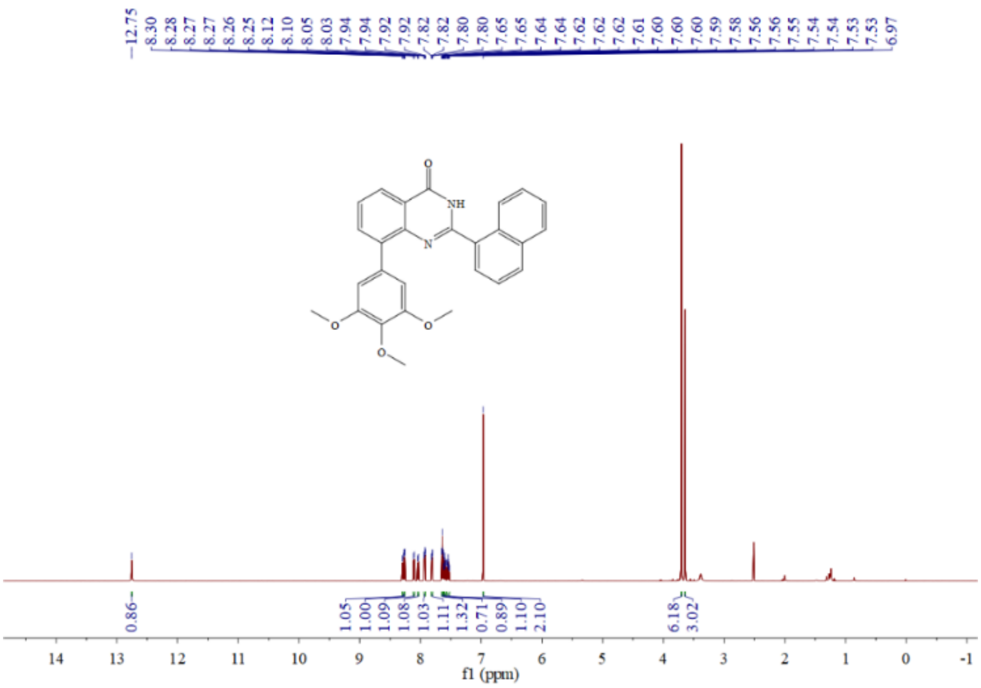

Figure S35: ^{1}H NMR spectrum of A8.

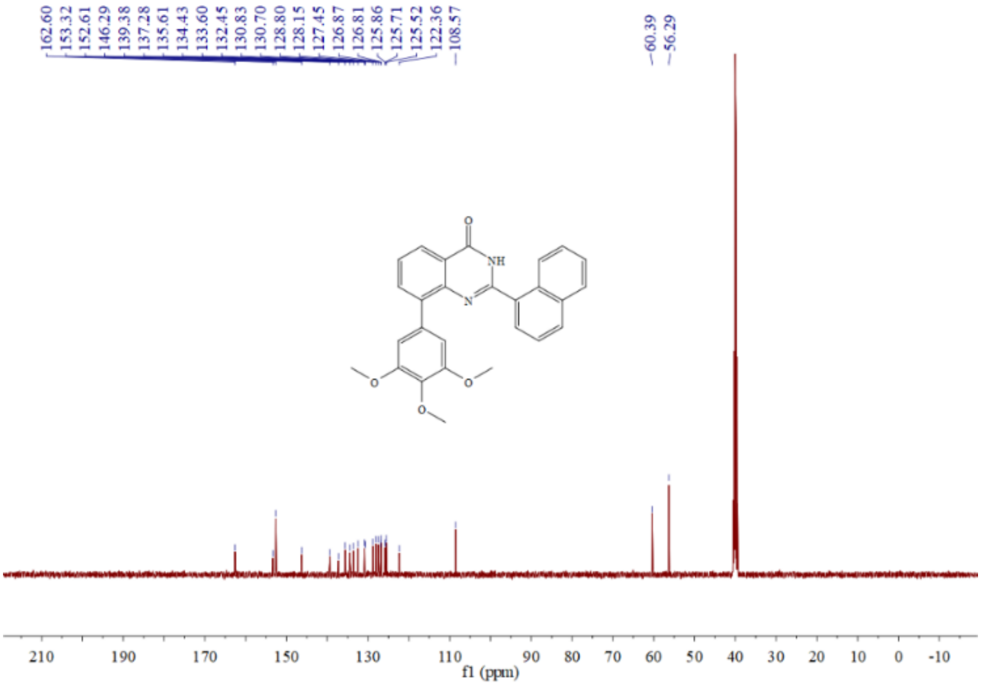

Figure S36: ^{13}C NMR spectrum of A8.

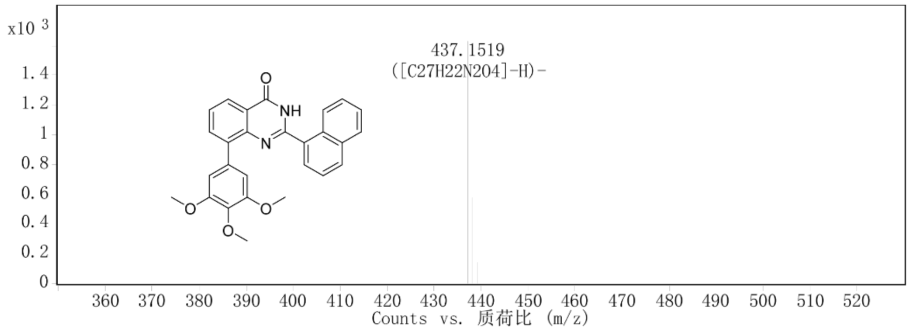

Figure S37: HR MS spectrum of A8.

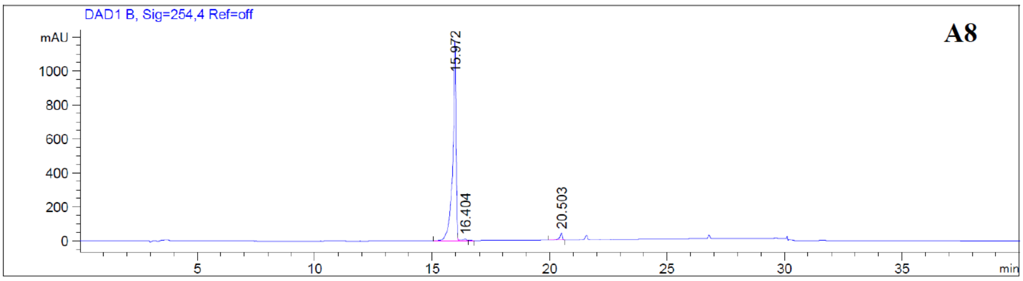

Signal 2: DAD1 B, Sig=254,4 Ref=off

Peak #	Retention time [min]	Type	Peak Width [min]	Peak area [mAU*s]	Peak height [mAU]	Peak area %
1	15.972	BV R	0.1326	1.14115e4	1180.97681	95.9028
2	16.404	VV E	0.1480	135.20341	12.31965	1.1363
3	20.503	BB	0.1153	352.32571	43.13621	2.9610

Total amount: 1.18991e4 1236.43267

Figure S38: HPLC spectrum of A8.

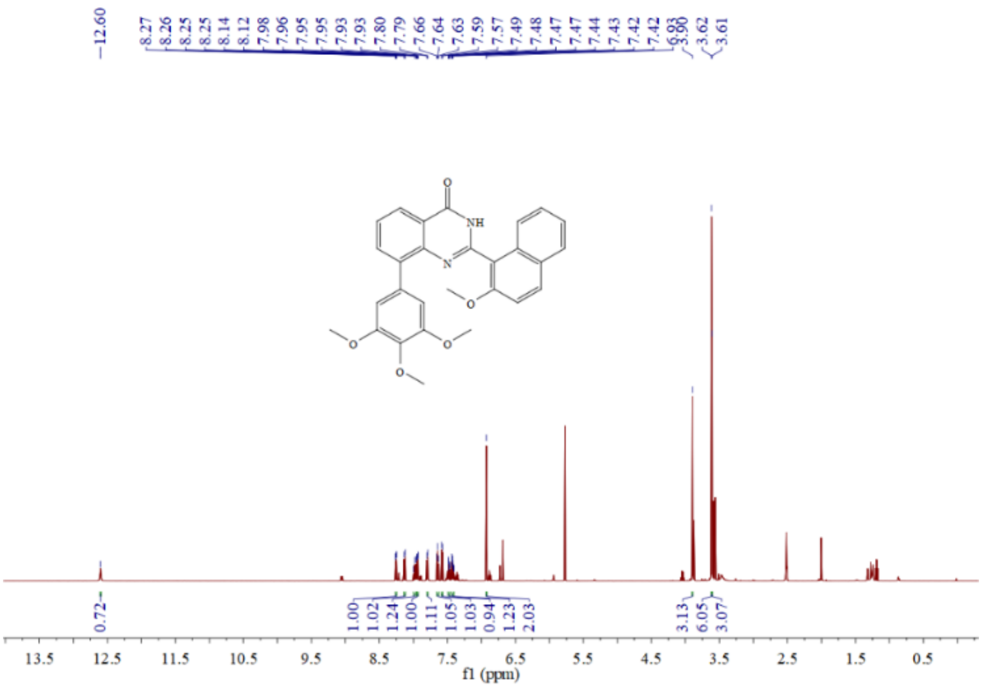

Figure S39: ^{1}H NMR spectrum of A9.

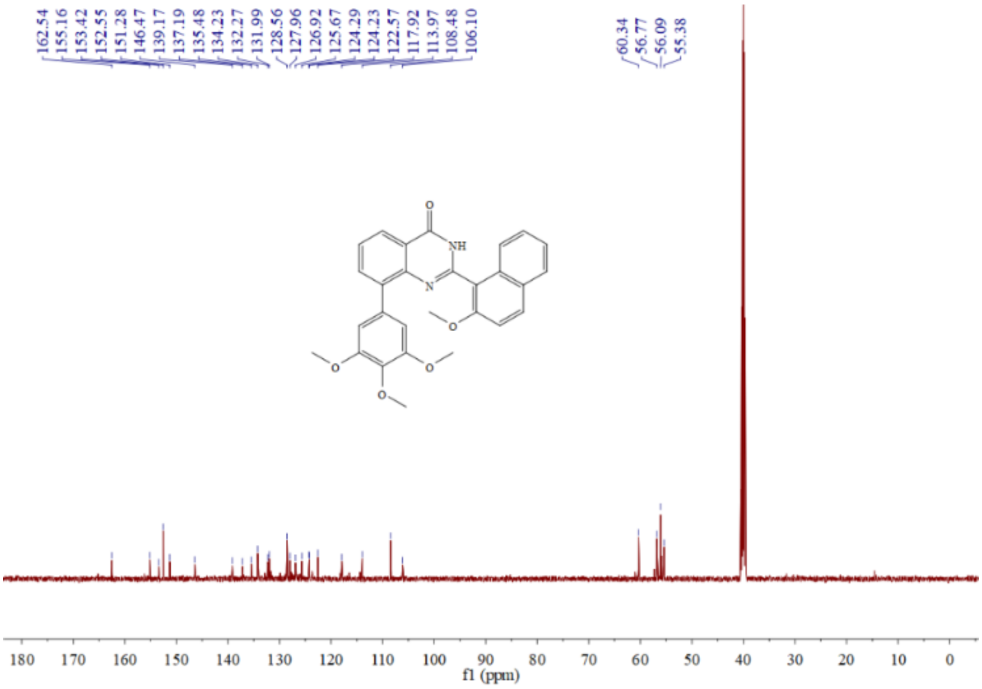

Figure S40: ^{13}C NMR spectrum of A9.

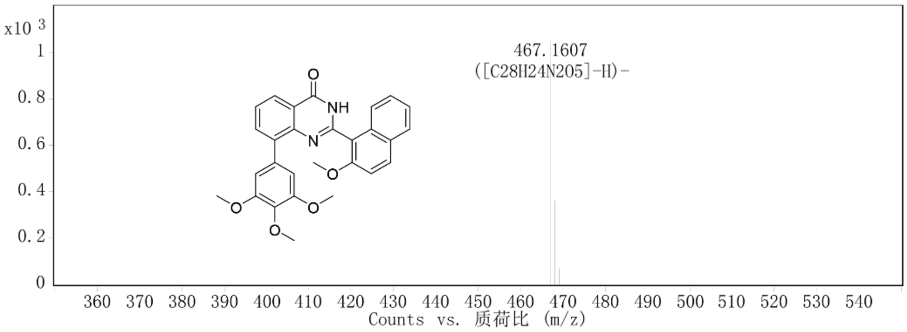

Figure S41: HR MS spectrum of A9.

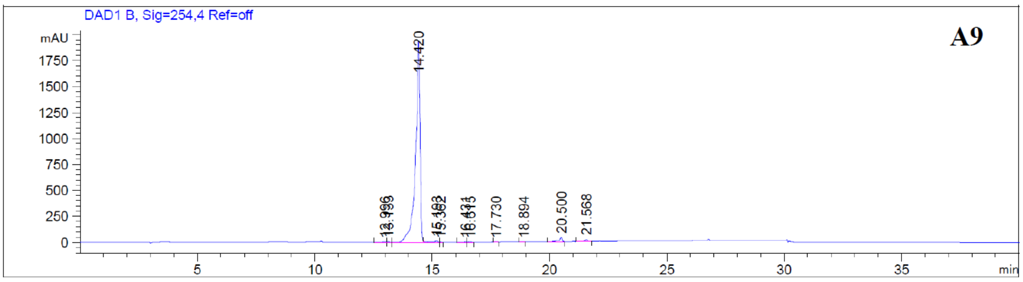

Signal 2: DAD1 B, Sig=254.4 Ref=off

Peak #	Retention time [min]	Type	Peak Width [min]	Peak area [mAU*s]	Peak height [mAU]	Peak area %
1	12.996	BV	0.1658	65.78774	5.41172	0.2526
2	13.133	VB	0.0902	40.93852	6.61672	0.1572
3	14.420	BV R	0.1774	2.51770e4	1940.23926	96.6646
4	15.193	VV E	0.1797	147.75516	10.65927	0.5673
5	15.362	VB E	0.0813	8.66841	1.64930	0.0333
6	16.431	BV	0.1388	16.86482	1.62827	0.0648
7	16.615	VB	0.1420	18.23220	1.83433	0.0700
8	17.730	BB	0.0971	6.76738	1.08076	0.0260
9	18.894	BB	0.0923	10.62002	1.76305	0.0408
10	20.500	BB	0.1235	396.17950	45.51863	1.5211
11	21.568	BB	0.1129	156.91025	19.71293	0.6024
Total amount:				2.60458e4	2036.11425	

Figure S42: HPLC spectrum of A9.

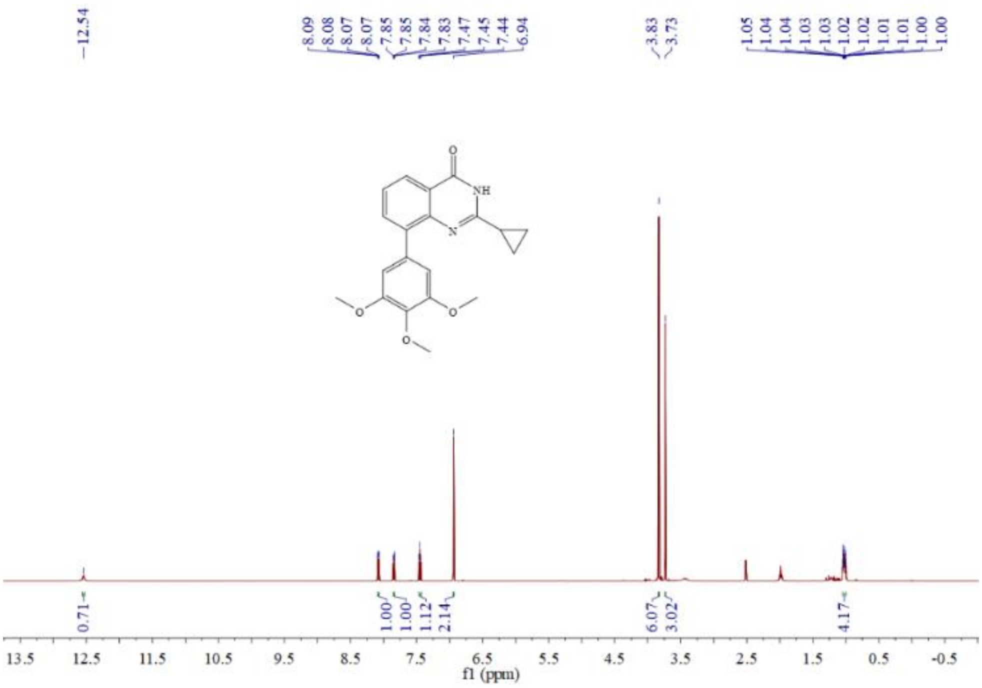

Figure S43: ^{1}H NMR spectrum of A10.

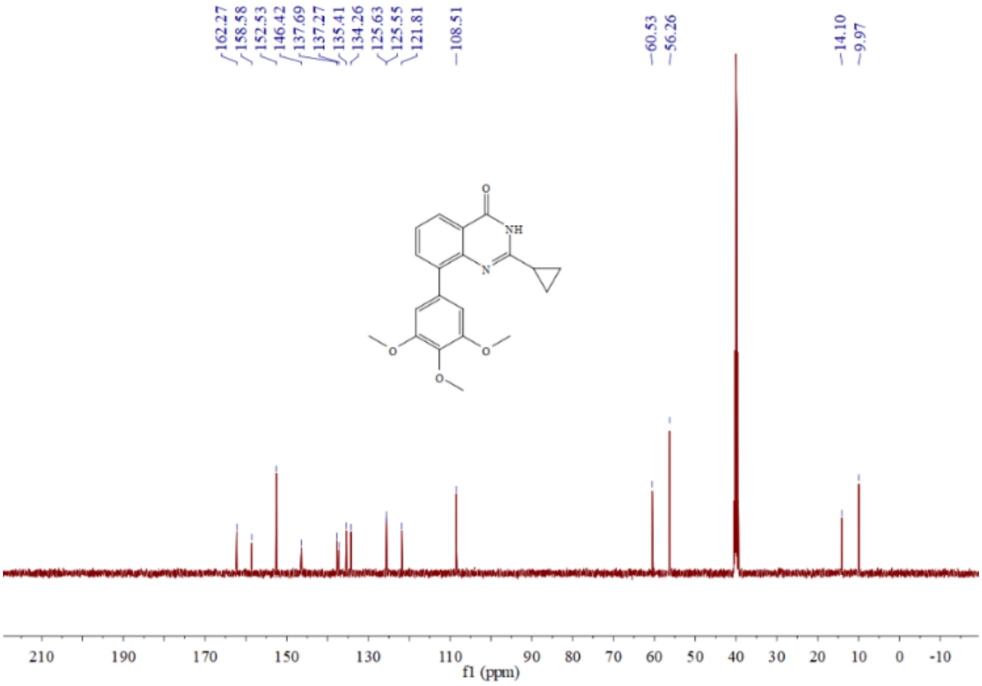

Figure S44: ^{13}C NMR spectrum of A10.

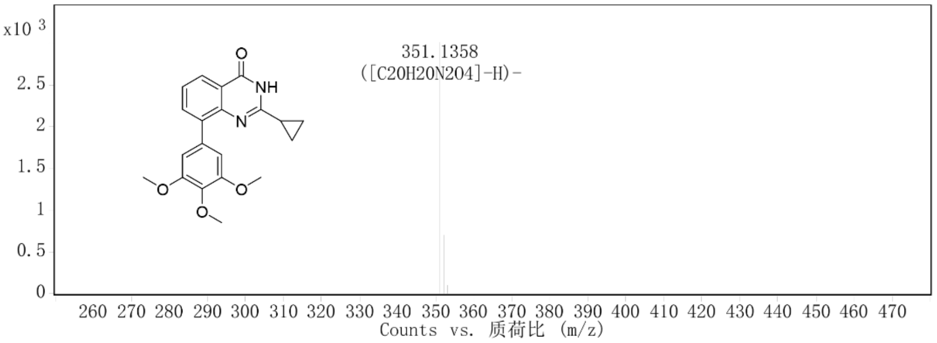

Figure S45: HR MS spectrum of A10.

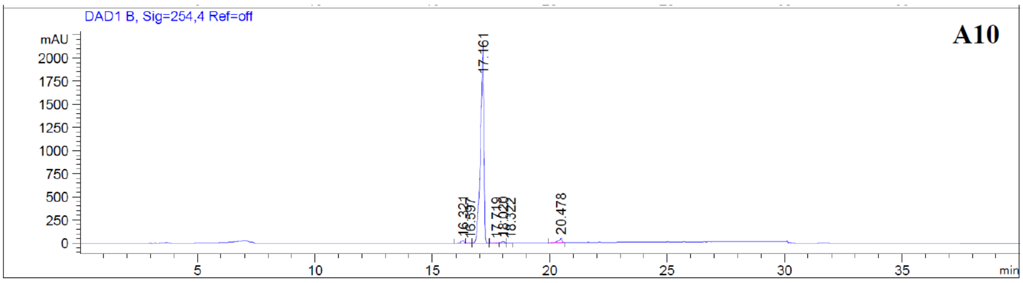

Signal 2: DAD1 B, Sig=254,4 Ref=off

Peak #	Retention time [min]	Type	Peak Width [min]	Peak area [mAU*s]	Peak height [mAU]	Peak area %
1	16.321	BV R	0.1294	255.40665	26.74943	1.0940
2	16.597	VV E	0.1396	29.45311	3.02490	0.1262
3	17.161	VB	0.1405	2.24194e4	2171.35767	96.0275
4	17.719	BV E	0.1143	12.11738	1.56598	0.0519
5	18.020	VB R	0.1368	148.43626	15.35086	0.6358
6	18.322	BV	0.0954	6.68321	1.06227	0.0286
7	20.478	VB R	0.1345	475.35669	47.61600	2.0361
Total amount:				2.33468e4	2266.72711	

Figure S46: HPLC spectrum of A10.

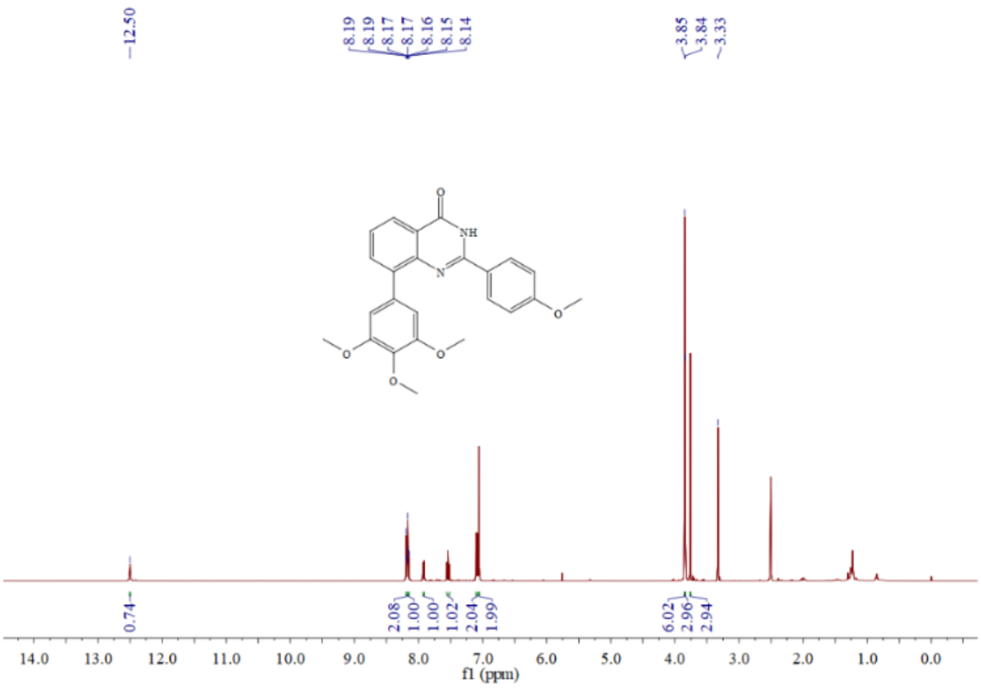

Figure S47: ^{1}H NMR spectrum of A11.

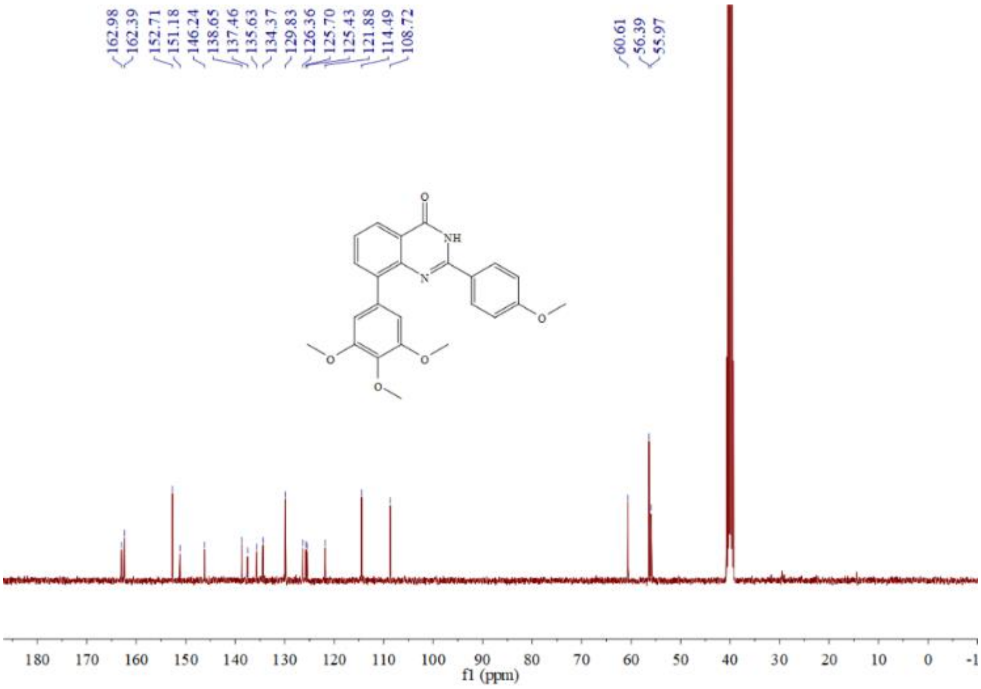

Figure S48: ^{13}C NMR spectrum of A11.

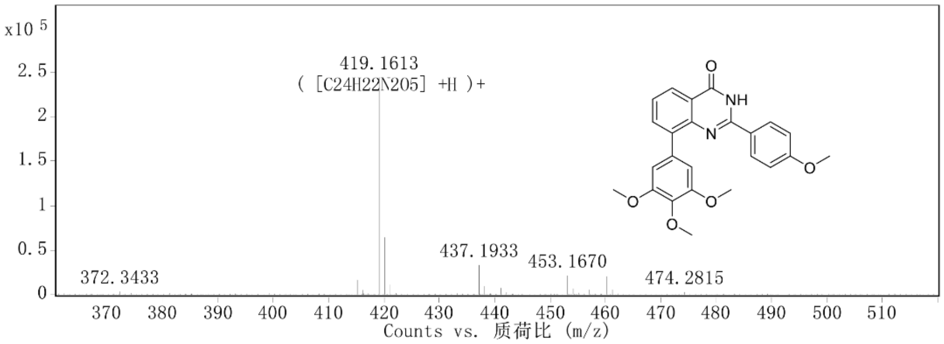

Figure S49: HR MS spectrum of A11.

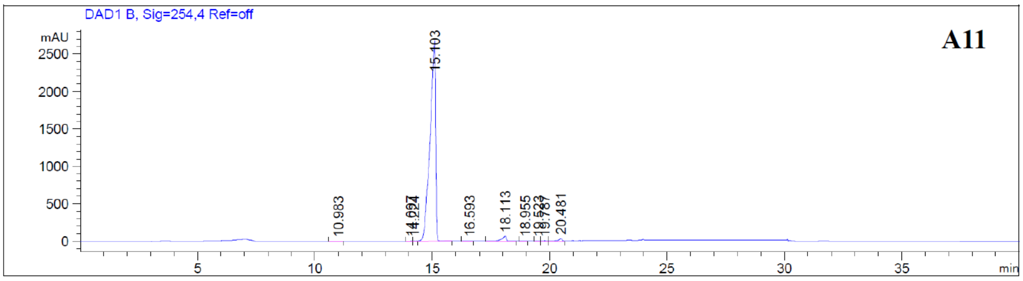

Signal 2: DAD1 B, Sig=254,4 Ref=off

Peak #	Retention time [min]	Type	Peak Width [min]	Peak area [mAU*s]	Peak height [mAU]	Peak area %
1	10.983	BB	0.2085	20.80221	1.31516	0.0448
2	14.097	BV E	0.1226	11.15145	1.17653	0.0240
3	14.224	VV E	0.0883	7.60610	1.19472	0.0164
4	15.103	VV R	0.2283	4.52169e4	2687.38501	97.3160
5	16.593	BB	0.1491	21.96426	2.04799	0.0473
6	18.113	BV R	0.1729	859.77869	65.60978	1.8504
7	18.955	BV	0.1091	17.88493	2.39529	0.0385
8	19.523	VV	0.1623	12.92022	1.05951	0.0278
9	19.787	VB	0.1103	17.90461	2.36615	0.0385
10	20.481	VB R	0.1125	277.07721	34.93779	0.5963

Total amount: 4.64639e4 2799.48793

Figure S50: HPLC spectrum of A11.

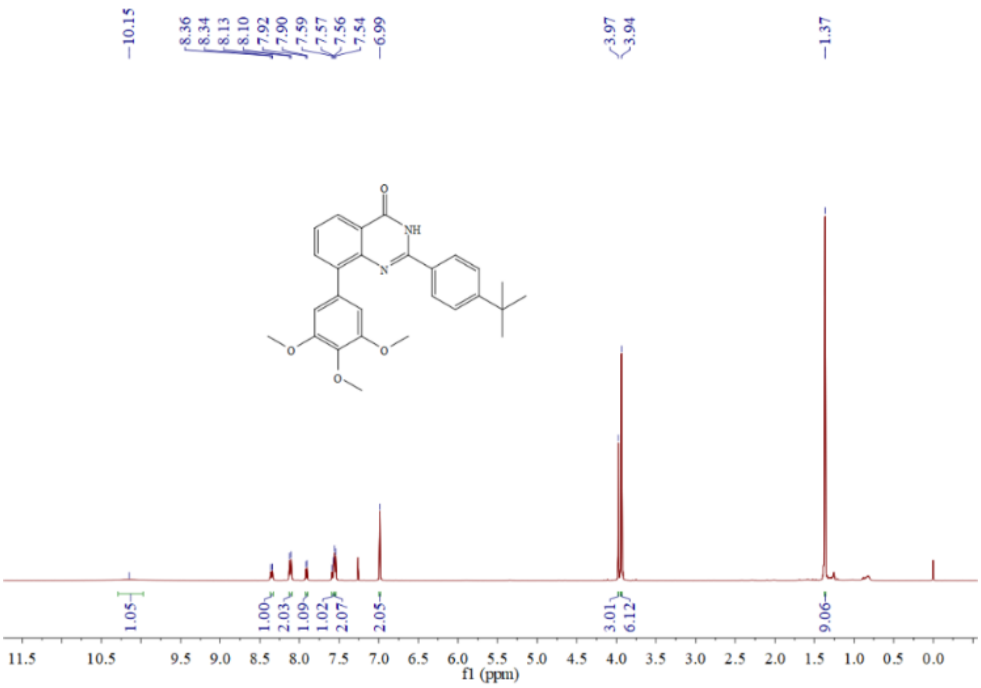

Figure S51: ^{1}H NMR spectrum of A12.

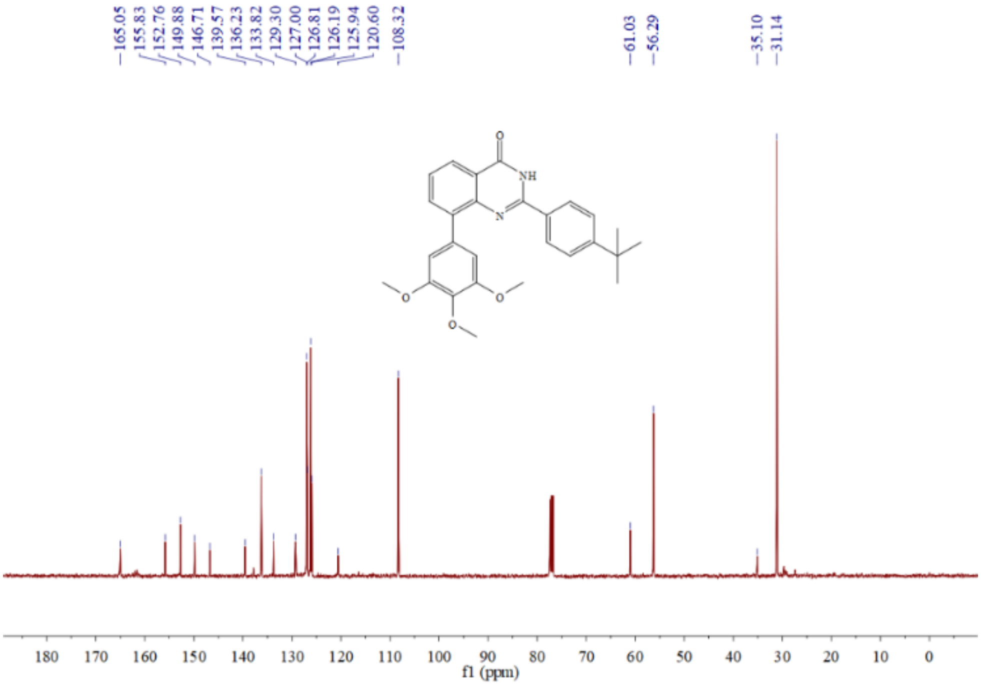

Figure S52: ^{13}C NMR spectrum of A12.

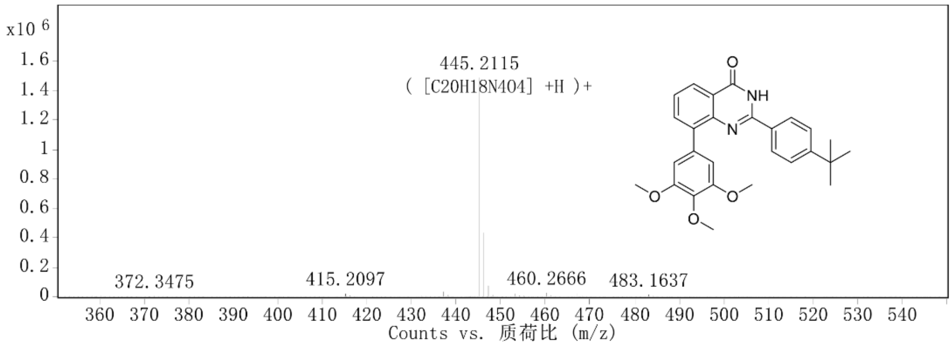

Figure S53: HR MS spectrum of A12.

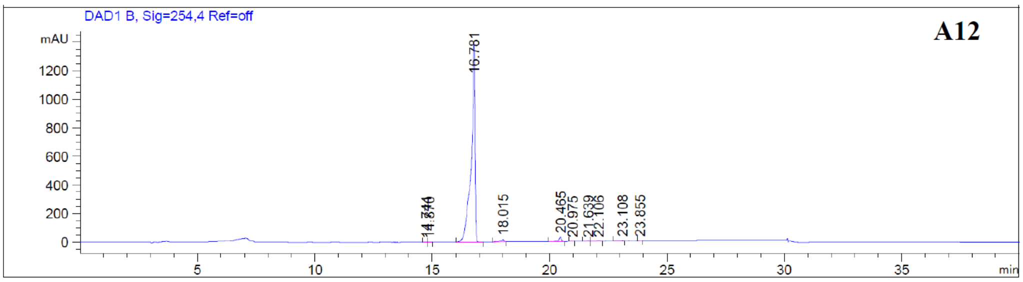

Signal 2: DAD1 B, Sig=254,4 Ref=off

Peak #	Retention time [min]	Type	Peak Width [min]	Peak area [mAU*s]	Peak height [mAU]	Peak area %
1	14.744	BV	0.0916	9.09310	1.40404	0.0573
2	14.870	VB	0.0879	11.32876	1.89046	0.0714
3	16.781	BB	0.1479	1.53829e4	1402.68115	96.8973
4	18.015	BB	0.1396	157.55989	15.63540	0.9925
5	20.465	BB	0.1149	243.57735	30.58859	1.5343
6	20.975	BB	0.0876	6.42043	1.10877	0.0404
7	21.639	BB	0.1076	21.29154	2.97213	0.1341
8	22.106	BB	0.1144	19.04296	2.35449	0.1200
9	23.108	BB	0.1427	17.41348	1.65604	0.1097
10	23.855	BB	0.0742	6.83950	1.42066	0.0431
Total amount:				1.58755e4	1461.71173	

Figure S54: HPLC spectrum of A12.

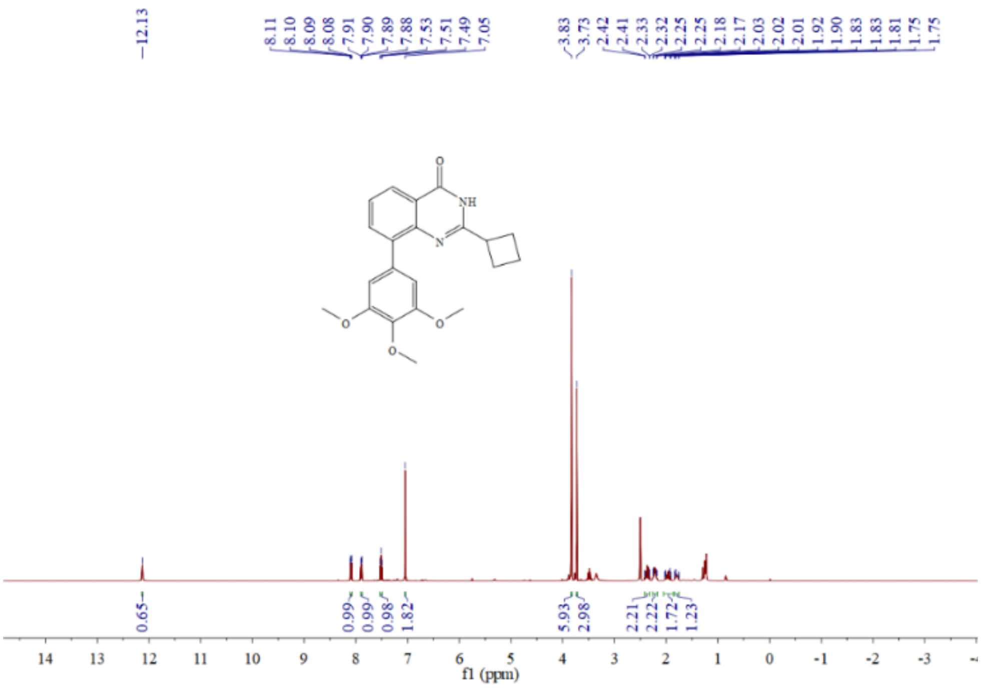

Figure S55: ^{1}H NMR spectrum of A13.

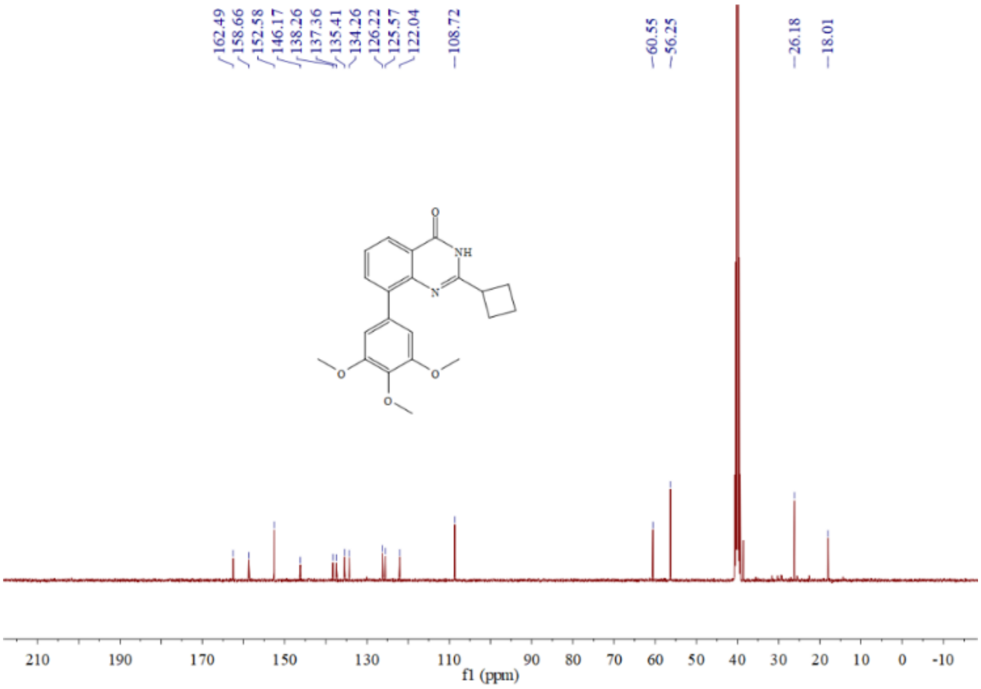

Figure S56: ^{13}C NMR spectrum of A13.

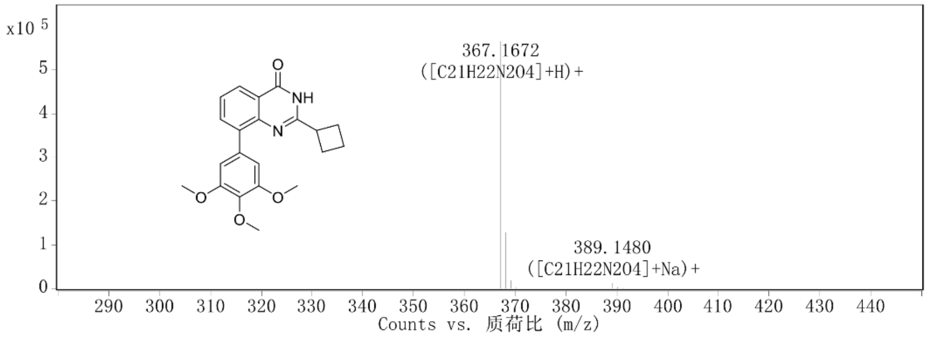

Figure S57: HR MS spectrum of A13.

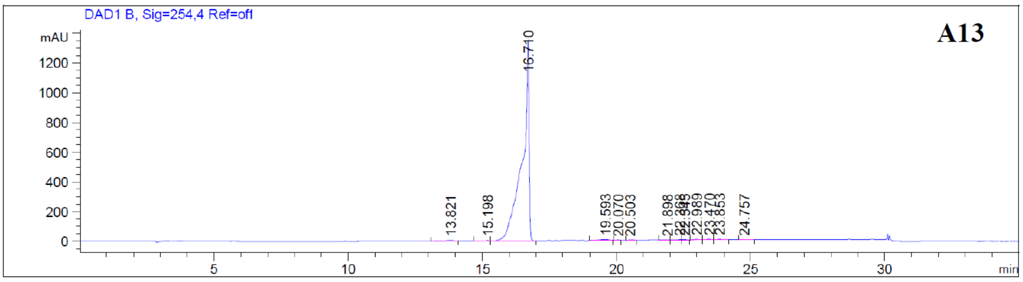

Signal 2: DAD1 B, Sig=254,4 Ref=off

Peak #	Retention time [min]	Type	Peak Width [min]	Peak area [mAU*s]	Peak height [mAU]	Peak area %
1	13.821	BB	0.2906	94.31142	4.32249	0.3884
2	15.198	BB	0.1661	41.92266	3.39523	0.1726
3	16.710	BB	0.2254	2.38083e4	1352.23547	98.0375
4	19.593	BB	0.3013	127.74716	5.26027	0.5260
5	20.070	BB	0.1073	8.35657	1.09120	0.0344
6	20.503	BB	0.1114	19.68441	2.45767	0.0811
7	21.898	BB	0.1314	18.32436	1.88599	0.0755
8	22.368	BV	0.1680	21.33101	1.80391	0.0878
9	22.545	VB	0.1329	18.30277	1.89173	0.0754
10	22.989	BV	0.1752	43.98082	3.30617	0.1811
11	23.470	VB	0.1752	35.79506	2.65614	0.1474
12	23.853	BB	0.1084	31.72130	4.00602	0.1306
13	24.757	BB	0.1513	15.11924	1.36432	0.0623
Total amount:				42849e4	1385.67662	

Figure S58: HPLC spectrum of A13.

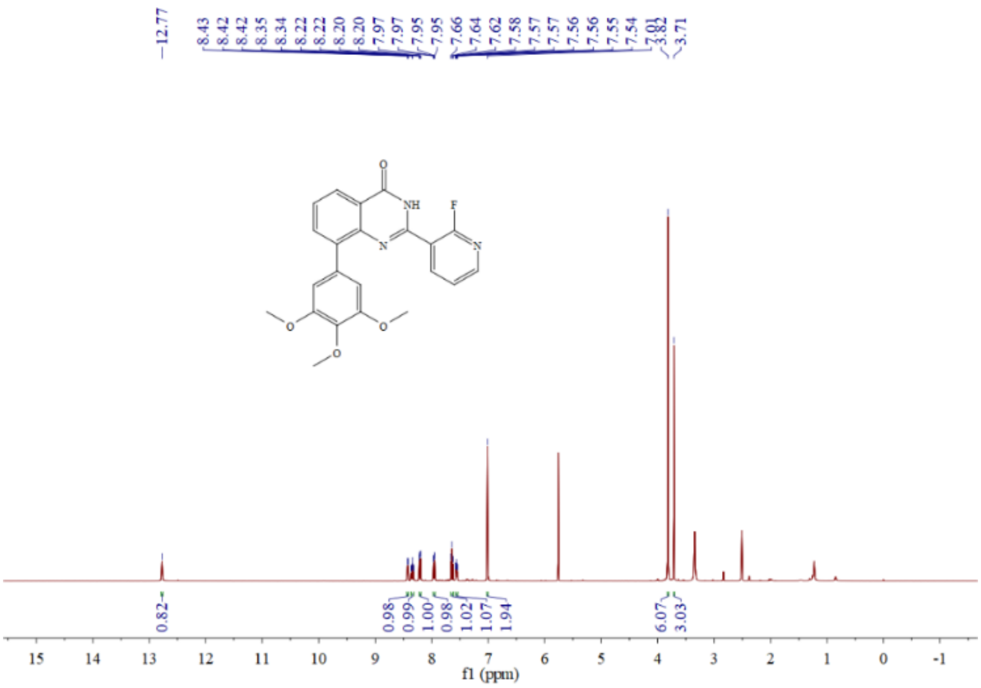

Figure S59: ^{1}H NMR spectrum of A14.

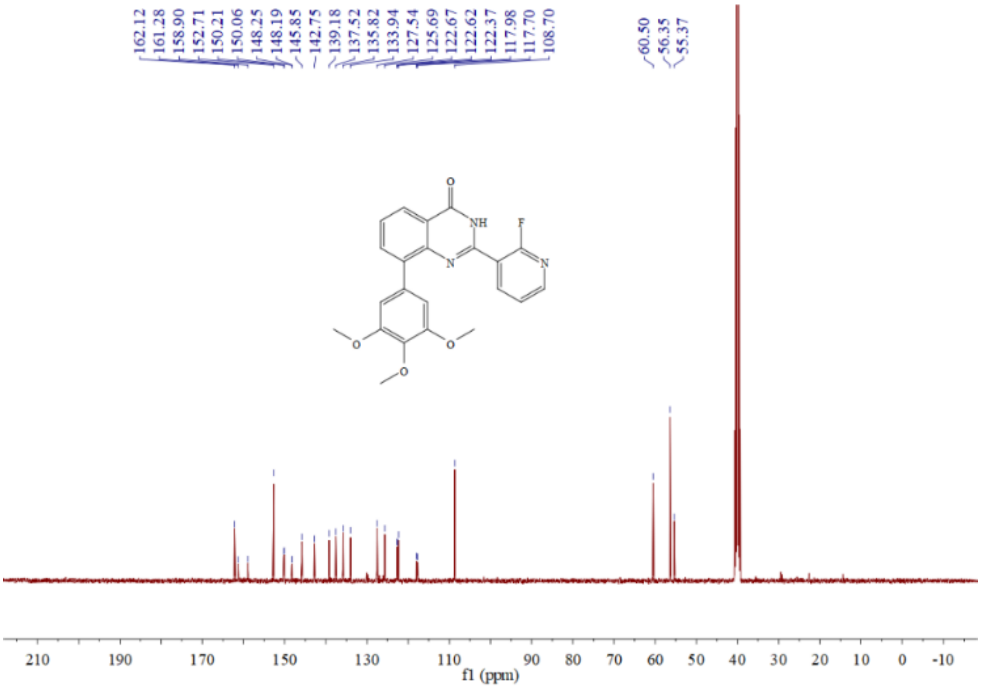

Figure S60: ^{13}C NMR spectrum of A14.

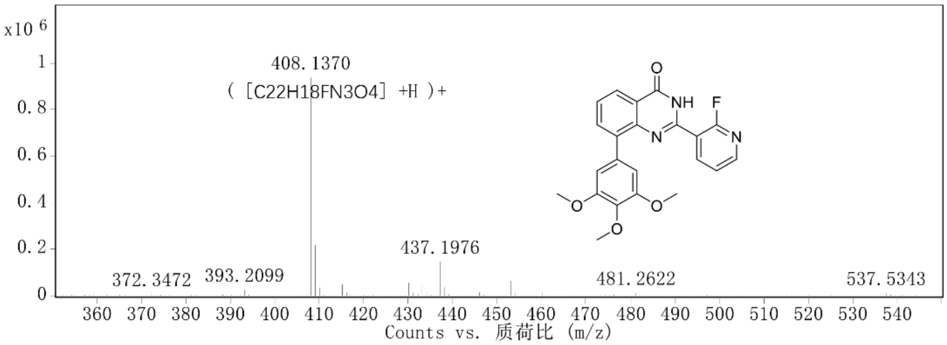

Figure S61: HR MS spectrum of A14.

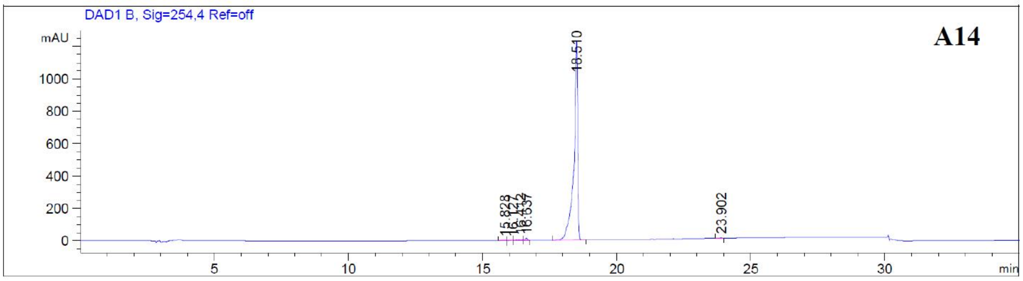

Signal 2: DAD1 B, Sig=254,4 Ref=off

Peak #	Retention time [min]	Type	Peak Width [min]	Peak area [mAU*s]	Peak height [mAU]	Peak area %
1	15.828	BB	0.1072	13.51790	1.76900	0.1091
2	16.127	BV	0.1007	13.02386	1.88345	0.1051
3	16.412	VV	0.1542	87.80063	7.63103	0.7086
4	16.637	VB	0.1047	102.19975	14.07238	0.8248
5	18.510	BB	0.1351	1.21602e4	1232.39417	98.1348
6	23.902	BB	0.0822	14.58009	2.65033	0.1177

Total amount: 1.23913e4 1260.40035

Figure S62: HPLC spectrum of A14.

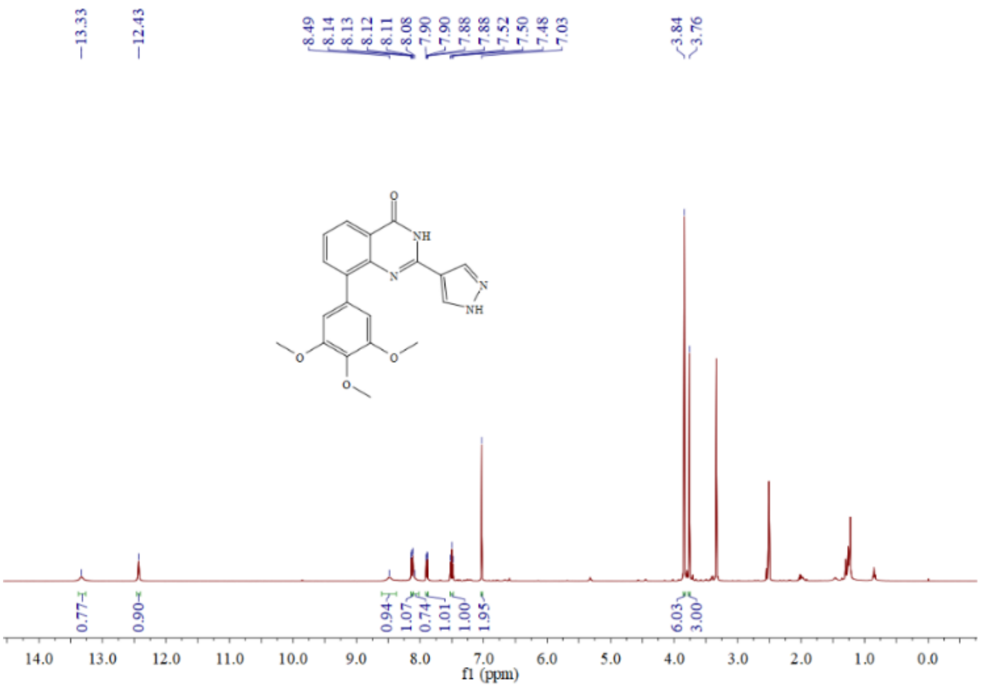

Figure S63: ^{1}H NMR spectrum of A15.

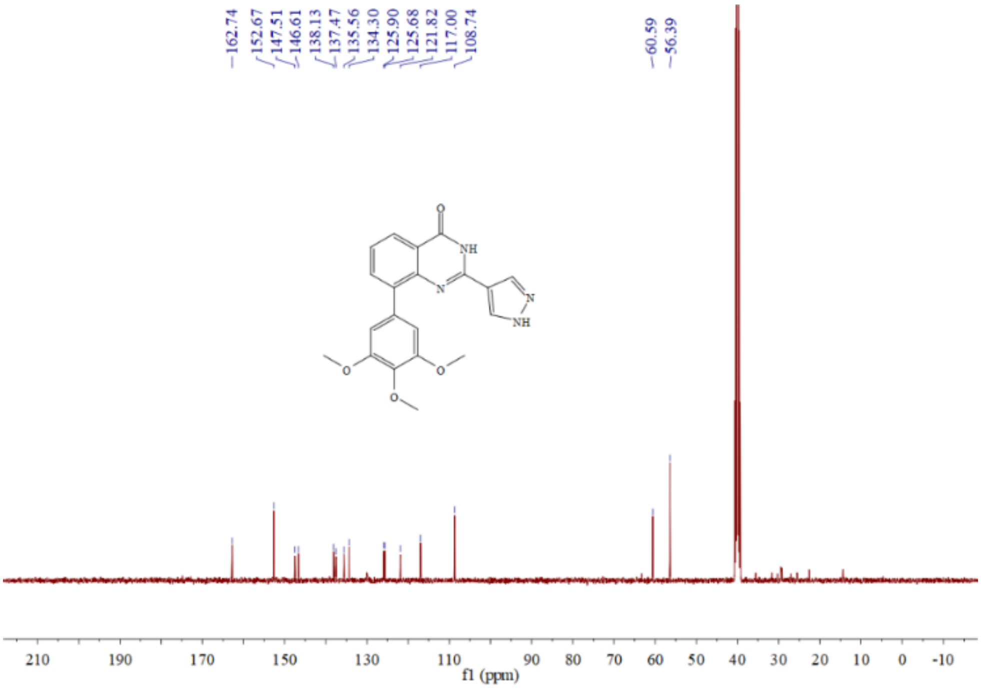

Figure S64: ^{13}C NMR spectrum of A15.

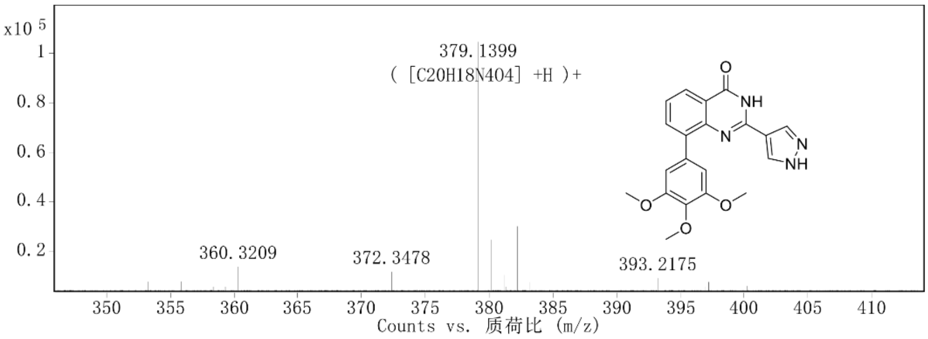

Figure S65: HR MS spectrum of A15.

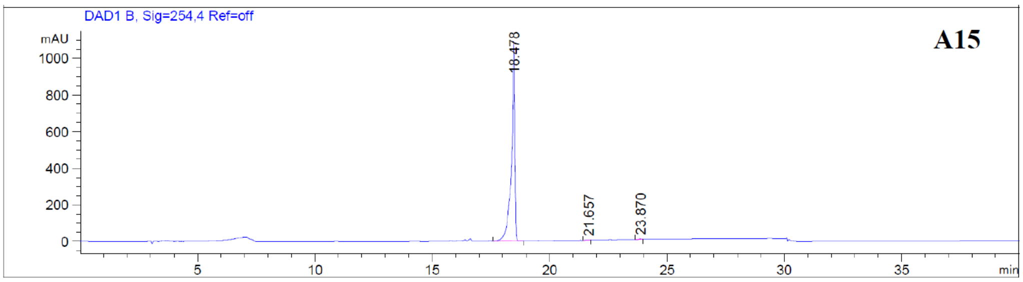

Signal 2: DAD1 B, Sig=254,4 Ref=off

Peak #	Retention time [min]	Type	Peak Width [min]	Peak area [mAU*s]	Peak height [mAU]	Peak area %
1	18.478	BB	0.1365	1.07152e4	1092.14417	99.8012
2	21.657	BV	0.1178	8.75157	1.08896	0.0815
3	23.870	BB	0.0799	12.59071	2.45212	0.1173

Total amount: 1.07365e4 1095.68525

Figure S66: HPLC spectrum of A15.

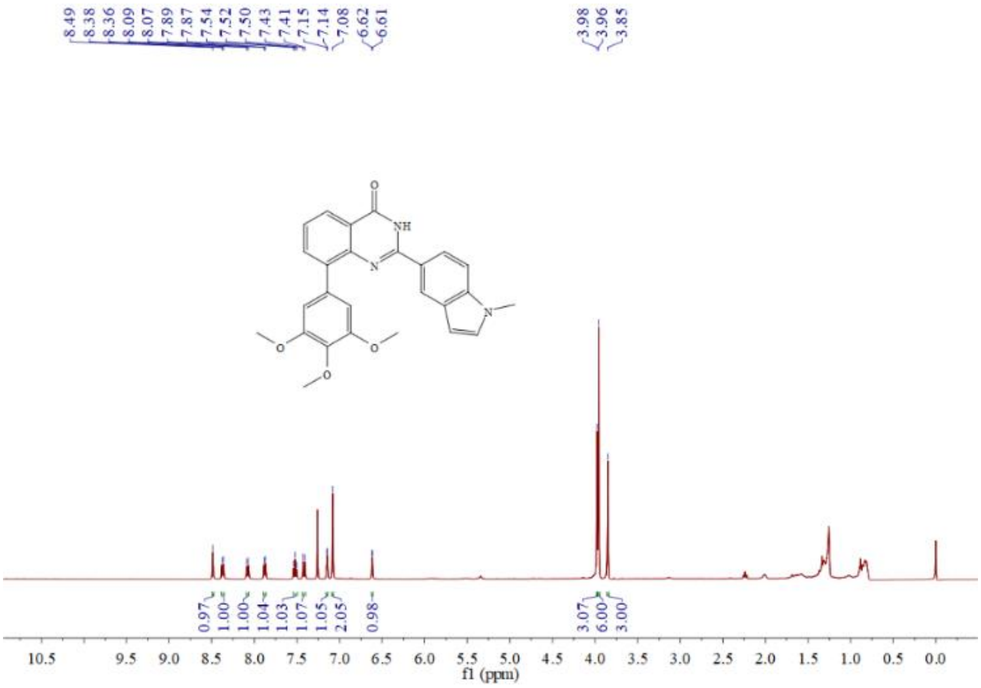

Figure S67: ^{1}H NMR spectrum of A16.

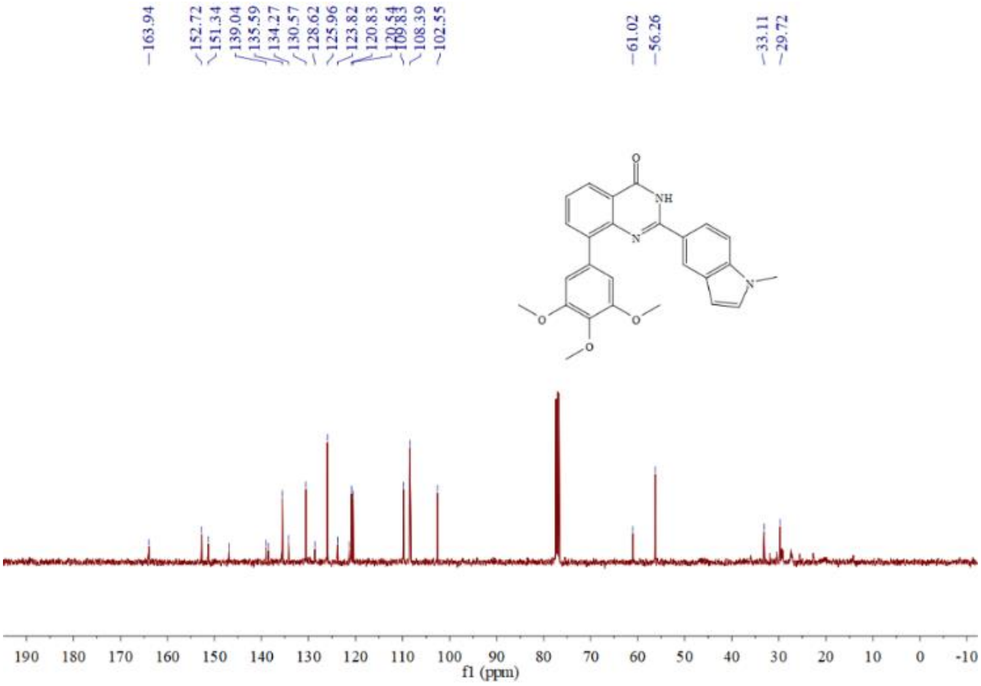

Figure S68: ^{13}C NMR spectrum of A16.

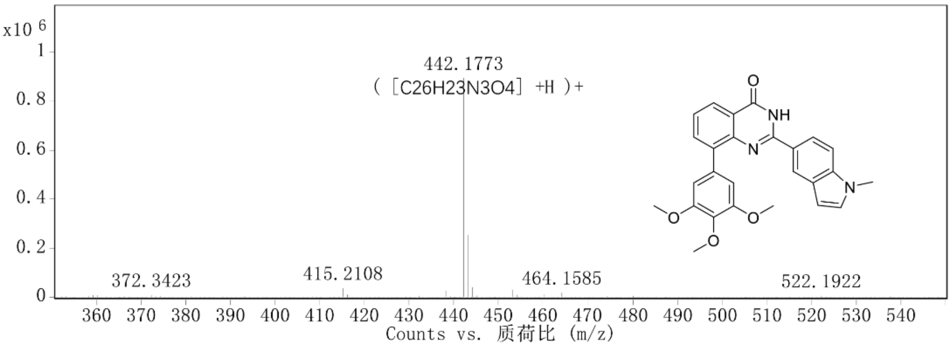

Figure S69: HR MS spectrum of A16.

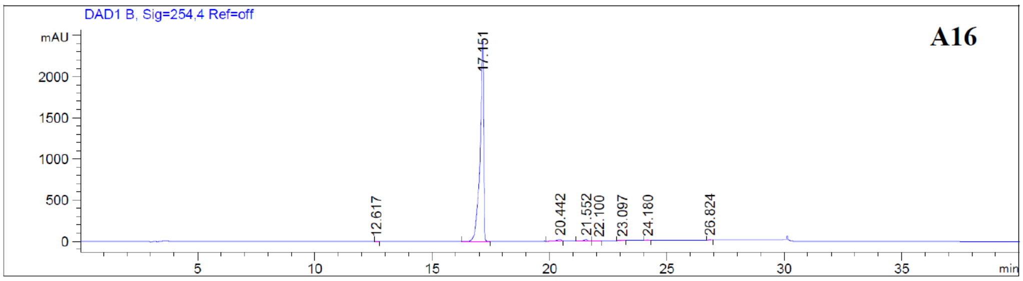

Signal 2: DAD1 B, Sig=254,4 Ref=off

Peak #	Retention time [min]	Type	Peak Width [min]	Peak area [mAU*s]	Peak height [mAU]	Peak area %
1	12.617	BB	0.0664	7.99108	1.84931	0.0297
2	17.151	BB	0.1540	2.63793e4	2442.61621	98.1533
3	20.442	BB	0.1272	220.55878	24.45373	0.8207
4	21.552	BV R	0.1077	139.68219	18.58336	0.5197
5	22.100	BB	0.1409	12.28273	1.18514	0.0457
6	23.097	BB	0.1304	18.07571	1.87613	0.0673
7	24.180	VB	0.0876	23.67496	4.08418	0.0881
8	26.824	BB	0.0789	74.04156	14.67603	0.2755
Total amount:				2.68756e4	2509.32408	

Figure S70: HPLC spectrum of A16.

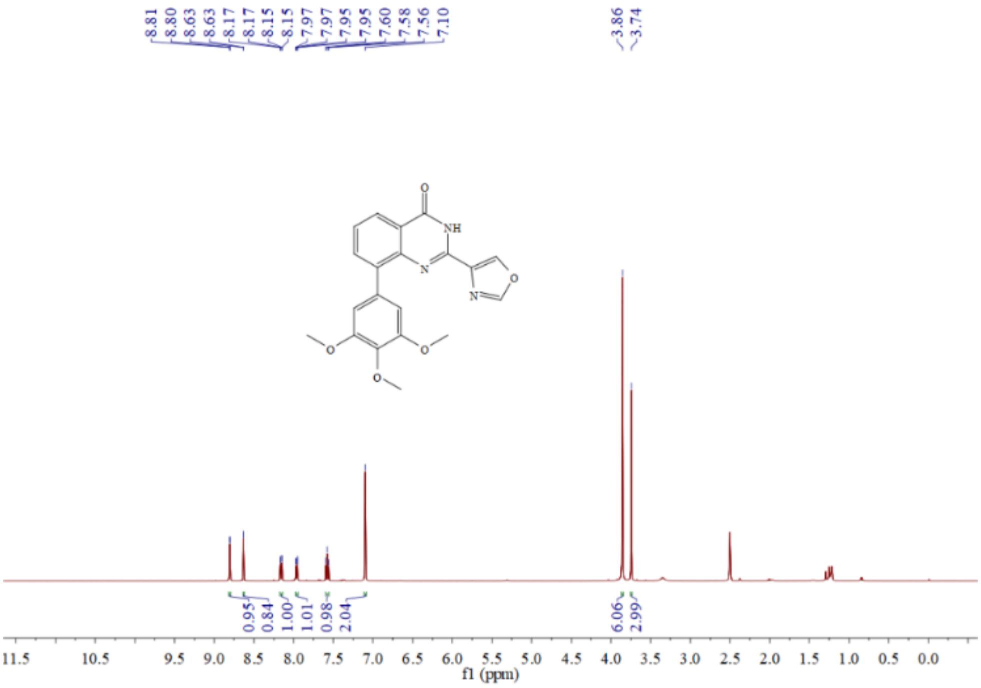

Figure S71: ^{1}H NMR spectrum of A17.

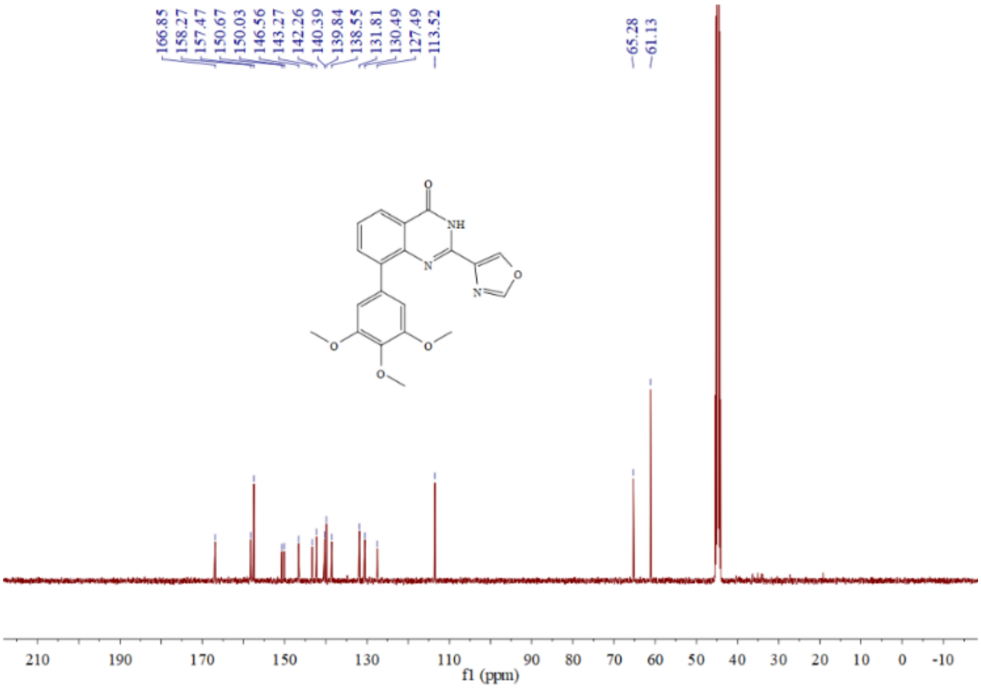

Figure S72: ^{13}C NMR spectrum of A17.

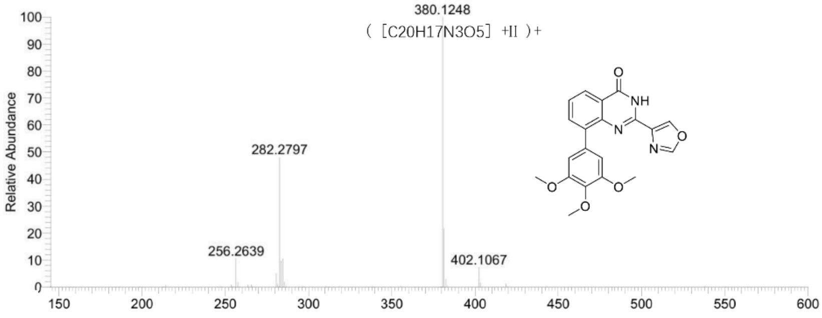

Figure S73: HR MS spectrum of A17.

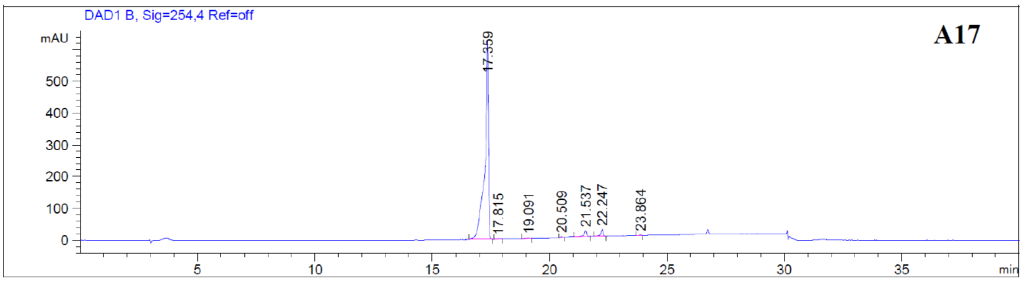

Signal 2: DAD1 B, Sig=254,4 Ref=off

Peak #	Retention time [min]	Type	Peak Width [min]	Peak area [mAU*s]	Peak height [mAU]	Peak area %
1	17.359	BB	0.1436	6626.29590	625.42896	95.3400
2	17.815	BB	0.0966	8.54750	1.30263	0.1230
3	19.091	BB	0.1078	13.37630	1.77838	0.1925
4	20.509	BB	0.0967	10.92456	1.66099	0.1572
5	21.537	BB	0.1075	141.85359	18.91966	2.0410
6	22.247	BB	0.1000	143.38193	20.91686	2.0630
7	23.864	BB	0.0846	5.79286	1.01513	0.0833
Total amount:				6950.17263	671.02260	

Figure S74: HPLC spectrum of A17.

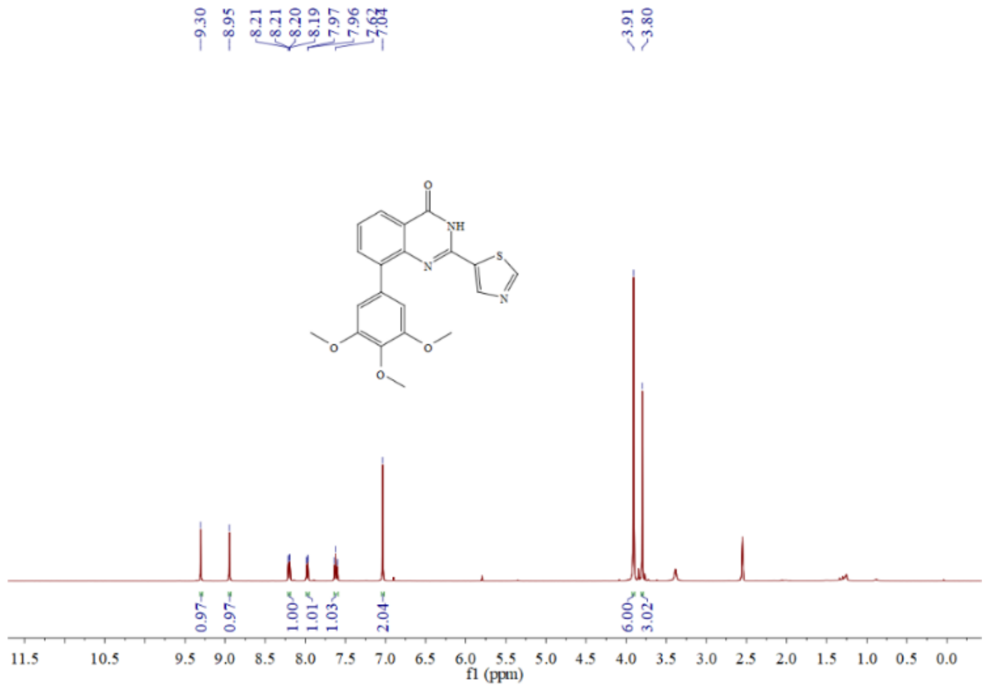

Figure S75: ^{1}H NMR spectrum of A18.

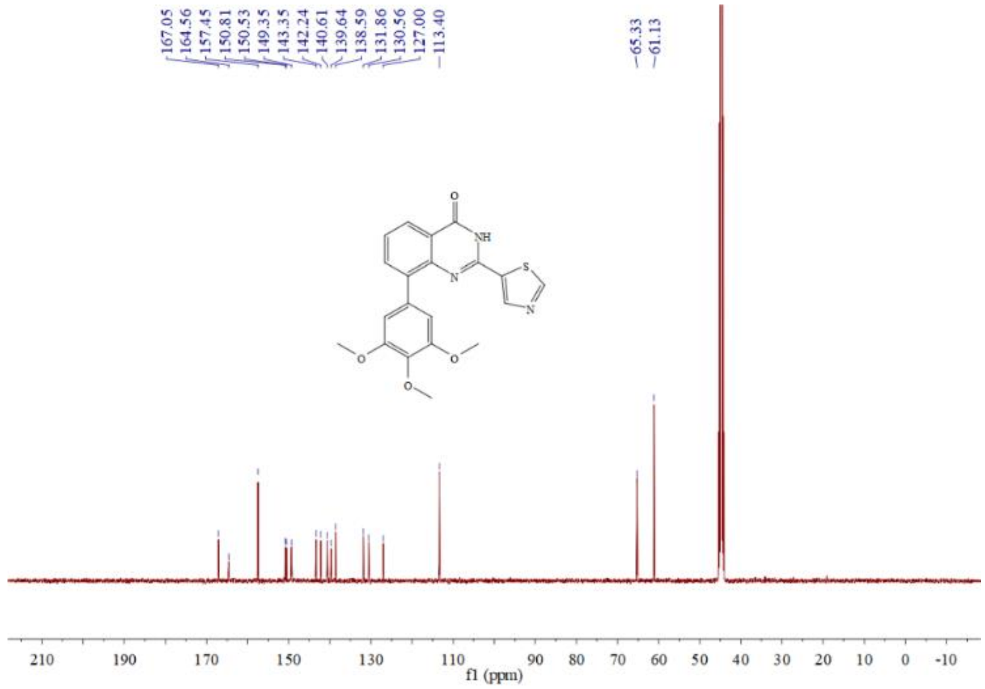

Figure S76: ^{13}C NMR spectrum of A18.

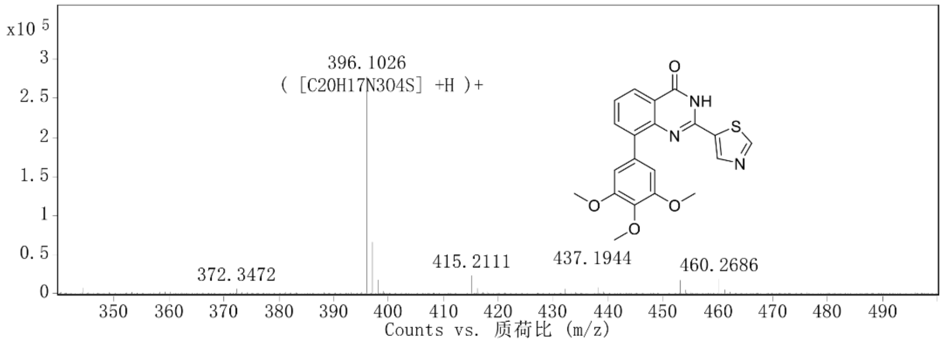

Figure S77: HR MS spectrum of A18.

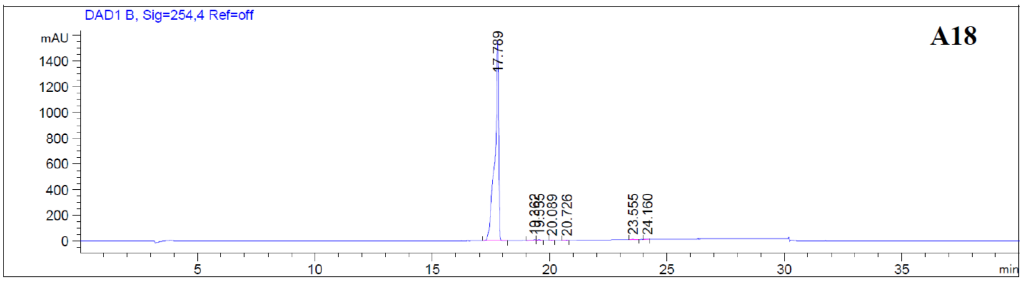

Signal 2: DAD1 B, Sig=254,4 Ref=off

Peak #	Retention time [min]	Type	Peak Width [min]	Peak area [mAU*s]	Peak height [mAU]	Peak area %
1	17. 789	BV R	0. 1538	1. 76219e4	1559. 84937	98. 9463
2	19. 362	BV	0. 1489	71. 06097	6. 97302	0. 3990
3	19. 555	VB	0. 1072	66. 62617	9. 12169	0. 3741
4	20. 089	VB	0. 1012	16. 01887	2. 30045	0. 0899
5	20. 726	BV	0. 1113	10. 09391	1. 23509	0. 0567
6	23. 555	BV R	0. 1257	15. 16284	1. 55581	0. 0851
7	24. 160	BB	0. 0941	8. 69962	1. 26758	0. 0488
Total amount:				1. 78095e4	1582. 30301	

Figure S78: HPLC spectrum of A18.

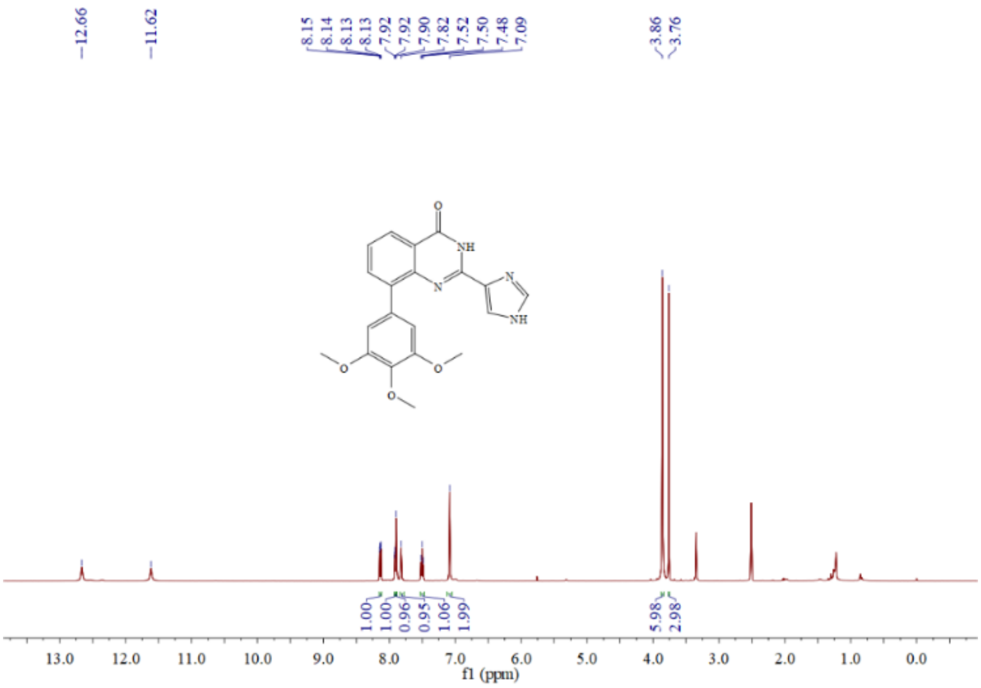

Figure S79: ^{1}H NMR spectrum of A19.

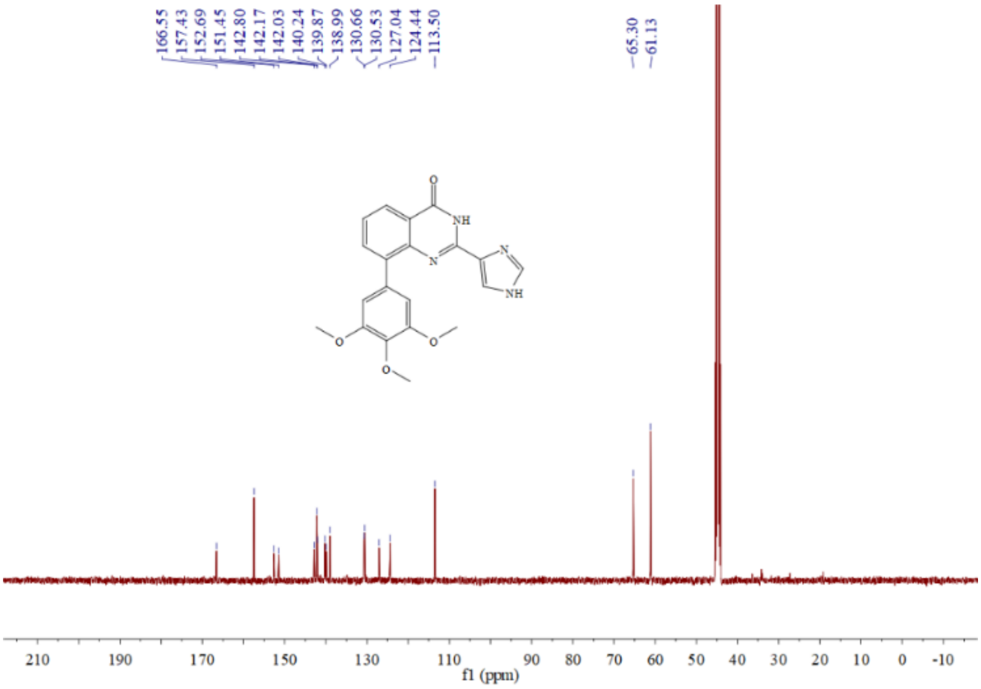

Figure S80: ^{13}C NMR spectrum of A19.

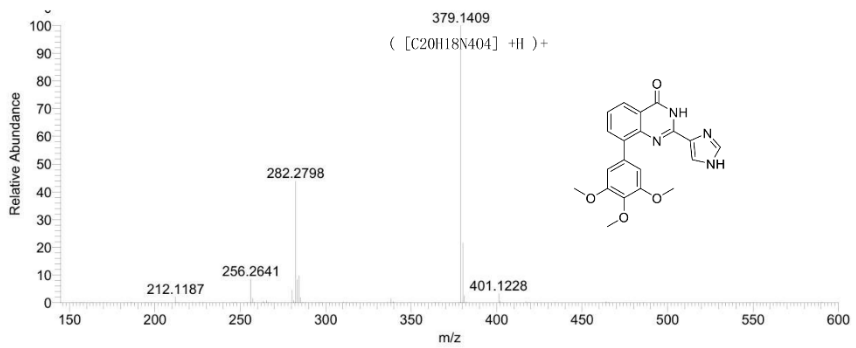

Figure S81: HR MS spectrum of A19.

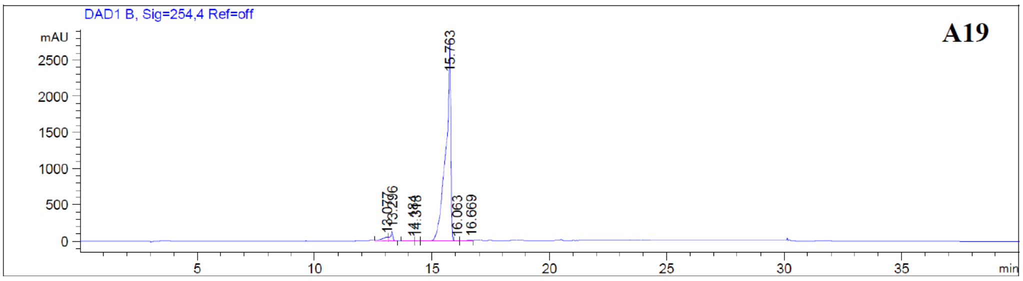

Signal 2: DAD1 B, Sig=254,4 Ref=off

Peak #	Retention time [min]	Type	Peak Width [min]	Peak area [mAU*s]	Peak height [mAU]	Peak area %
1	13.077	VV	0.2763	1103.32727	51.84291	2.4990
2	13.296	VB	0.1043	969.35077	131.10399	2.1955
3	14.184	BV	0.1927	49.41853	3.30001	0.1119
4	14.318	VB	0.0807	16.36403	2.94915	0.0371
5	15.763	BV R	0.2023	4.19578e4	2776.31763	95.0327
6	16.063	VB E	0.0938	8.53443	1.31400	0.0193
7	16.669	BB	0.1731	46.12991	3.47013	0.1045
Total amount:				4.41509e4	2970.29781	

Figure S82: HPLC spectrum of A19.

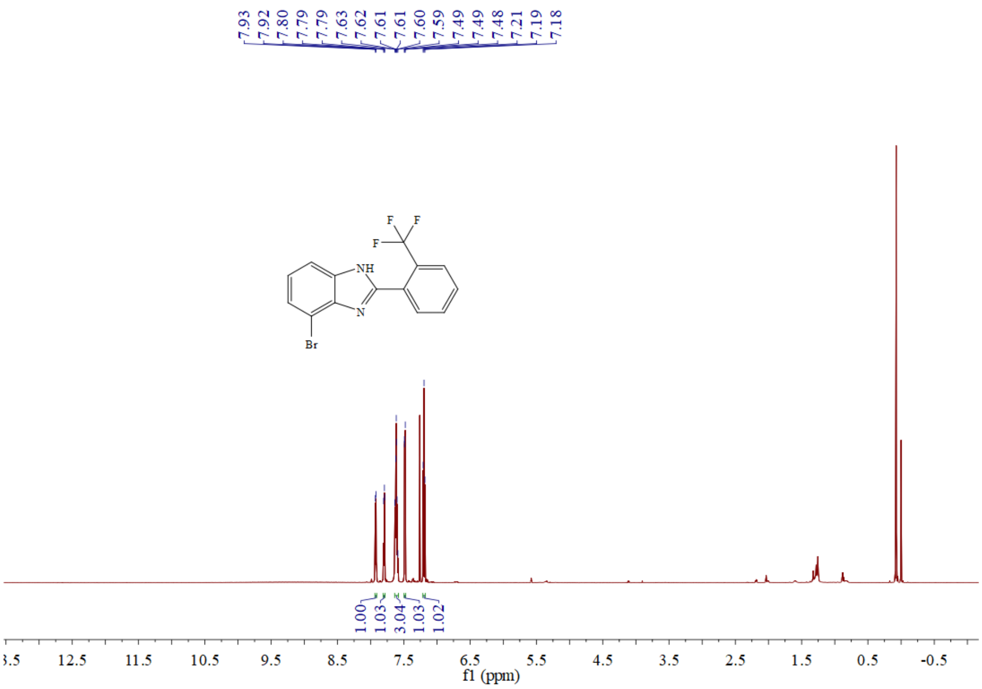

Figure S83: ^{1}H NMR spectrum of b0.

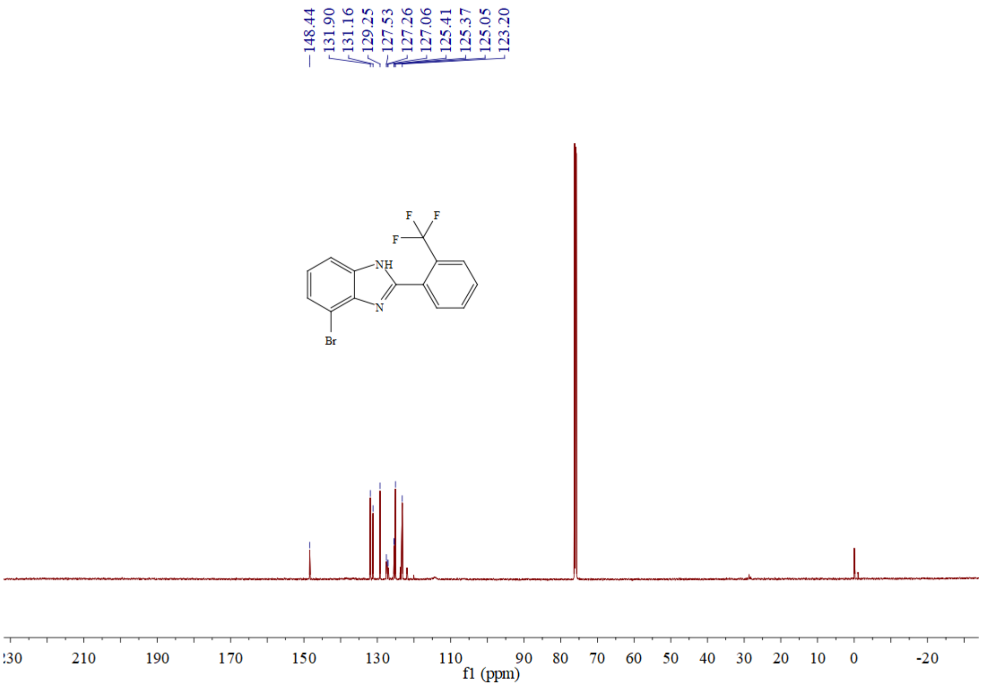

Figure S84: ^{13}C NMR spectrum of b0.

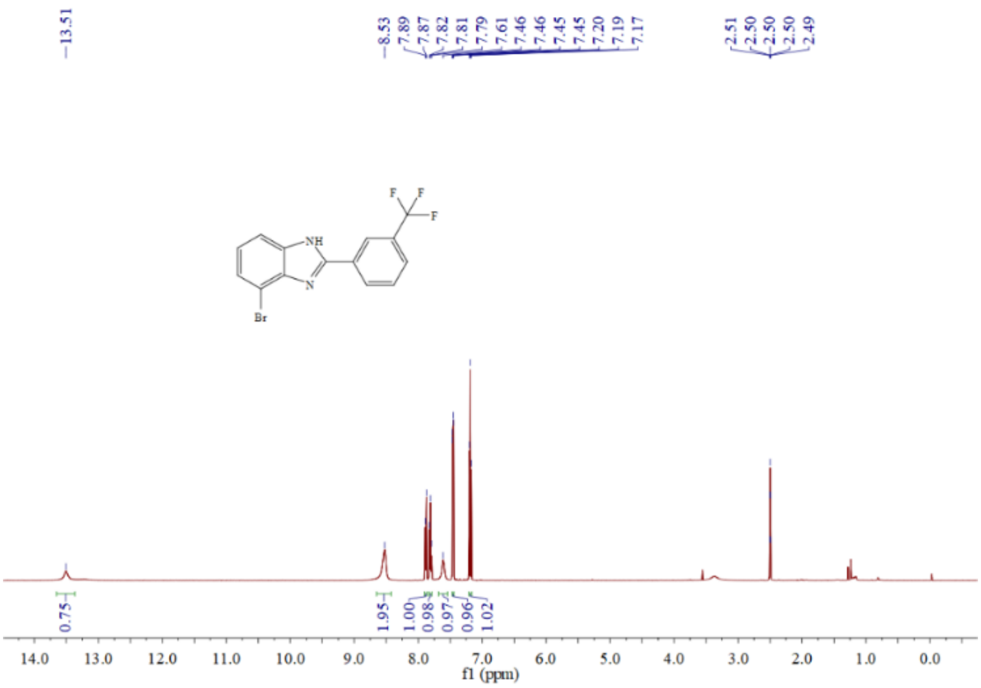

Figure S85: ¹H NMR spectrum of b1.

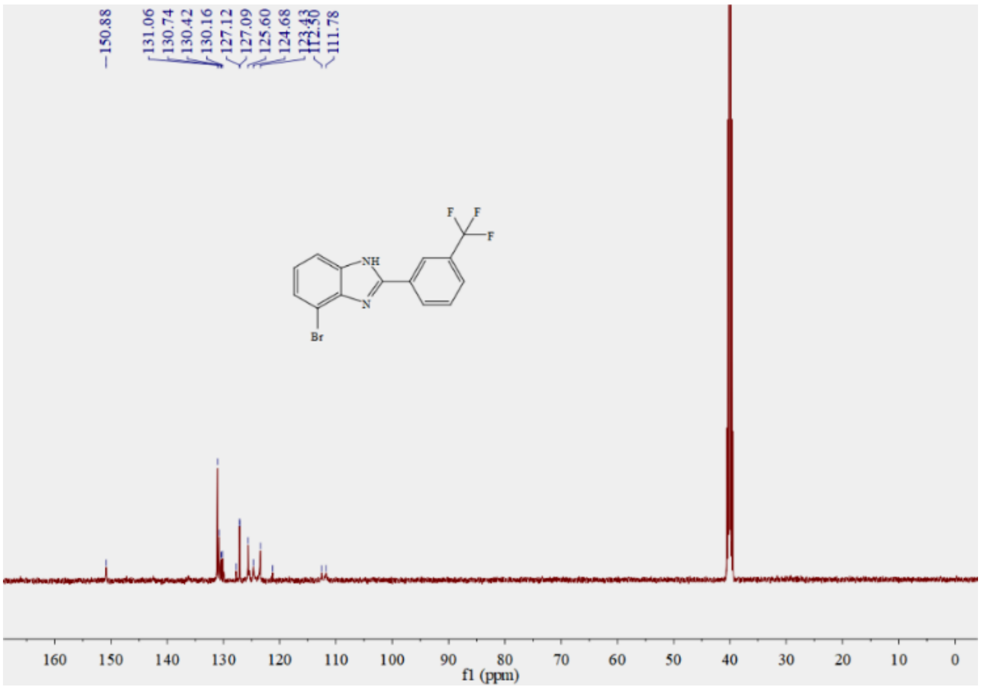

Figure S86: ¹³C NMR spectrum of b1.

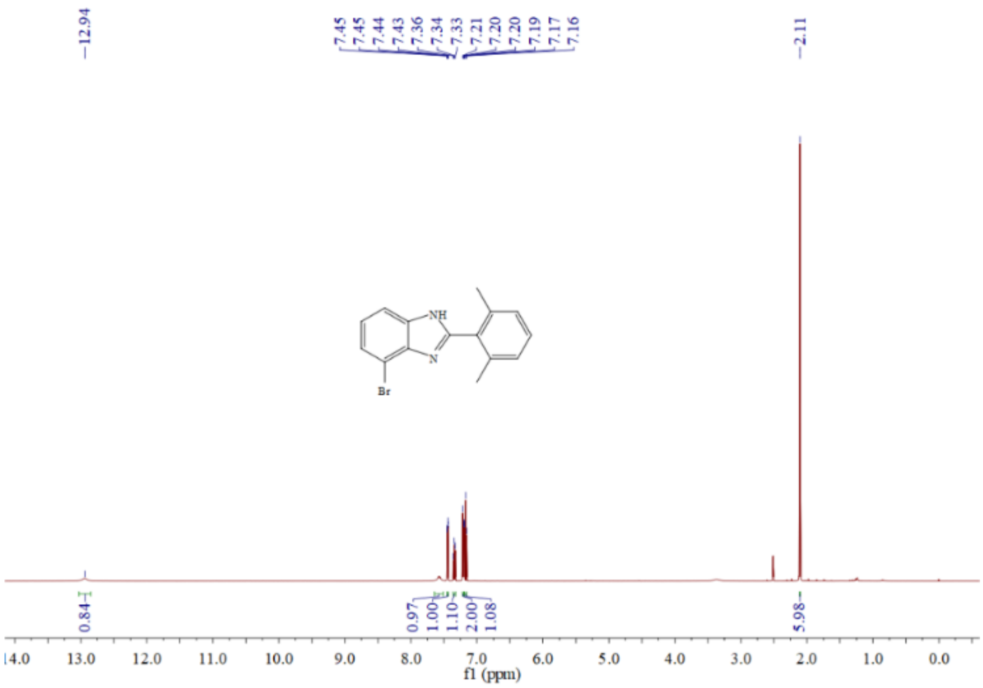

Figure S87: ^{1}H NMR spectrum of b2.

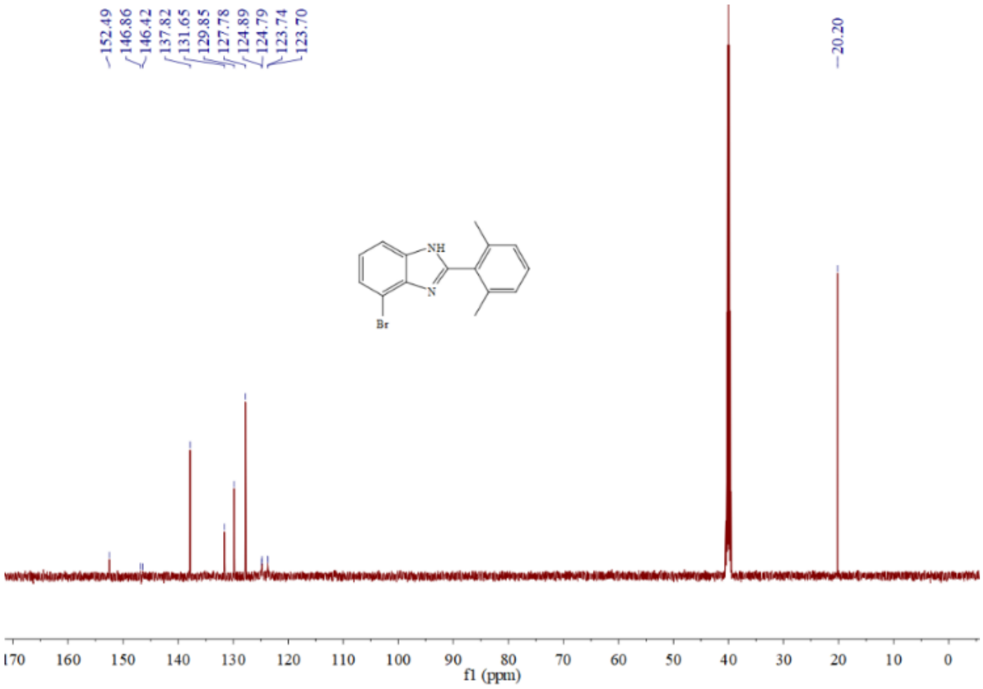

Figure S88: ^{13}C NMR spectrum of b2.

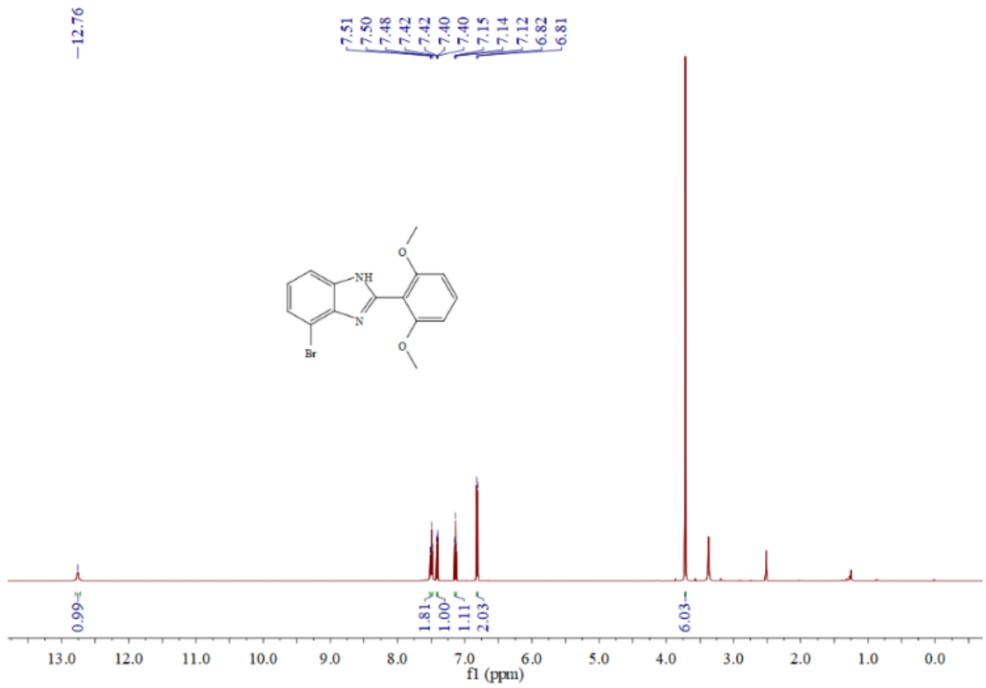

Figure S89: ^{1}H NMR spectrum of b3.

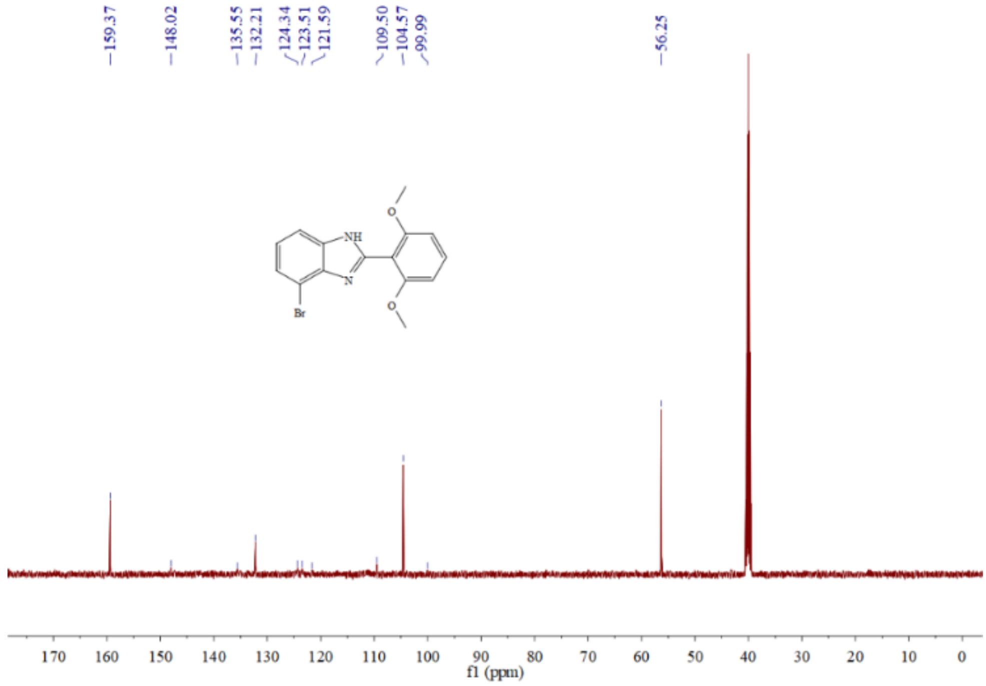

Figure S90: ^{13}C NMR spectrum of b3.

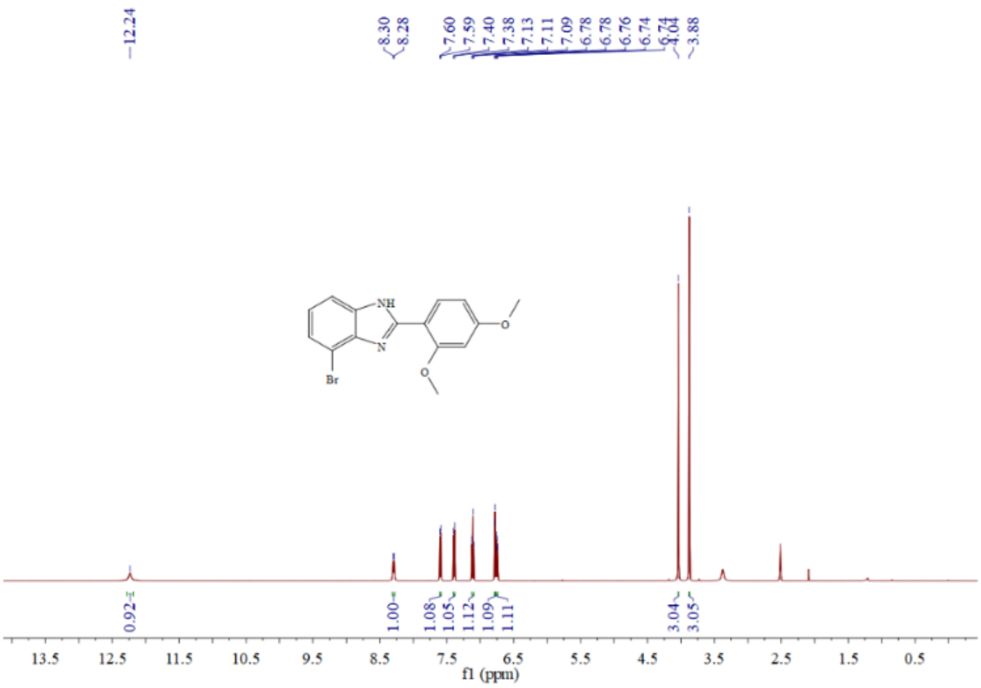

Figure S91: ^{1}H NMR spectrum of b4.

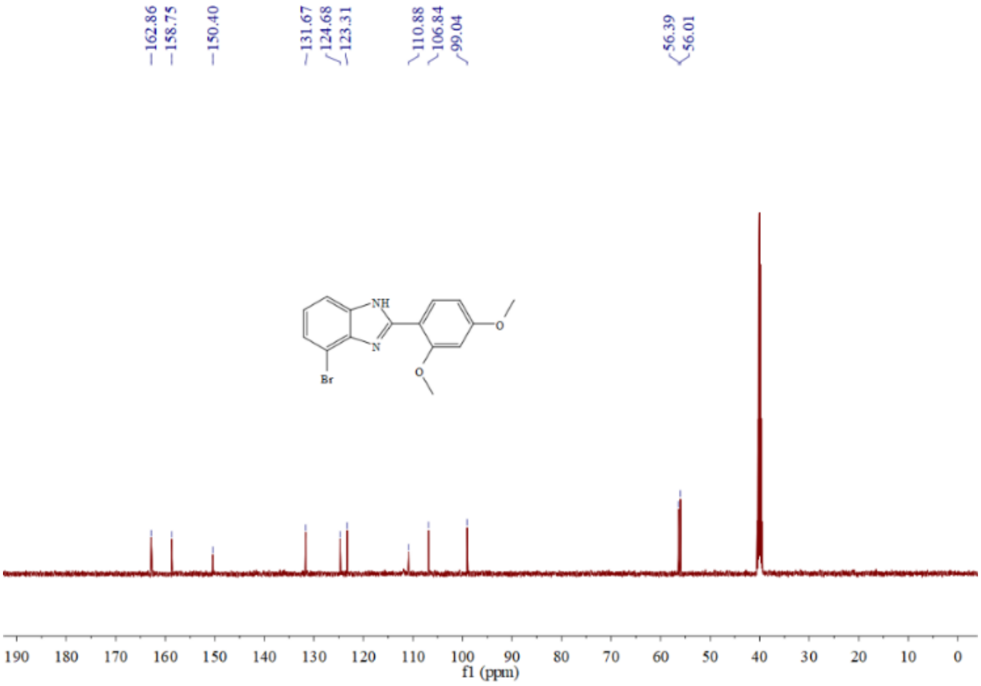

Figure S92: ^{13}C NMR spectrum of b4.

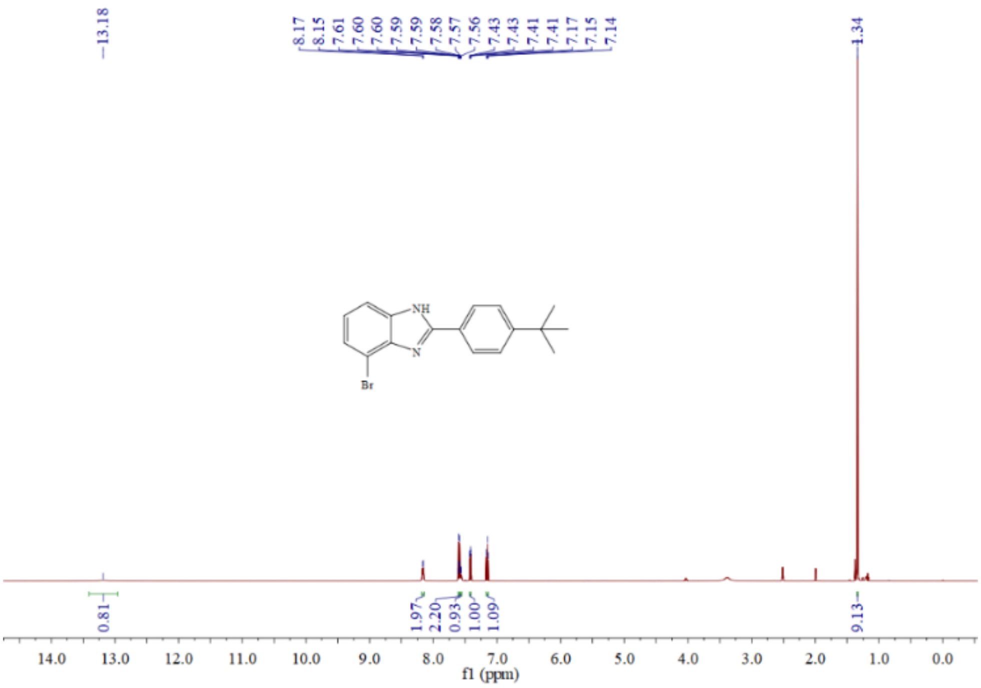

Figure S93: ^{1}H NMR spectrum of b5.

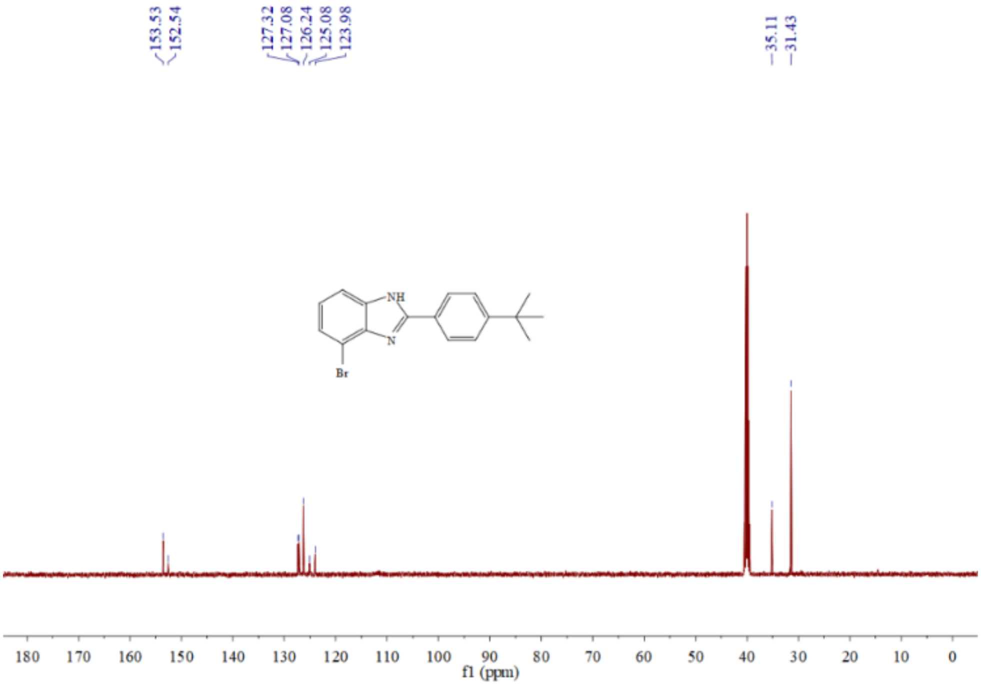

Figure S94: ^{13}C NMR spectrum of b5.

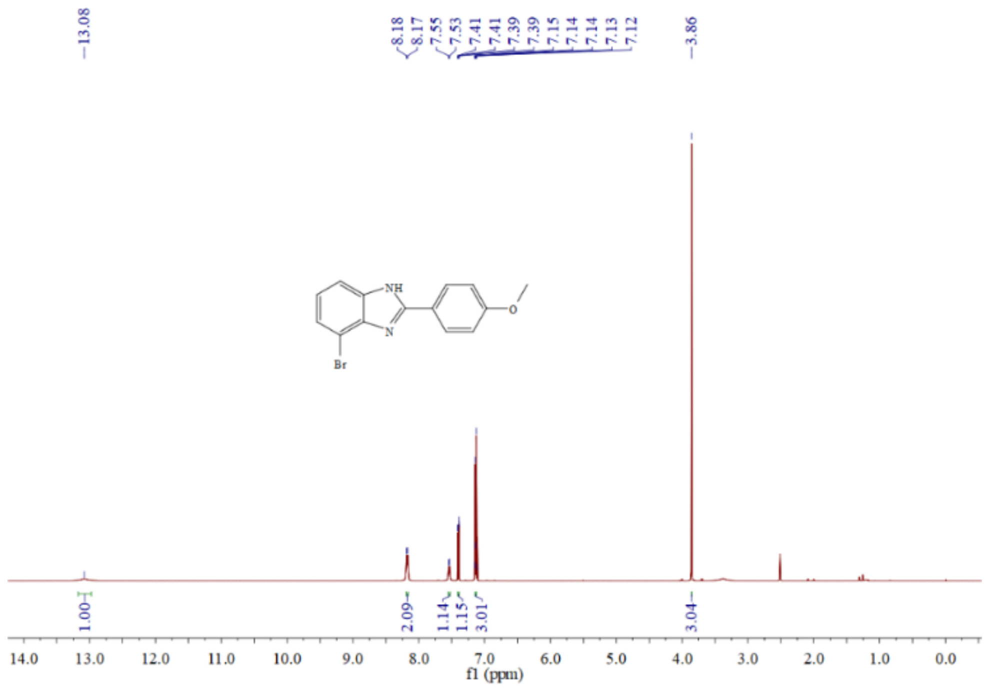

Figure S95: ^{1}H NMR spectrum of b6.

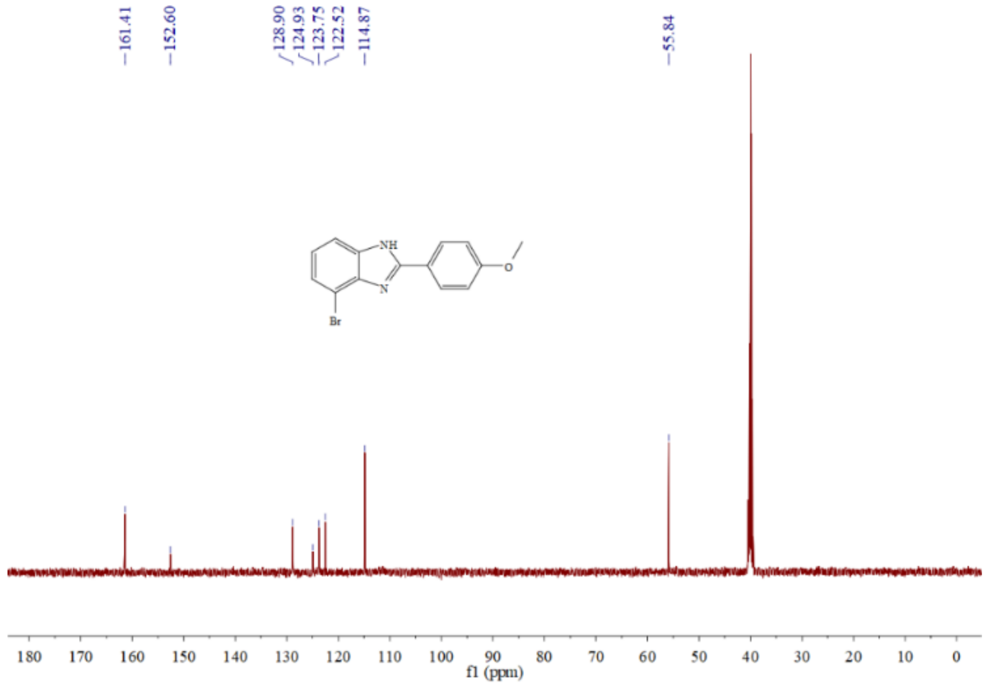

Figure S96: ^{13}C NMR spectrum of b6.

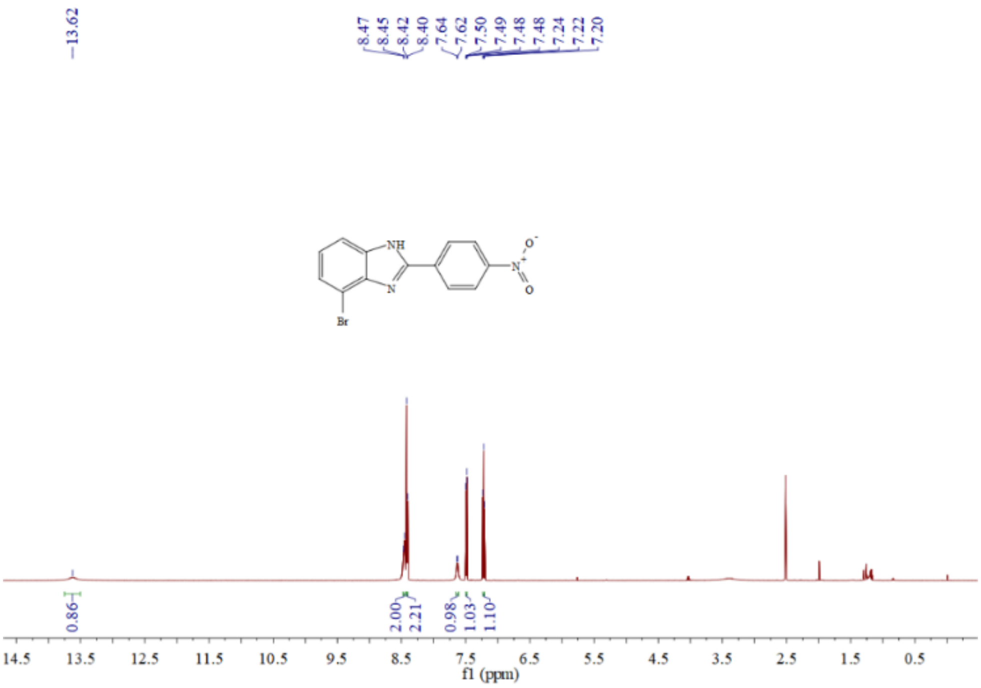

Figure S97: ^{1}H NMR spectrum of b7.

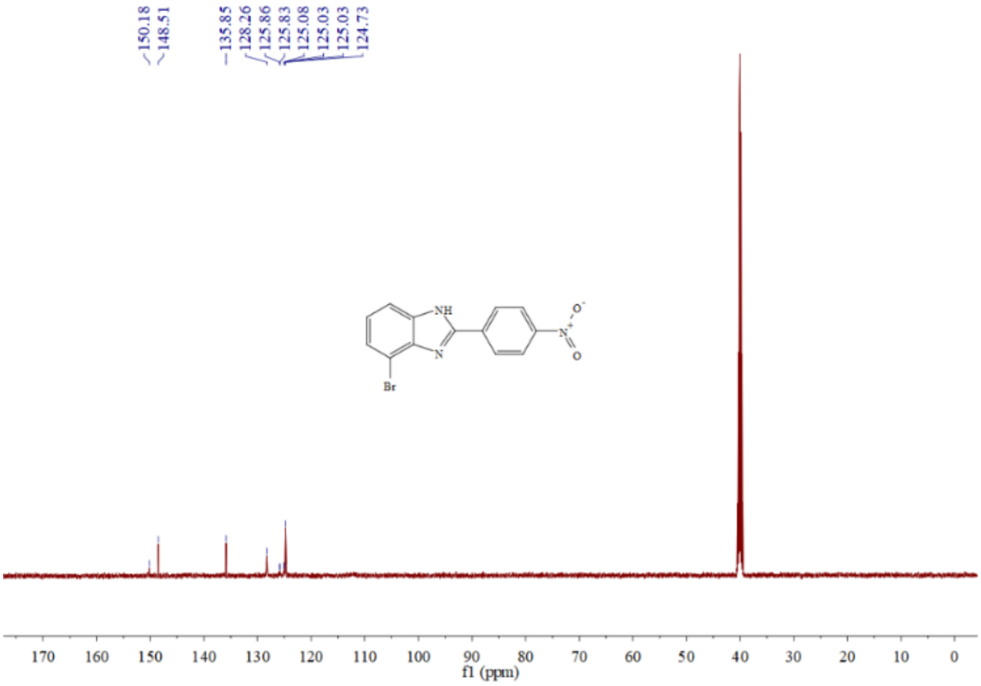

Figure S98: ^{13}C NMR spectrum of b7.

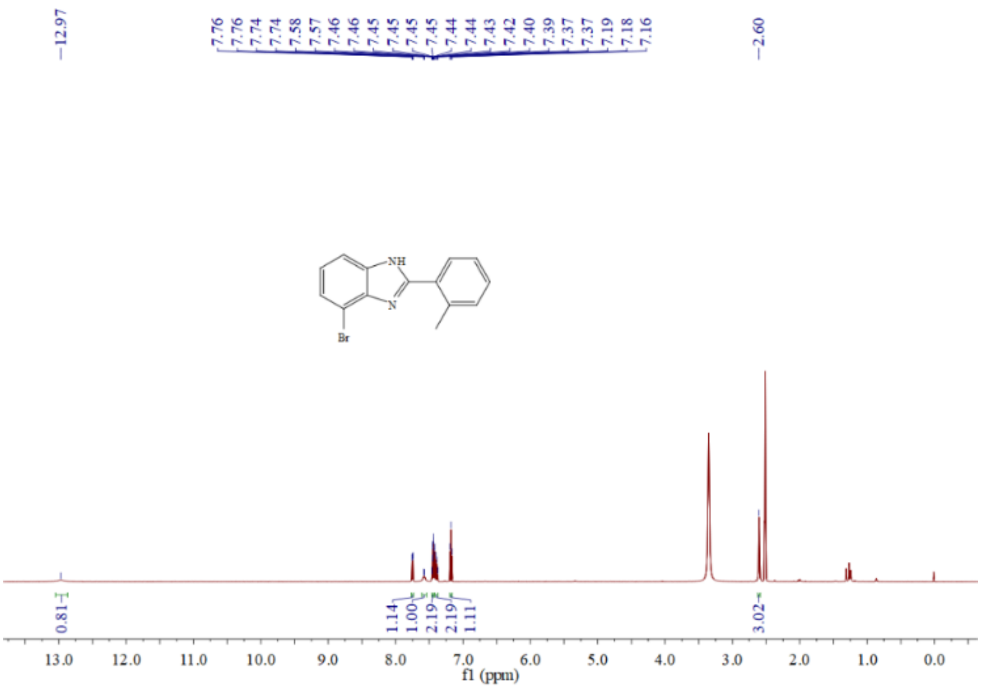

Figure S99: ^{1}H NMR spectrum of b8.

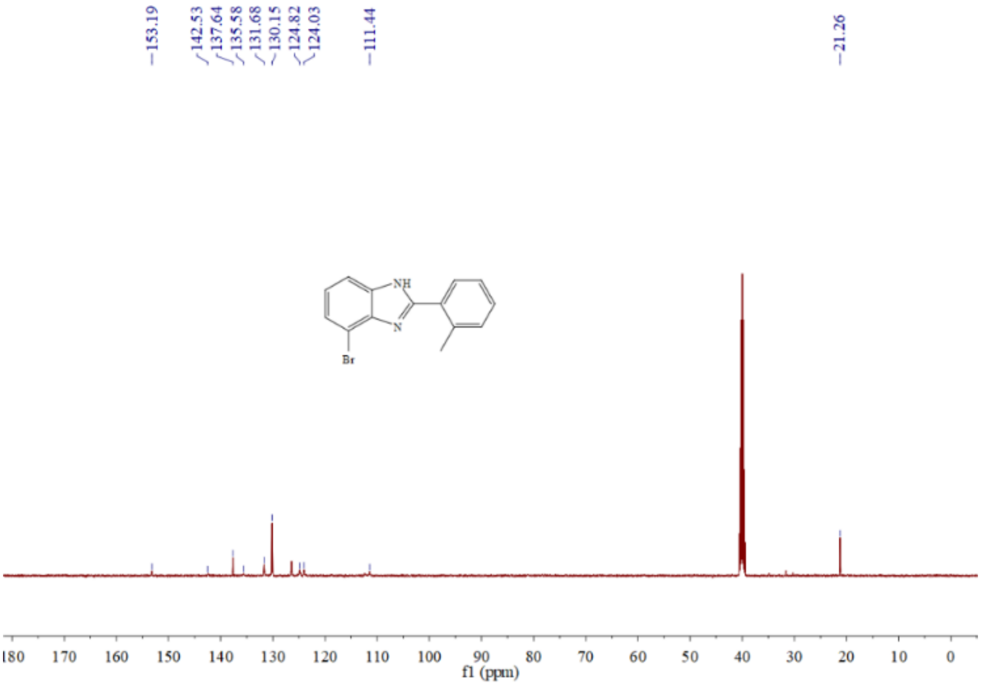

Figure S100: ^{13}C NMR spectrum of b8.

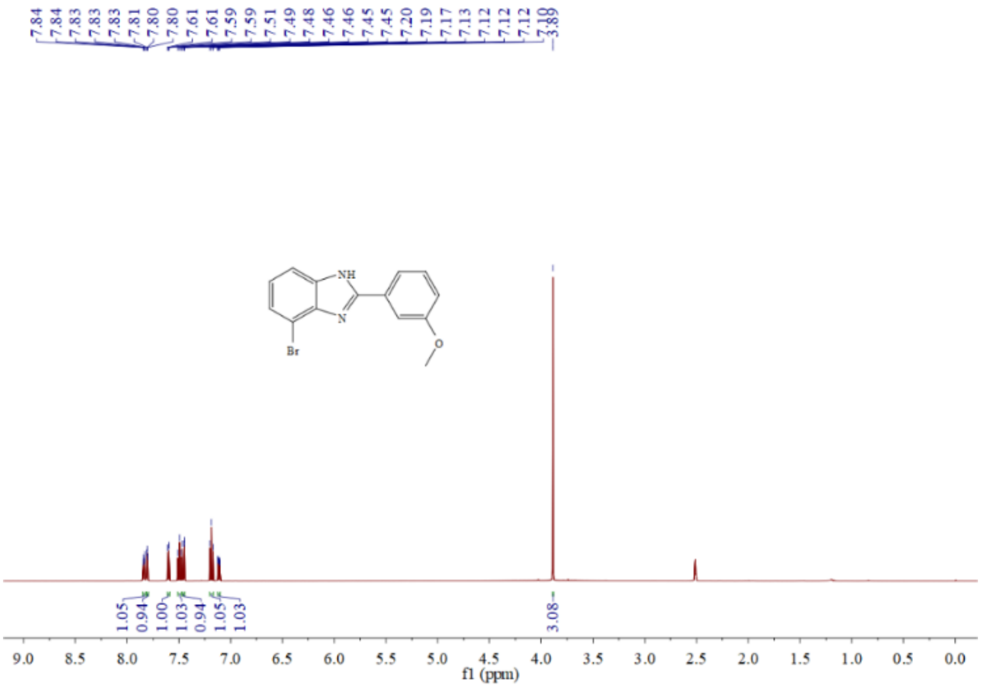

Figure S101: ^{1}H NMR spectrum of b9.

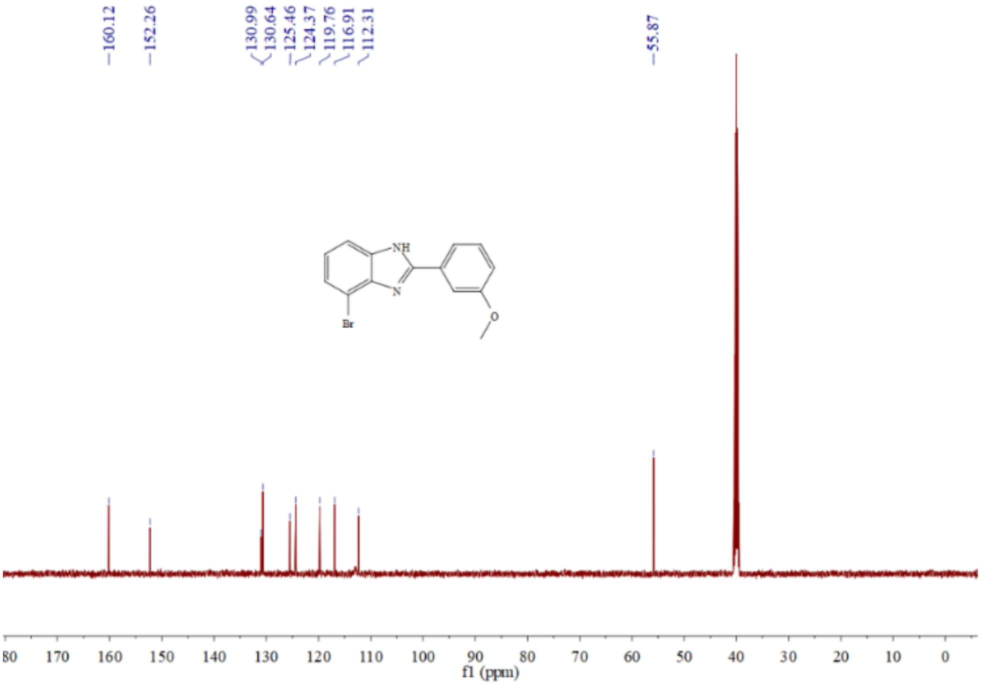

Figure S102: ^{13}C NMR spectrum of b9.

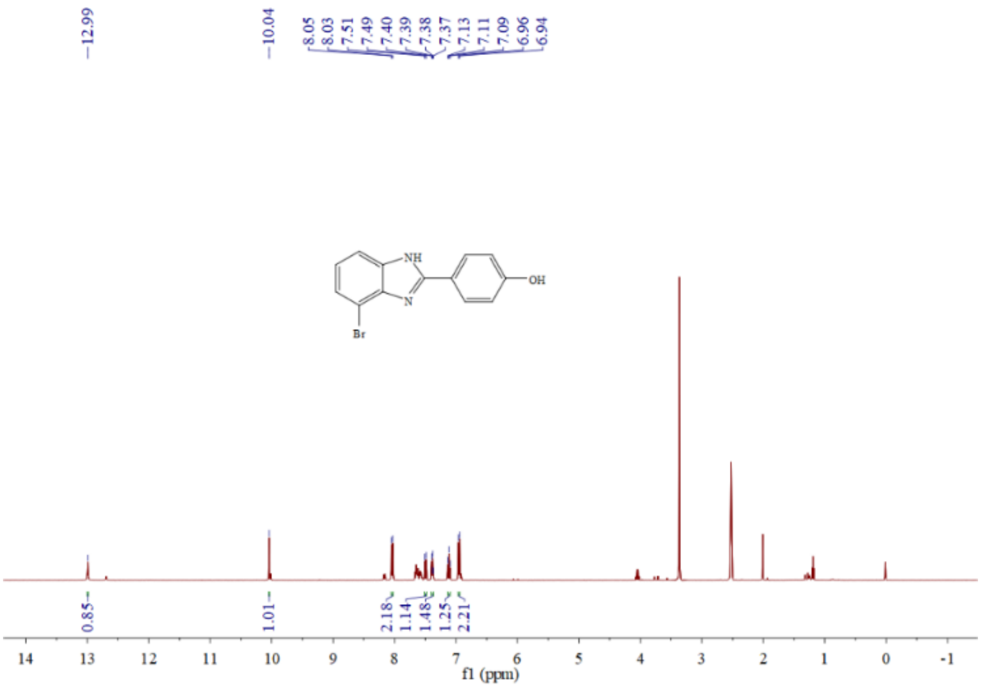

Figure S103: ^{1}H NMR spectrum of b10.

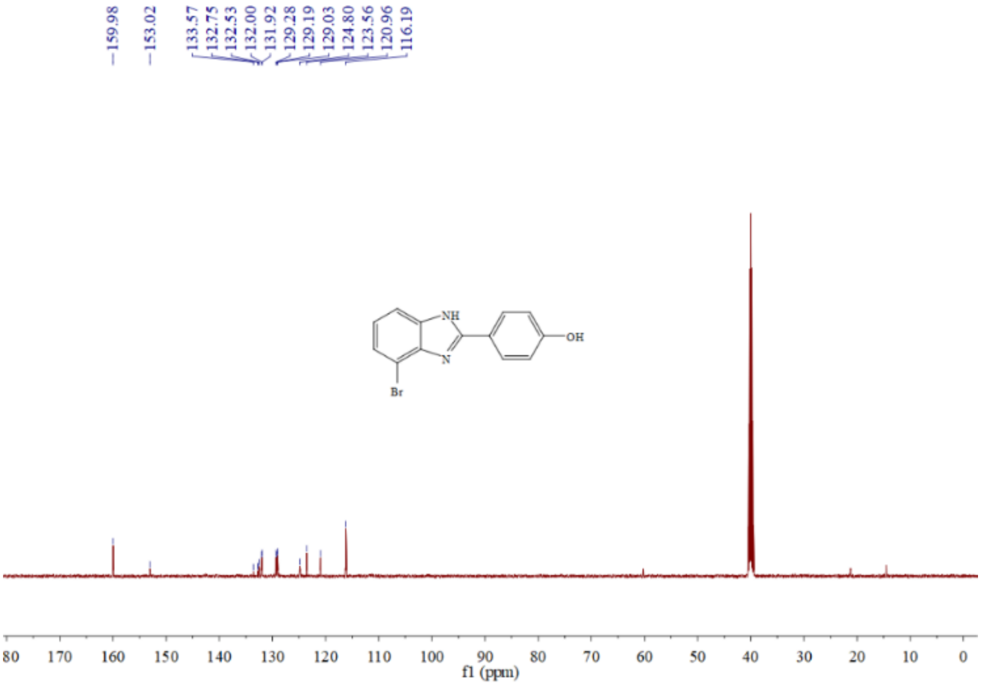

Figure S104: ^{13}C NMR spectrum of b10.

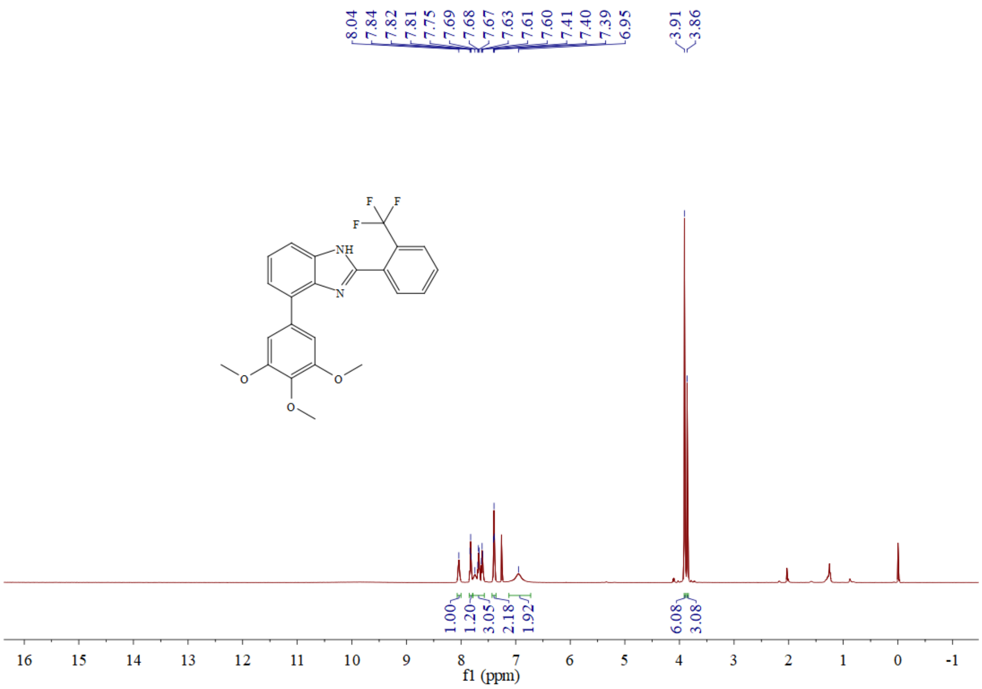

Figure S105: ^{1}H NMR spectrum of B0.

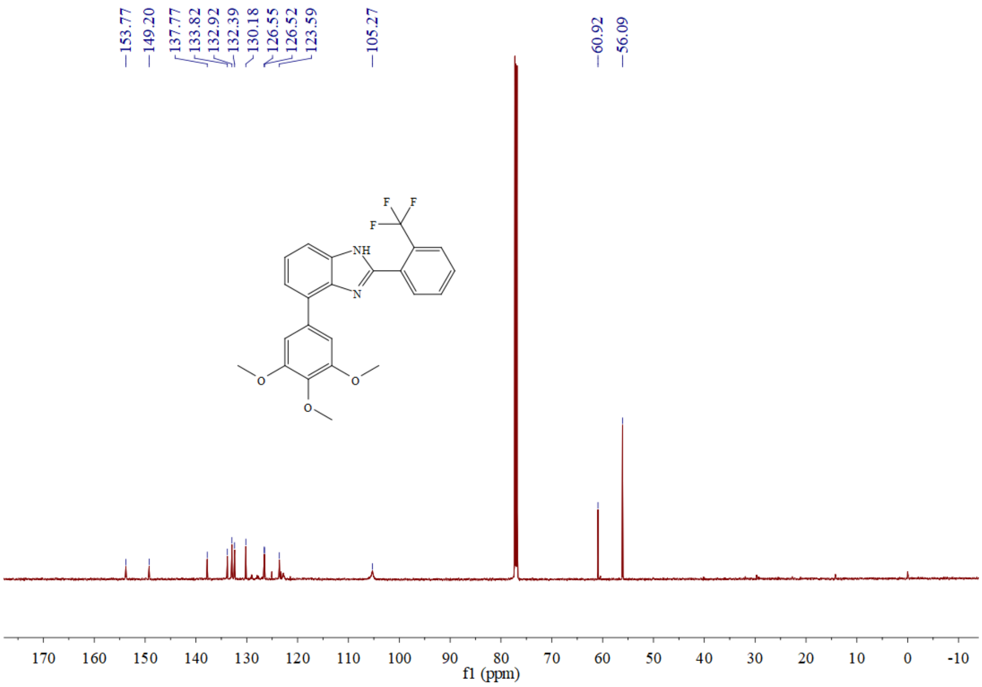

Figure S106: ^{13}C NMR spectrum of B0.

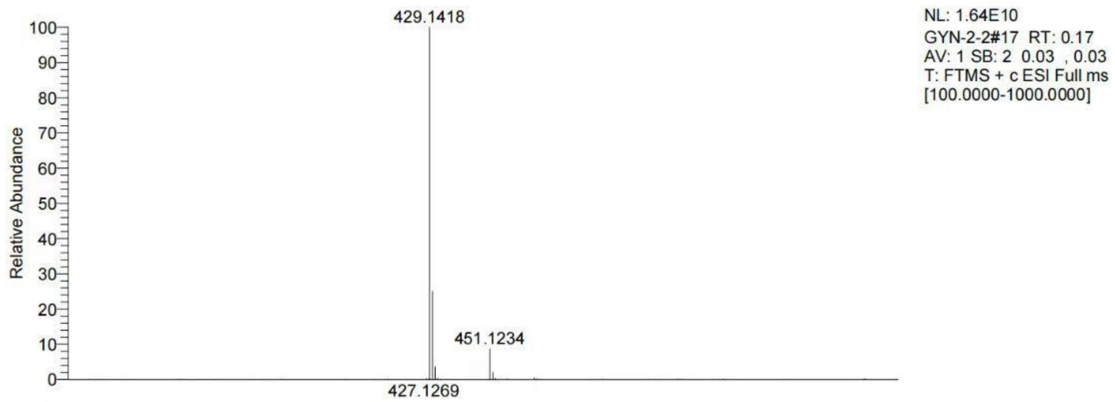

Figure S107: HR MS spectrum of B0.

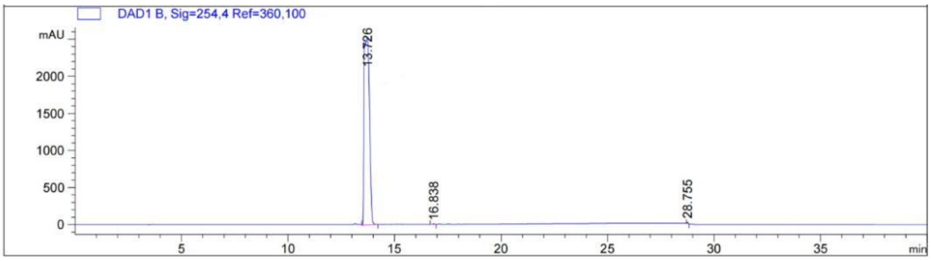

Signal 2: DAD1 B, Sig=254,4 Ref=360,100

Peak #	Retention time [min]	Type	Peak Width [min]	Peak area [mAU*s]	Peak Height [mAU]	Peak area %
1	13.726	MM	0.2924	4.44611e4	2533.90283	99.7557
2	16.838	BV	0.0819	40.99683	7.72969	0.0920
3	28.755	BB	0.0491	67.86900	22.52678	0.1523
Total amount :				4.45699e4	2564.15929	

Figure S108: HPLC spectrum of B0.

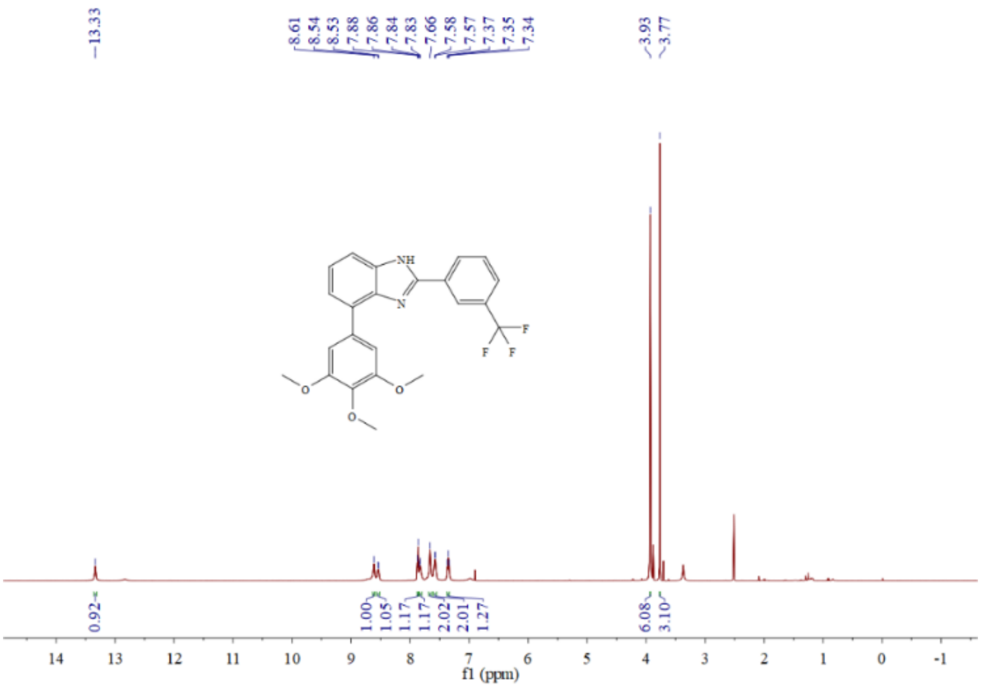

Figure S109: ¹H NMR spectrum of B1.

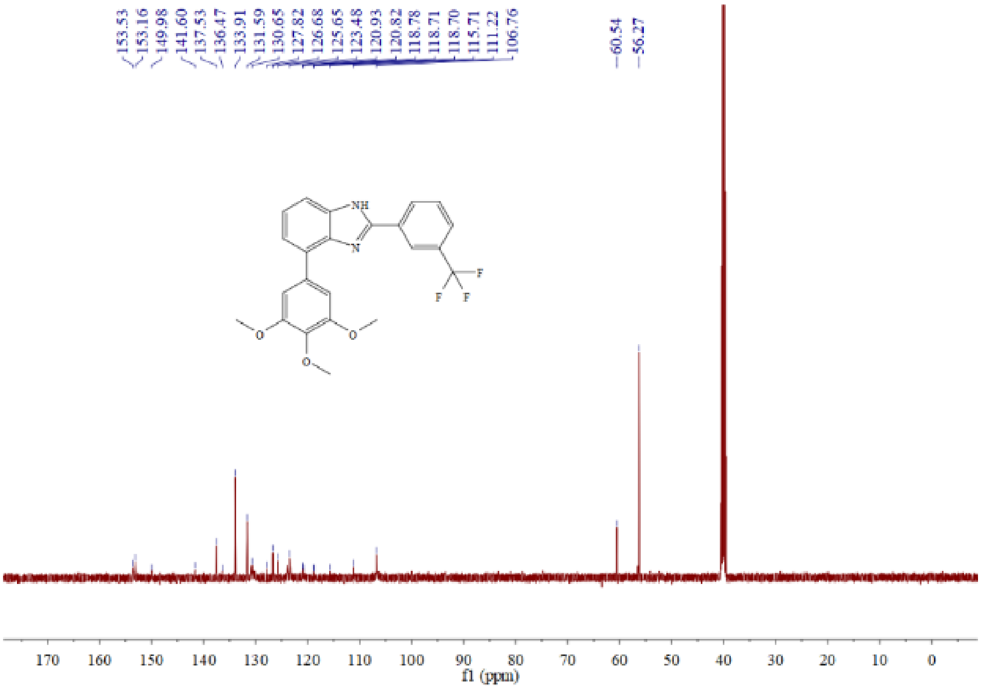

Figure S110: ¹³C NMR spectrum of B1.

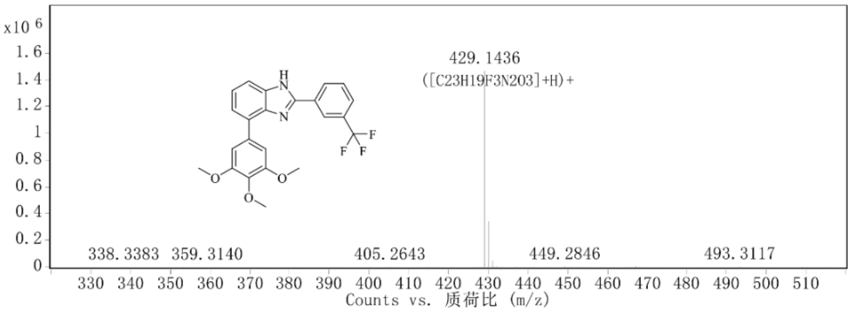

Figure S111: HR MS spectrum of B1.

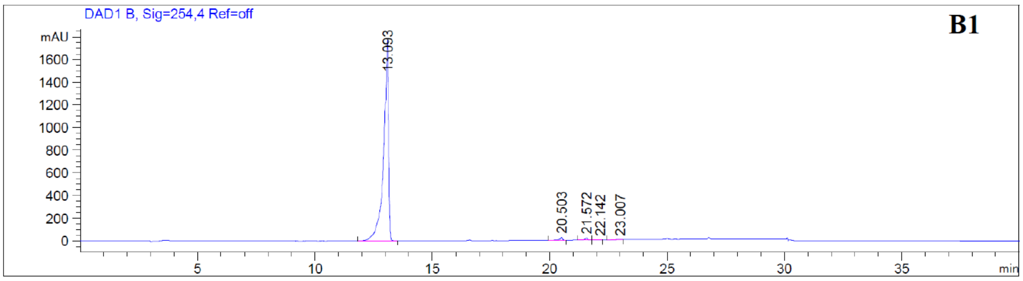

Signal 2: DAD1 B, Sig=254,4 Ref=off

Peak #	Retention time [min]	Type	Peak Width [min]	Peak area [mAU*s]	Peak height [mAU]	Peak area %
1	13. 093	BB	0. 1922	2. 59488e4	1778. 57605	98. 4215
2	20. 503	BB	0. 1192	226. 20305	26. 60421	0. 8580
3	21. 572	BB	0. 1120	148. 37042	18. 81226	0. 5628
4	22. 142	BB	0. 0957	10. 06286	1. 47311	0. 0382
5	23. 007	BV	0. 1590	31. 53255	2. 60968	0. 1196

Total amount: 2. 63650e4 1828. 07530

Figure S112: HPLC spectrum of B1.

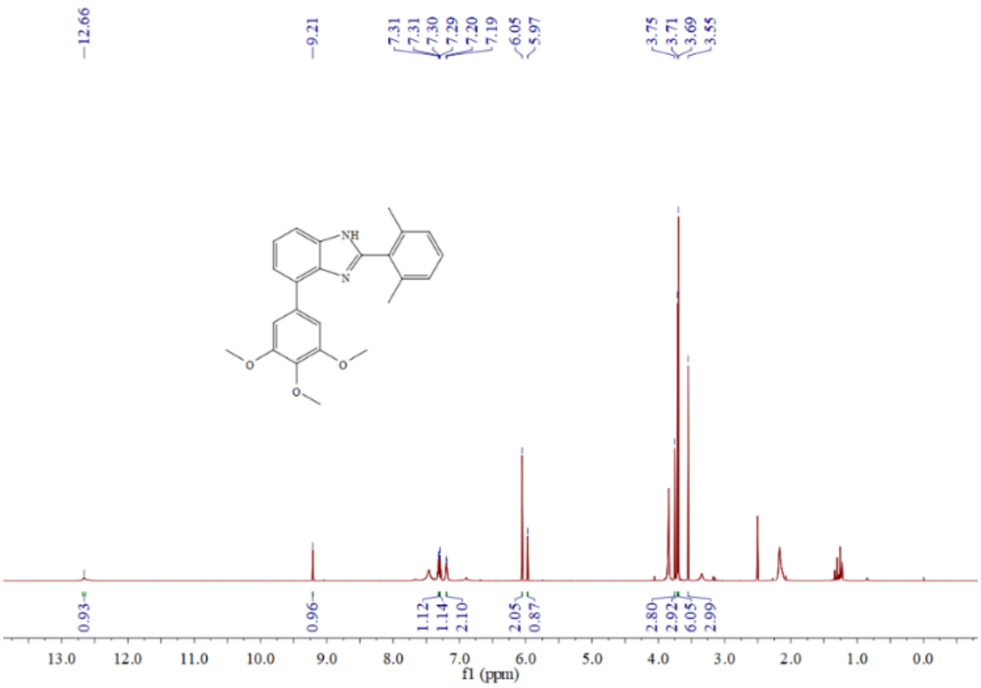

Figure S113: ^{1}H NMR spectrum of B2.

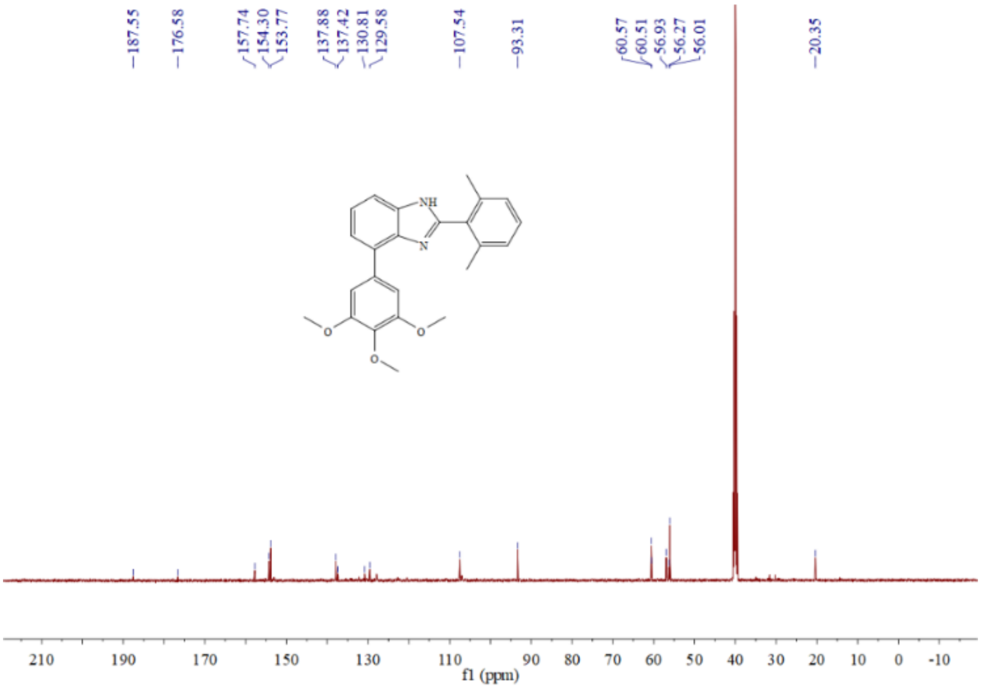

Figure S114: ^{13}C NMR spectrum of B2.

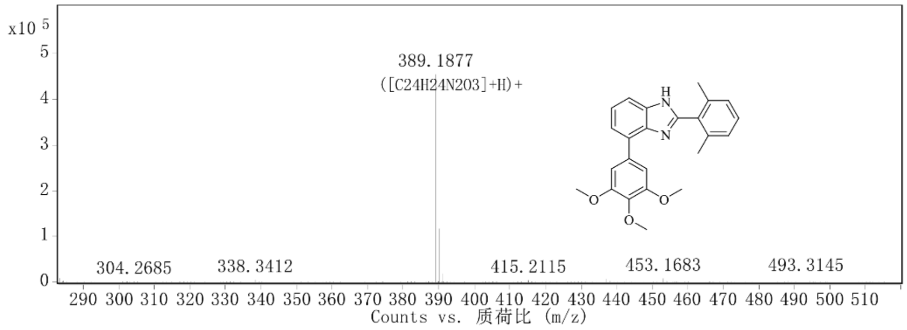

Figure S115: HR MS spectrum of B2.

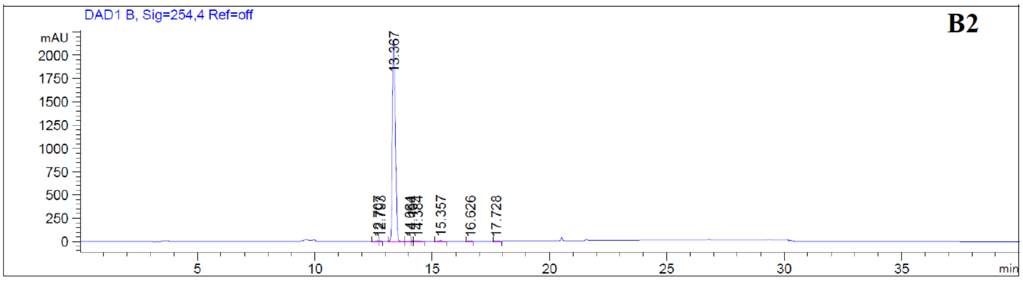

Signal 2: DAD1 B, Sig=254,4 Ref=off

Peak #	Retention time [min]	Type	Peak Width [min]	Peak area [mAU*s]	Peak height [mAU]	Peak area %
1	12.707	BV	0.1007	39.54552	5.71396	0.2020
2	12.793	VB	0.0767	36.83859	7.07673	0.1881
3	13.367	BB	0.1443	1.93303e4	2163.18506	98.7218
4	14.064	BV	0.1040	8.68609	1.12556	0.0444
5	14.154	VV	0.0670	7.73516	1.63978	0.0395
6	14.384	VB	0.1929	49.00500	3.26895	0.2503
7	15.357	BB	0.1230	64.31408	7.28140	0.3285
8	16.626	BB	0.1290	28.63547	3.67069	0.1462
9	17.728	BB	0.1123	15.52458	2.05174	0.0793
Total amount:				1.95806e4	2195.01386	

Figure S116: HPLC spectrum of B2.

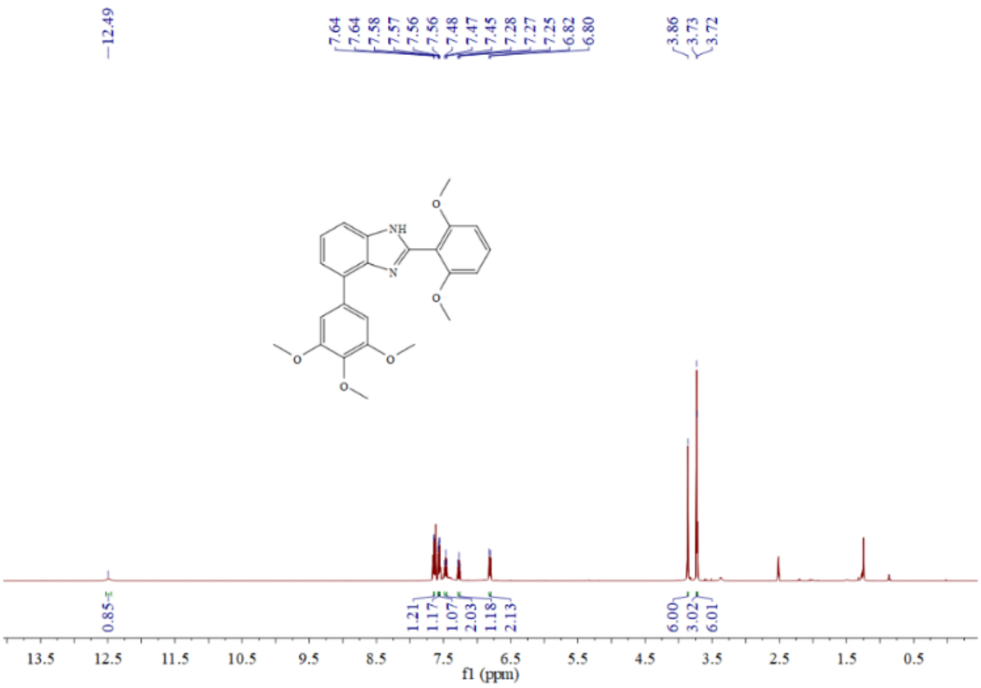

Figure S117: ^{1}H NMR spectrum of B3.

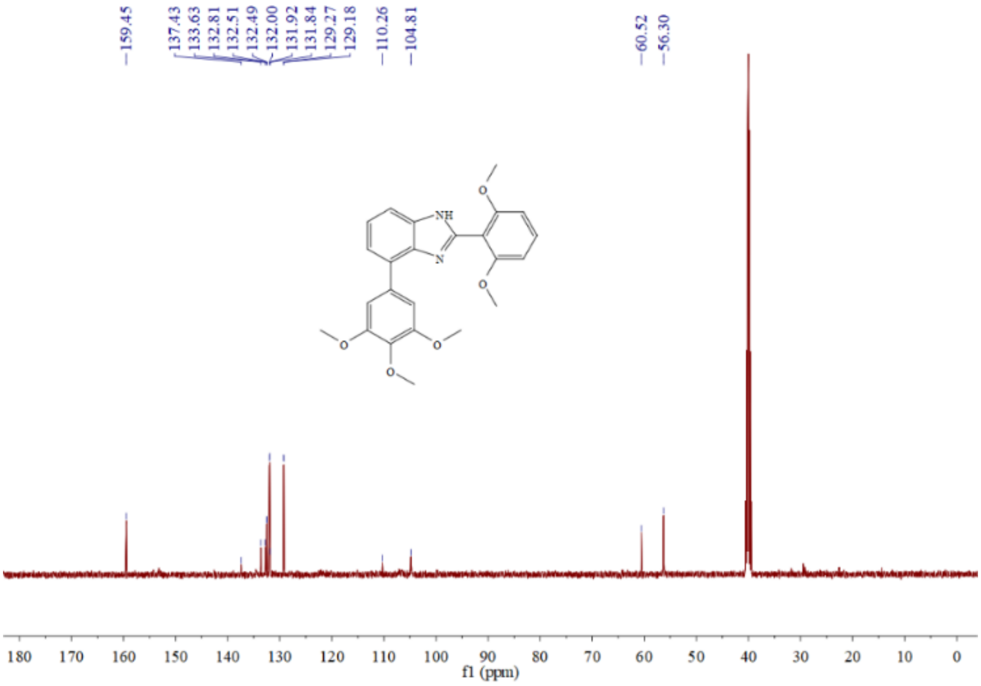

Figure S118: ^{13}C NMR spectrum of B3.

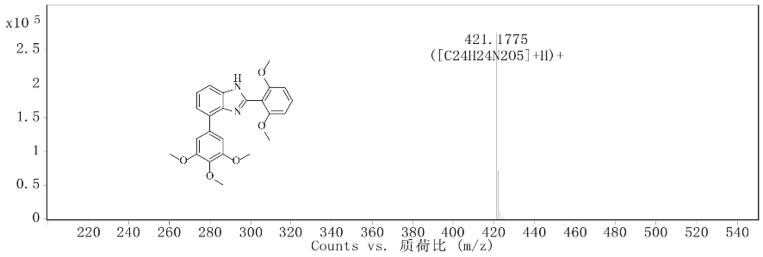

Figure S119: HR MS spectrum of B3.

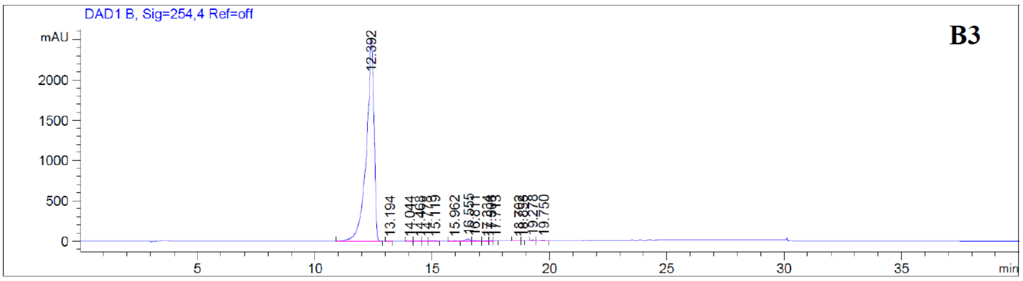

Signal 2: DAD1 B. Sig=254,4 Ref=off

Peak #	Retention time [min]	Type	Peak Width [min]	Peak area [mAU*s]	Peak height [mAU]	Peak area %
1	12.392	BB	0.3005	5.66753e4	2500.04150	98.6207
2	13.194	BB	0.1113	13.25760	1.69492	0.0231
3	14.044	BB	0.1214	11.85341	1.36407	0.0206
4	14.468	BV	0.1391	19.96629	1.92333	0.0347
5	14.778	VV	0.1442	24.77883	2.22095	0.0431
6	15.119	VB	0.2034	89.84007	5.90759	0.1563
7	15.962	BV E	0.1604	25.49608	2.18078	0.0444
8	16.555	VV R	0.1864	315.32748	22.64661	0.5487
9	16.811	VB	0.1741	85.16678	6.36439	0.1482
10	17.334	BV	0.1250	22.86505	2.64195	0.0398
11	17.506	VV	0.1023	16.44570	2.38880	0.0286
12	17.713	VV	0.1146	9.05603	1.19244	0.0158
13	18.702	BV	0.1028	24.22023	3.49513	0.0421
14	18.858	VB	0.0795	7.17657	1.36092	0.0125
15	19.278	BV	0.0966	68.25343	10.96569	0.1188
16	19.750	VB	0.1597	58.94163	4.92139	0.1026
Total amount:				5.74680e4	2571.31046	

Figure S120: HPLC spectrum of B3.

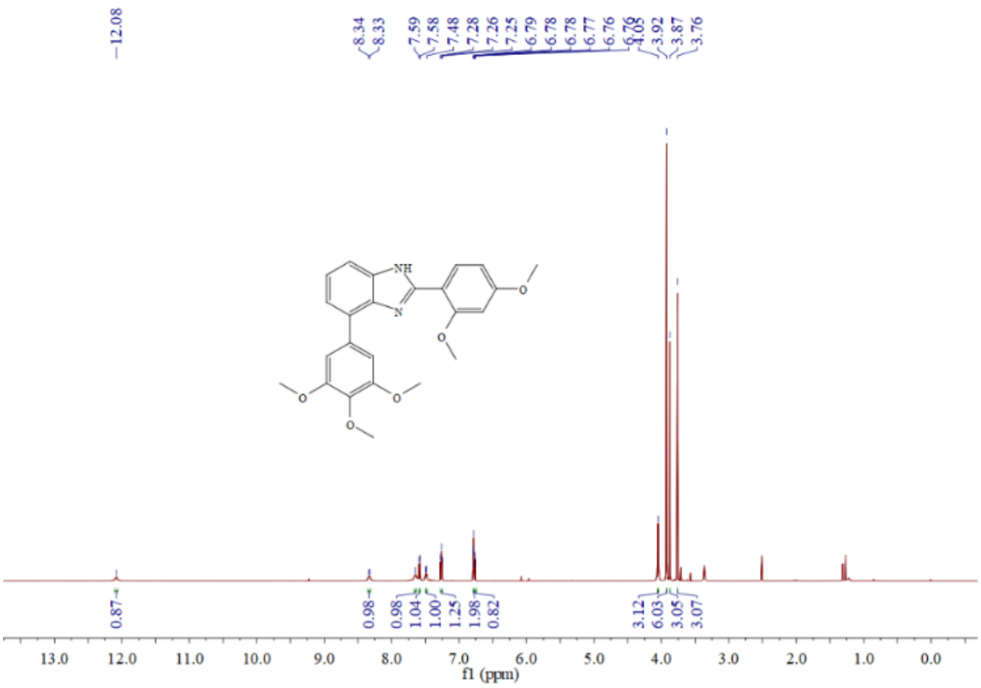

Figure S121: ^{1}H NMR spectrum of B4.

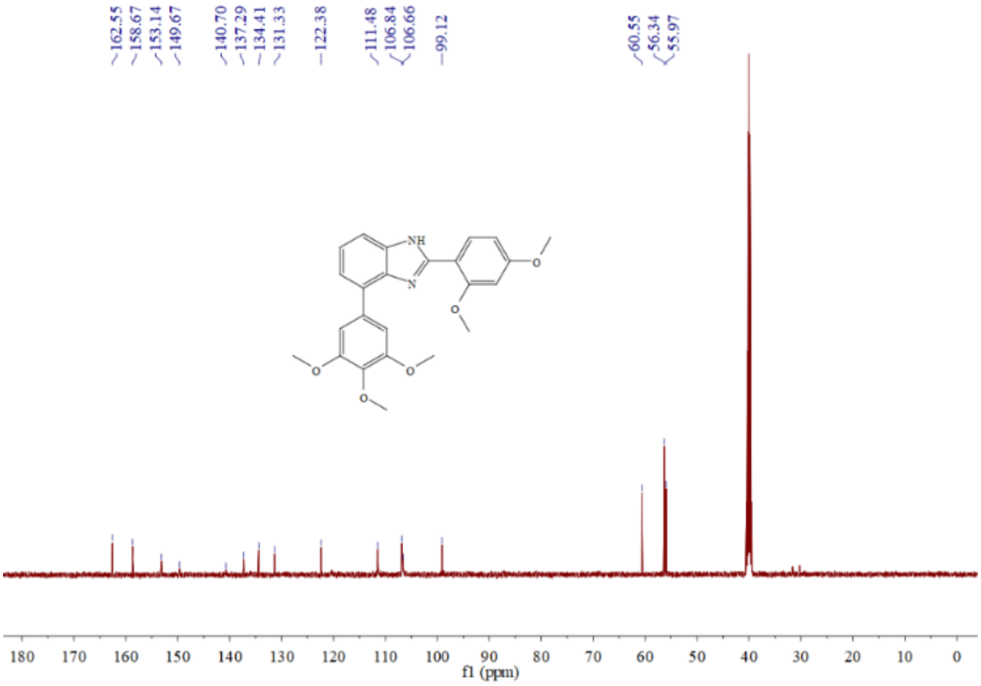

Figure S122: ^{13}C NMR spectrum of B4.

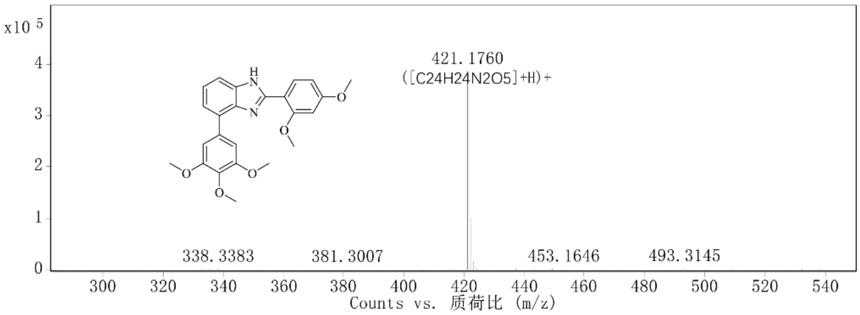

Figure S123: HR MS spectrum of B4.

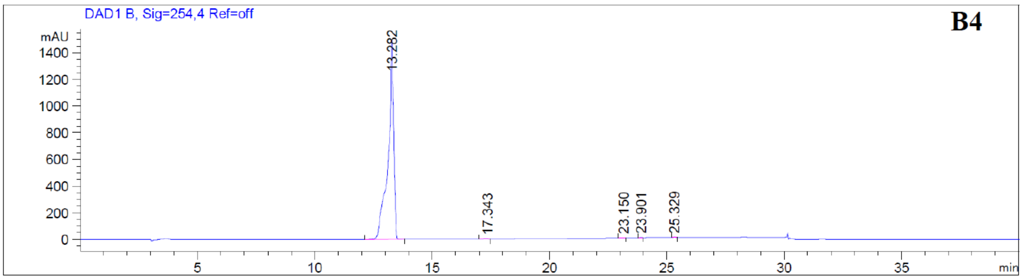

Signal 2: DAD1 B, Sig=254, 4 Ref=off

Peak #	Retention time [min]	Type	Peak Width [min]	Peak area [mAU*s]	Peak height [mAU]	Peak area %
1	13. 282	BB	0. 2224	2. 50013e4	1500. 56873	99. 7236
2	17. 343	BB	0. 1452	38. 32016	3. 46009	0. 1528
3	23. 150	BB	0. 0946	6. 64218	1. 09832	0. 0265
4	23. 901	BB	0. 0753	6. 38861	1. 30088	0. 0255
5	25. 329	BB	0. 0897	17. 94167	3. 09024	0. 0716

Total amount: 2. 50706e4 1509. 51826

Figure S124: HPLC spectrum of B4.

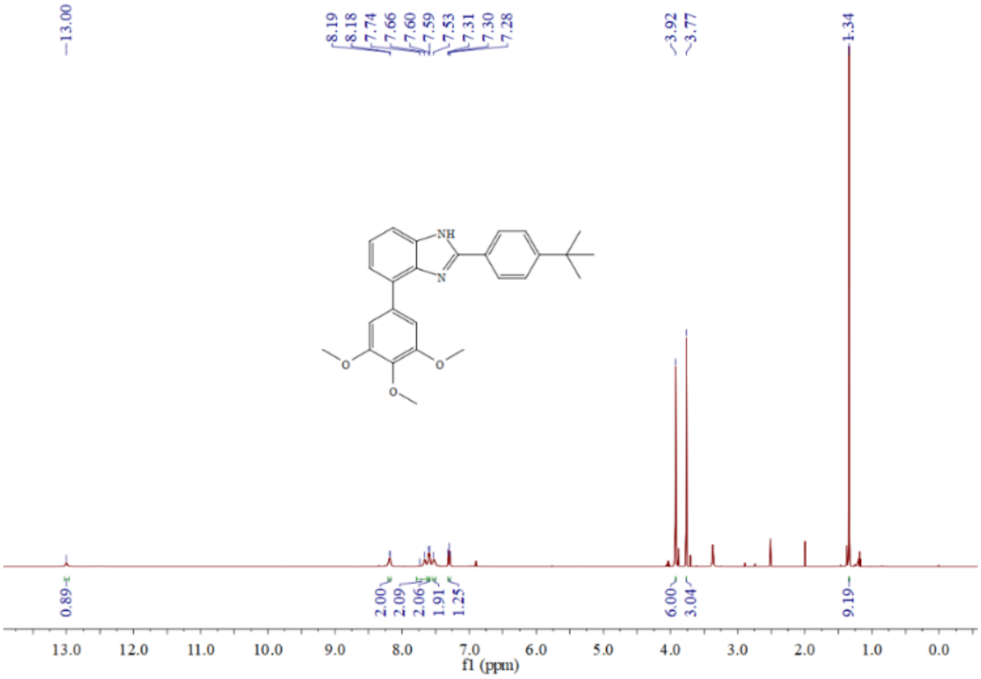

Figure S125: ^{1}H NMR spectrum of B5.

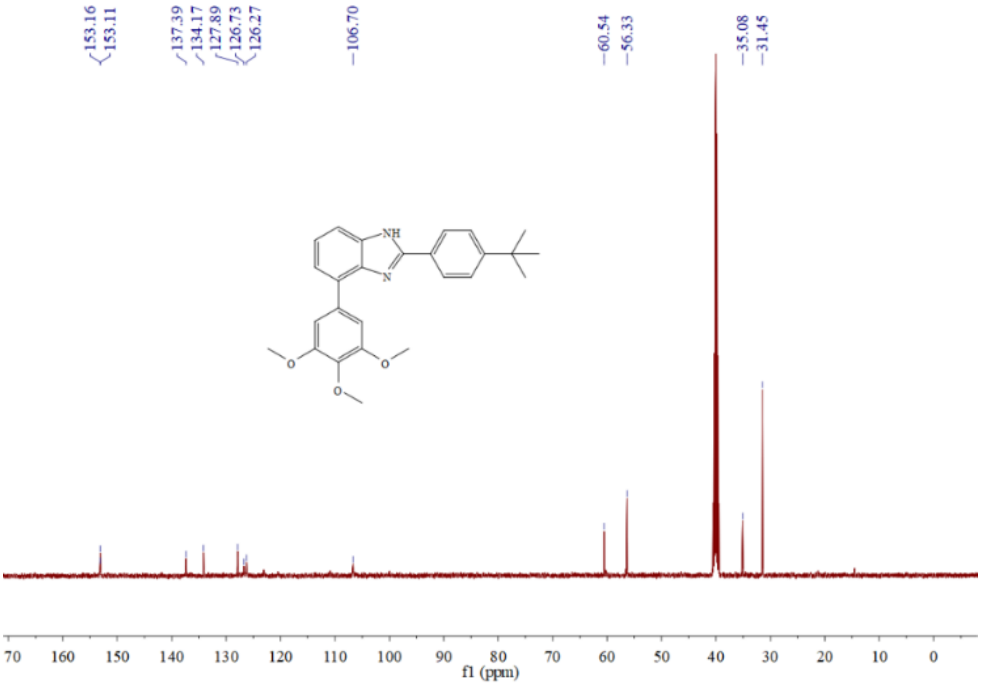

Figure S126: ^{13}C NMR spectrum of B5.

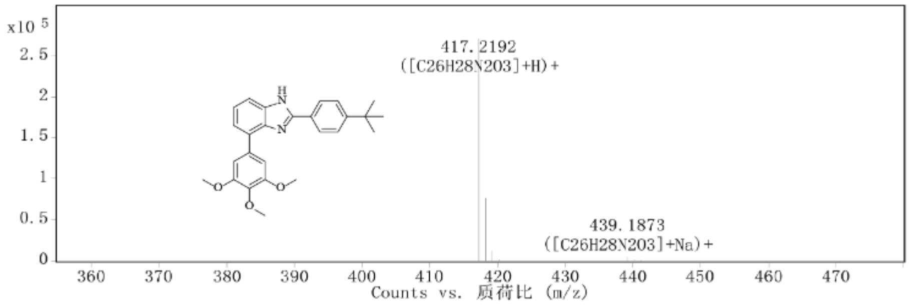

Figure S127: HR MS spectrum of B5.

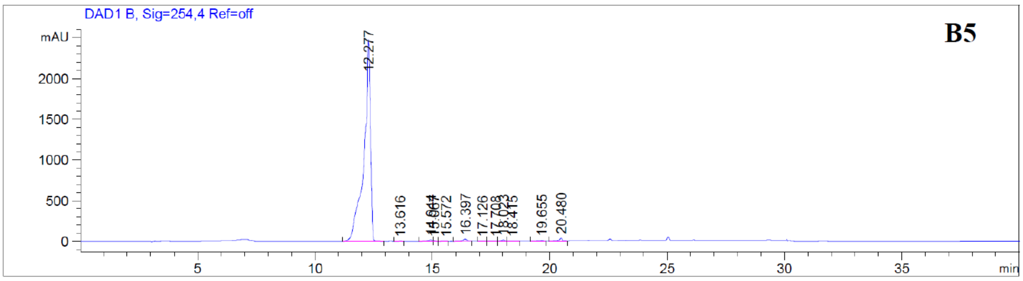

Signal 2: DAD1 B, Sig=254,4 Ref=off

Peak #	Retention time [min]	Type	Peak Width [min]	Peak area [mAU*s]	Peak height [mAU]	Peak area %
1	12.277	BB	0.2642	4.81339e4	2482.19653	97.4425
2	13.616	BB	0.1680	13.47446	1.03454	0.0273
3	14.944	BV	0.1571	235.89587	20.07136	0.4775
4	15.067	VB	0.0714	32.57030	6.86456	0.0659
5	15.572	BB	0.1213	29.85935	3.43818	0.0604
6	16.397	BB	0.1518	342.78955	30.34657	0.6939
7	17.126	BB	0.1057	10.95198	1.45749	0.0222
8	17.708	BV	0.1397	33.94115	3.36567	0.0687
9	18.023	VV	0.1310	151.54378	16.22088	0.3068
10	18.415	VB	0.1688	43.80080	3.38773	0.0887
11	19.655	BB	0.1401	85.67083	8.32068	0.1734
12	20.480	VV R	0.1152	282.81381	34.65544	0.5725
Total amount:				4.93972e4	2611.35964	

Figure S128: HPLC spectrum of B5.

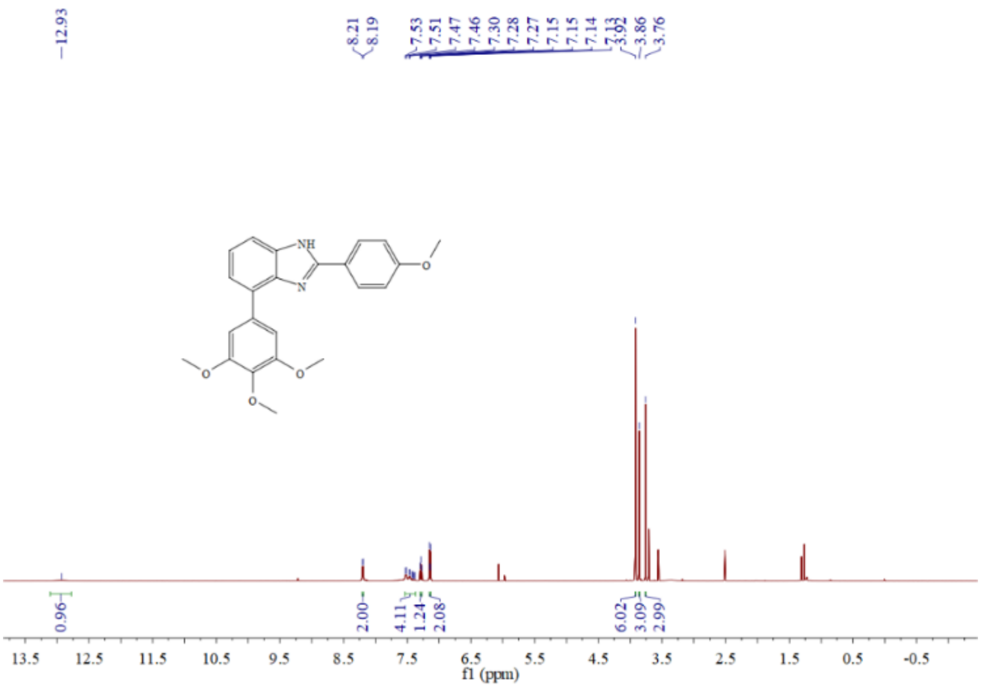

Figure S129: ^{1}H NMR spectrum of B6.

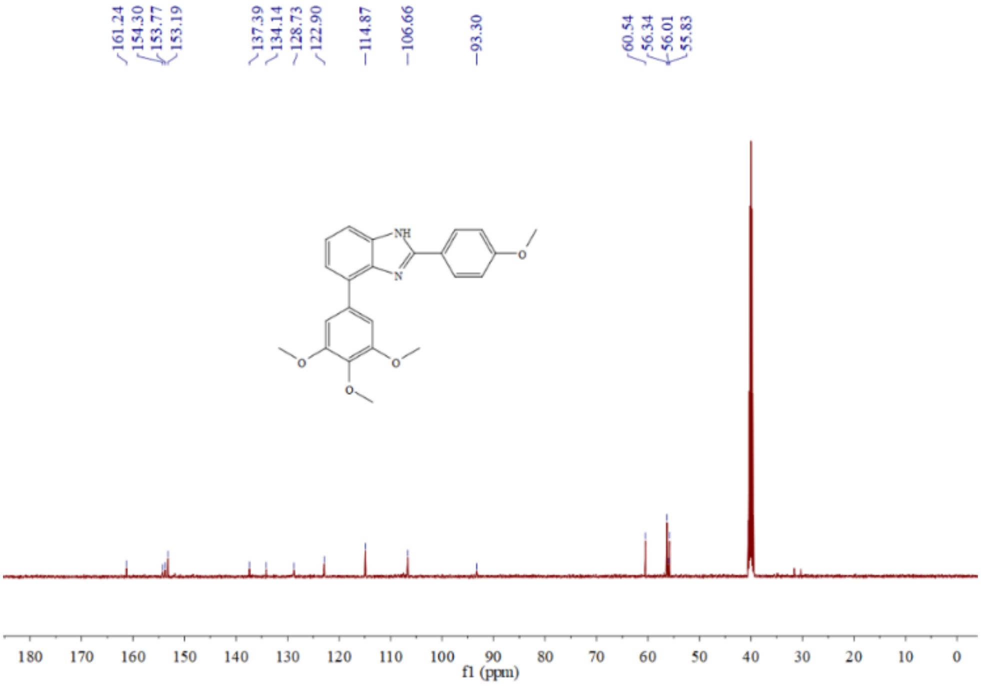

Figure S130: ^{13}C NMR spectrum of B6.

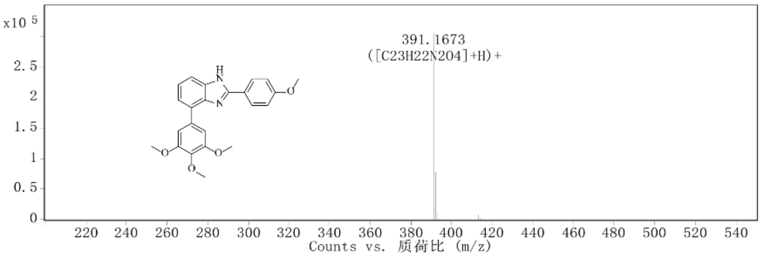

Figure S131: HR MS spectrum of B6.

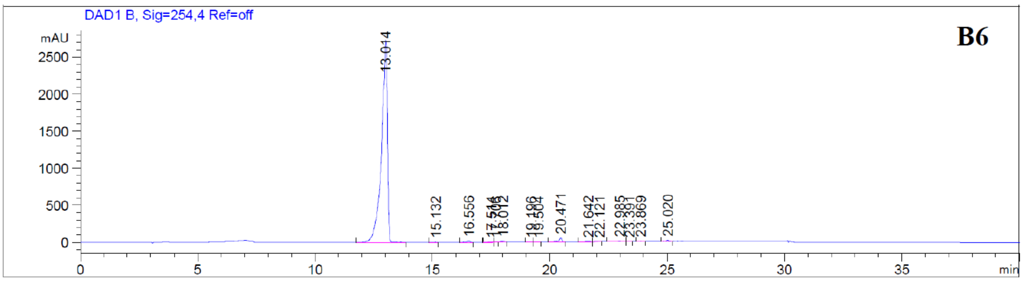

Signal 2: DAD1 B, Sig=254,4 Ref=off

Peak #	Retention time [min]	Type	Peak Width [min]	Peak area [mAU*s]	Peak height [mAU]	Peak area %
1	13.014	BV R	0.2395	4.79764e4	2723.56958	97.8605
2	15.132	BB	0.1260	18.39600	1.98643	0.0375
3	16.556	BB	0.1341	121.72767	12.67315	0.2483
4	17.514	BV E	0.1148	22.37799	2.69683	0.0456
5	17.706	VV E	0.1049	11.10897	1.56285	0.0227
6	18.012	VB R	0.1440	161.08162	15.66527	0.3286
7	19.196	BB	0.0994	26.23809	3.85307	0.0535
8	19.504	BB	0.1125	7.96223	1.04965	0.0162
9	20.471	BB	0.1179	440.67874	53.60333	0.8989
10	21.642	BB	0.1237	36.65733	4.20603	0.0748
11	22.121	BB	0.1064	22.16220	2.99220	0.0452
12	22.985	VV R	0.1677	67.85994	5.50903	0.1384
13	23.391	BB	0.1013	8.45332	1.21236	0.0172
14	23.869	BB	0.0943	11.84696	1.86128	0.0242
15	25.020	VB R	0.0935	92.33723	15.07558	0.1883
Total amount:				4.90253e4	2847.51666	

Figure S132: HPLC spectrum of B6.

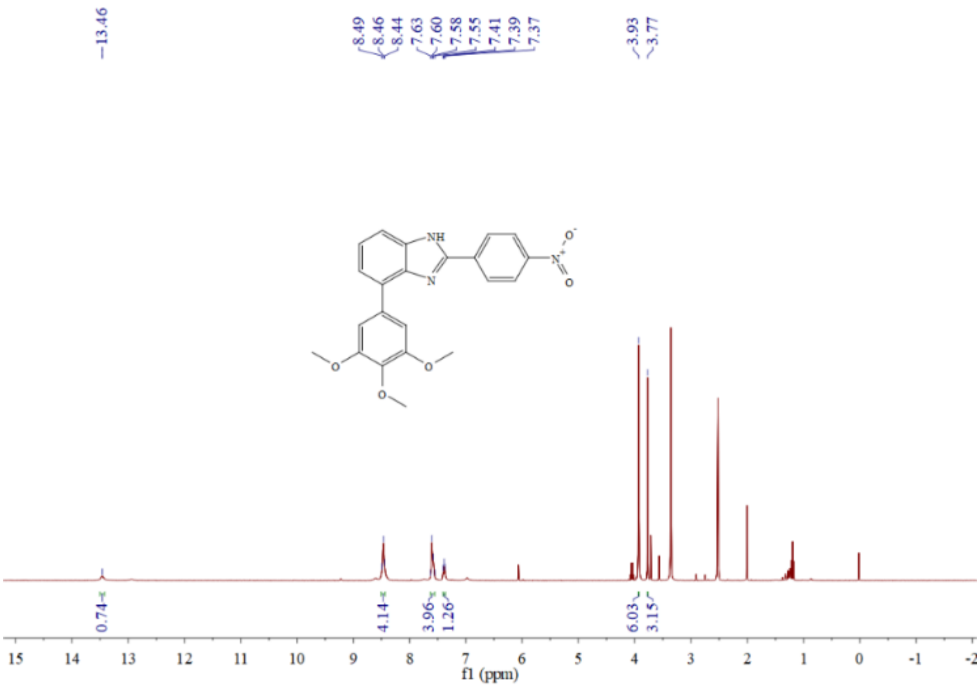

Figure S133: ^{1}H NMR spectrum of B7.

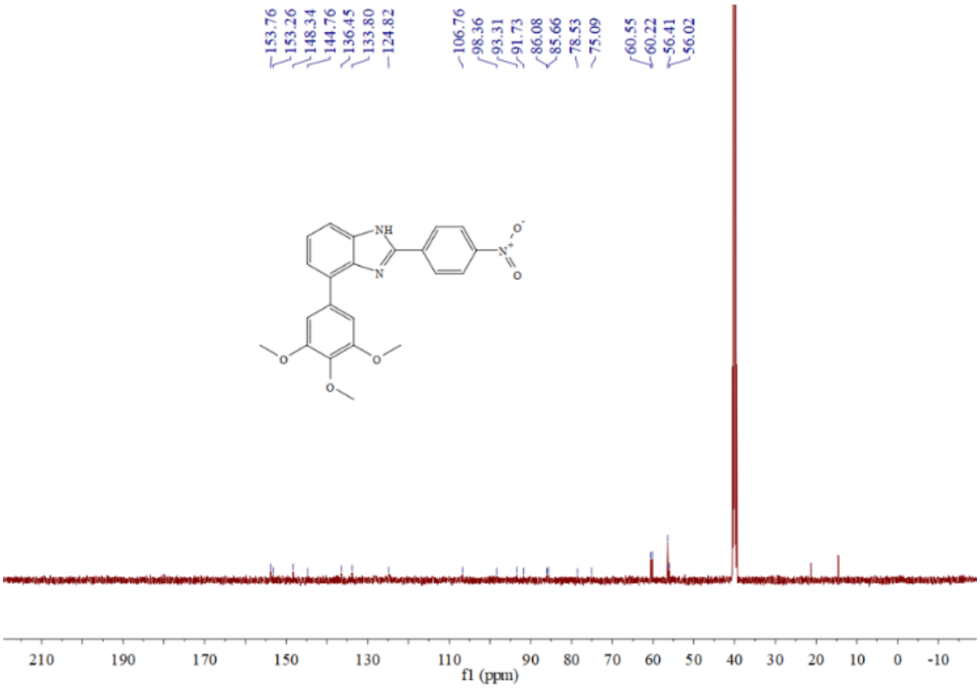

Figure S134: ^{13}C NMR spectrum of B7.

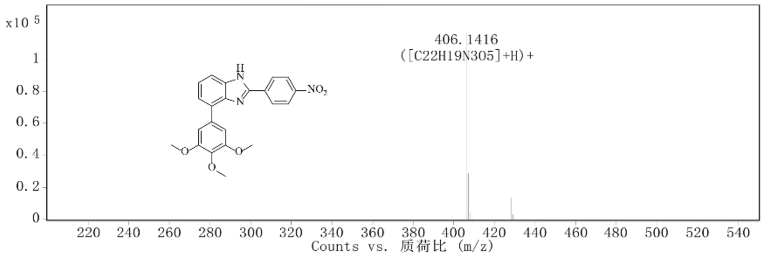

Figure S135: HR MS spectrum of B7.

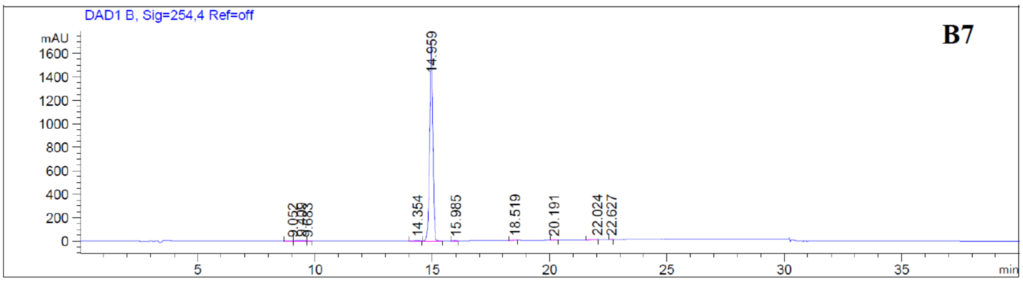

Signal 2: DAD1 B, Sig=254,4 Ref=off

Peak #	Retentio time [min]	Type	Peak Width [min]	Peak area [mAU*s]	Peak height [mAU]	Peak area %
1	9.052	BV	0.1606	18.51516	1.45318	0.1122
2	9.409	VV	0.3207	67.12093	2.48817	0.4067
3	9.683	VB	0.0772	7.95016	1.34004	0.0482
4	14.354	BB	0.1267	41.80086	4.48526	0.2533
5	14.959	BV R	0.1414	1.62996e4	1707.46912	98.7560
6	15.985	VB	0.1326	14.38113	1.51730	0.0871
7	18.519	BB	0.1340	12.96883	1.22014	0.0786
8	20.191	BV	0.1250	17.45817	1.71196	0.1058
9	22.024	BV	0.2191	19.18258	1.06009	0.1162
10	22.627	BV	0.0827	5.94094	1.03918	0.0360
Total amount:				1.65050e4	1723.78443	

Figure S136: HPLC spectrum of B7.

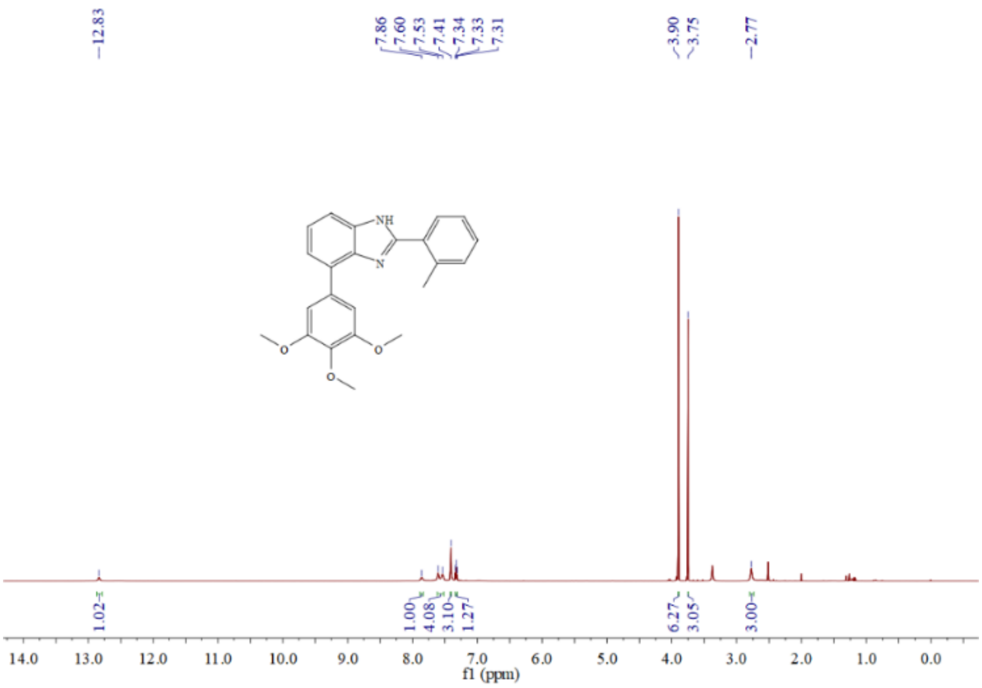

Figure S137: ^{1}H NMR spectrum of B8.

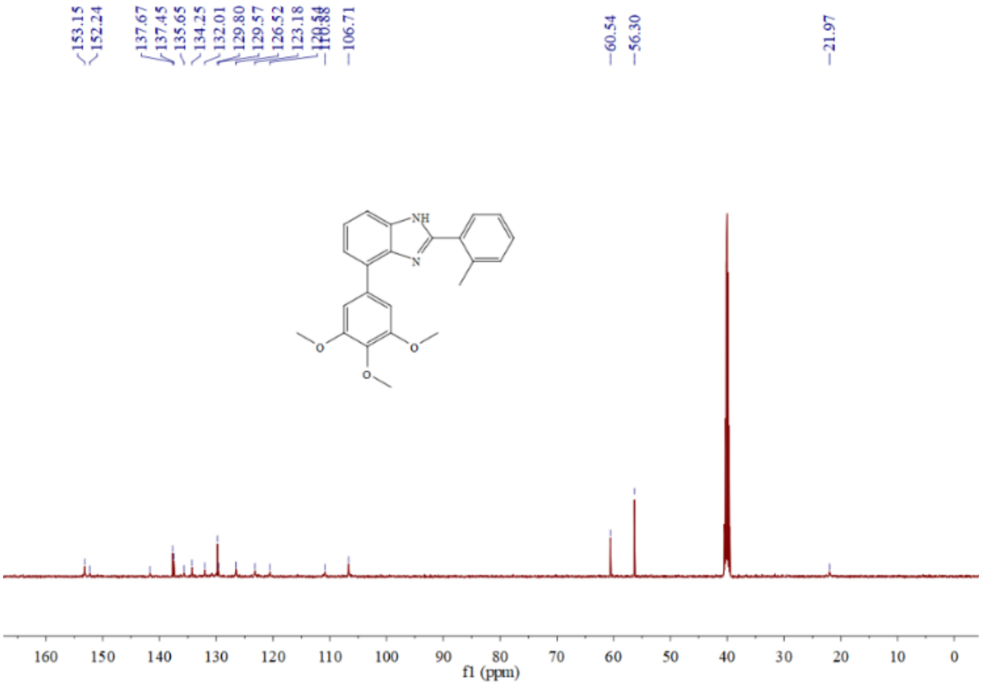

Figure S138: ^{13}C NMR spectrum of B8.

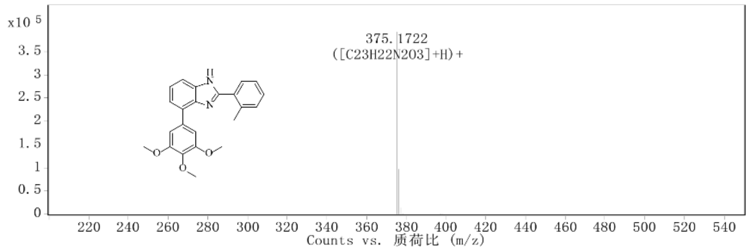

Figure S139: HR MS spectrum of B8.

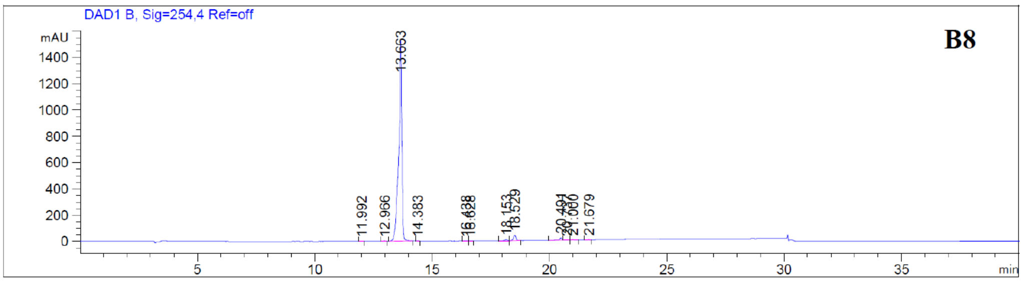

Signal 2: DAD1 B, Sig=254,4 Ref=off

Peak #	Retention time [min]	Type	Peak Width [min]	Peak area [mAU*s]	Peak height [mAU]	Peak area %
1	11.992	BB	0.1047	8.24680	1.16279	0.0557
2	12.966	BB	0.0924	8.71450	1.33119	0.0589
3	13.663	BB	0.1310	1.42594e4	1525.59216	96.3902
4	14.383	BB	0.0834	8.62522	1.58702	0.0583
5	16.438	BV	0.1319	16.35225	1.73613	0.1105
6	16.628	VB	0.1028	9.03869	1.27306	0.0611
7	18.153	BV	0.1061	56.88834	7.88958	0.3846
8	18.529	VB	0.1020	278.98010	40.69282	1.8858
9	20.491	BV R	0.0972	113.13433	17.09319	0.7648
10	20.737	VV E	0.1154	9.20289	1.20058	0.0622
11	21.000	VB	0.1022	18.46085	2.68392	0.1248
12	21.679	BB	0.0905	6.37136	1.05459	0.0431
Total amount:				1.47935e4	1603.29705	

Figure S140: HPLC spectrum of B8.

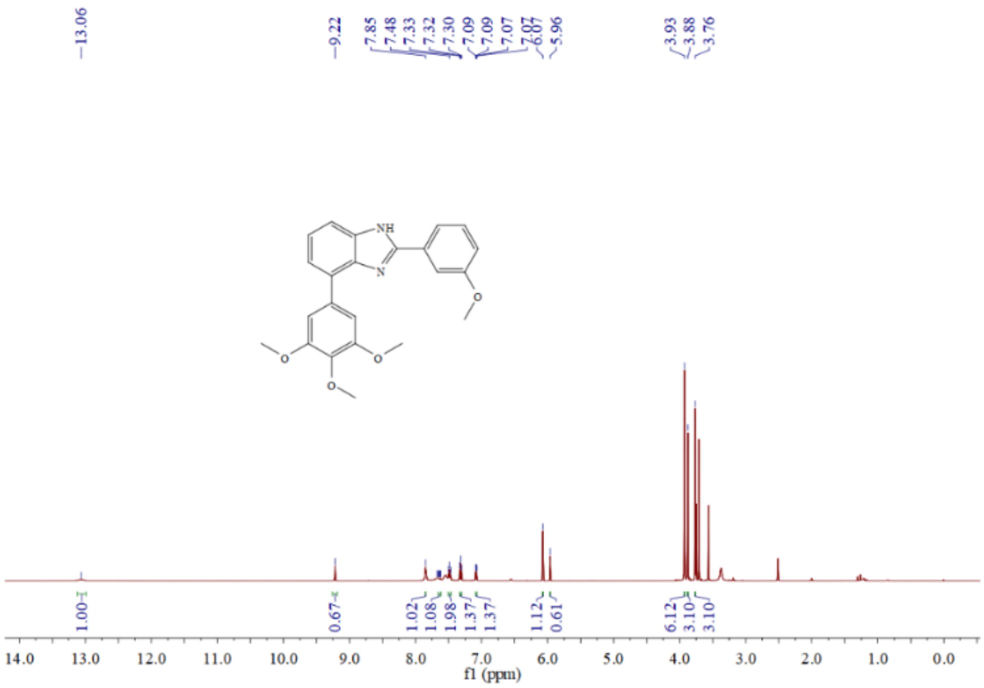

Figure S141: 1H NMR spectrum of B9.

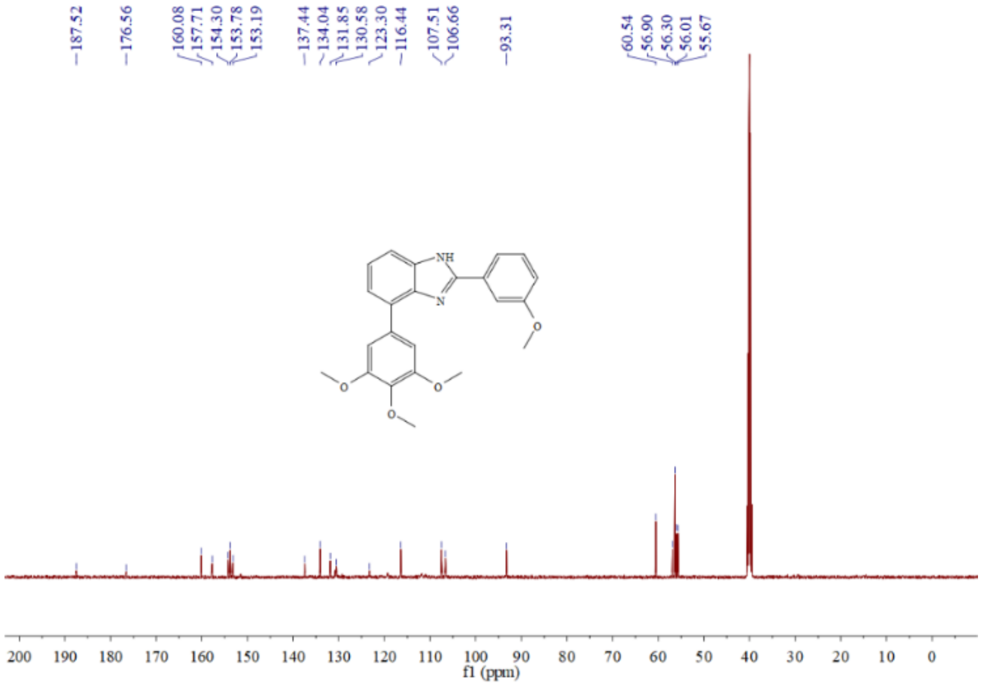

Figure S142: 13C NMR spectrum of B9.

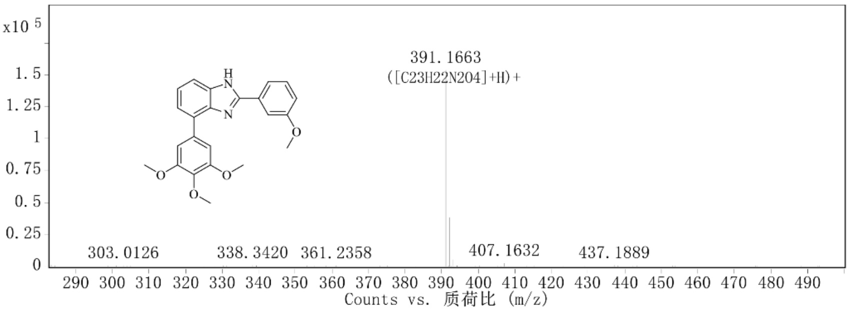

Figure S143: HR MS spectrum of B9.

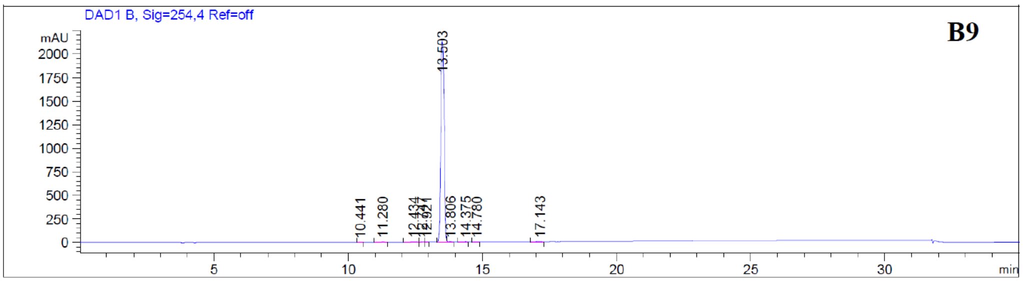

Signal 2: DAD1 B, Sig=254,4 Ref=off

Peak #	Retention time [min]	Type	Peak Width [min]	Peak area [mAU*s]	Peak height [mAU]	Peak area %
1	10.441	BB	0.0936	13.89629	2.32925	0.0759
2	11.280	BB	0.0978	40.37360	5.90170	0.2205
3	12.434	BV	0.2146	25.04284	1.54937	0.1368
4	12.747	VV	0.0816	7.34850	1.30749	0.0401
5	12.921	VB	0.0772	6.75994	1.33284	0.0369
6	13.503	BV R	0.1388	1.81222e4	2139.45093	98.9933
7	13.806	VB E	0.1105	17.34498	2.28770	0.0947
8	14.375	BB	0.1468	28.10103	2.50591	0.1535
9	14.780	BB	0.1335	13.04302	1.52938	0.0712
10	17.143	BB	0.1656	32.38505	2.63089	0.1769
Total amount:				1.83065e4	2160.82547	

Figure S144: HPLC spectrum of B9.

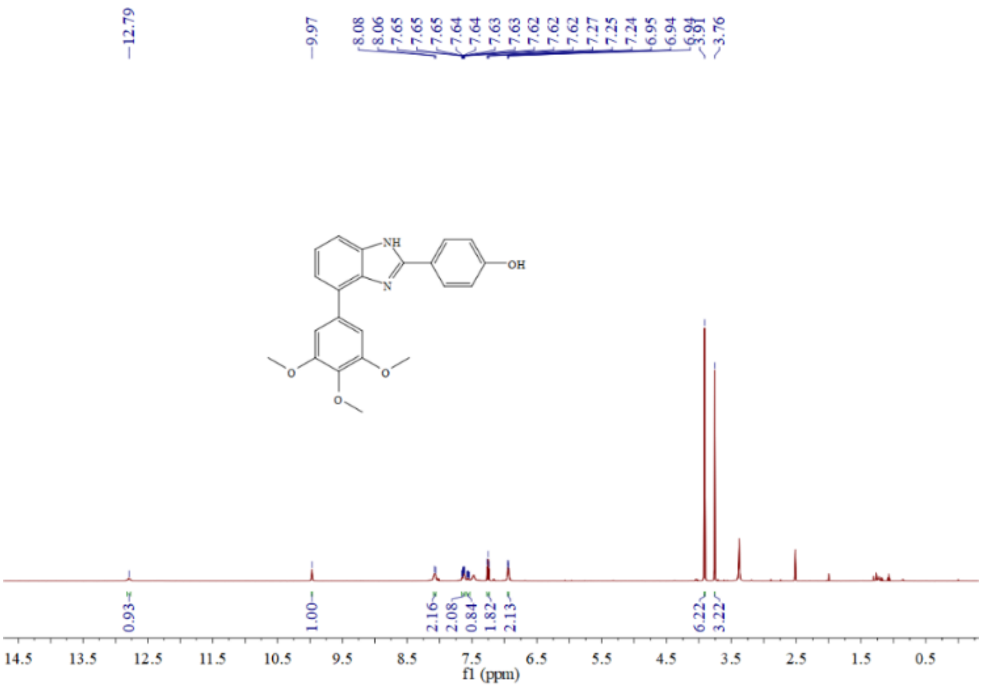

Figure S145: ^{1}H NMR spectrum of B10.

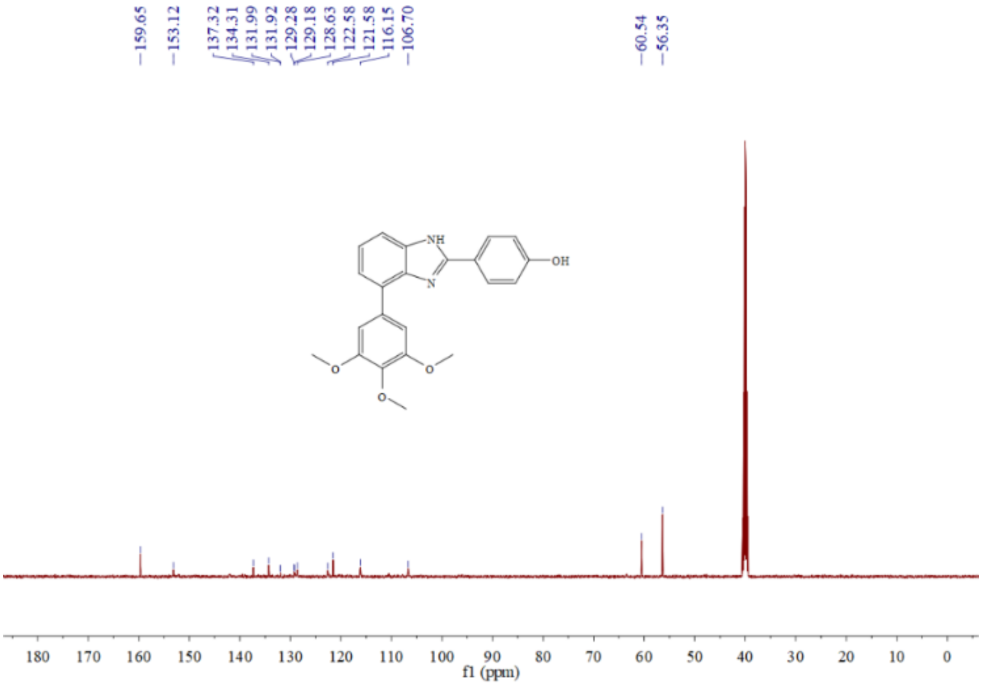

Figure S146: ^{13}C NMR spectrum of B10.

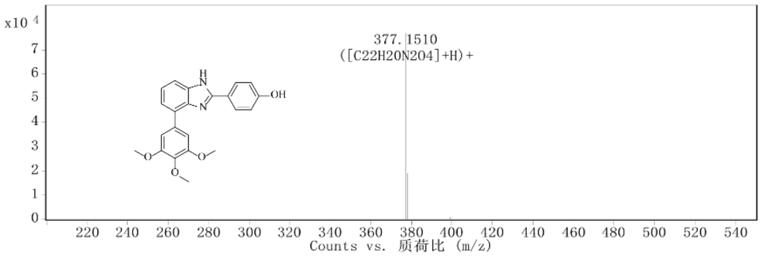

Figure S147: HR MS spectrum of B10.

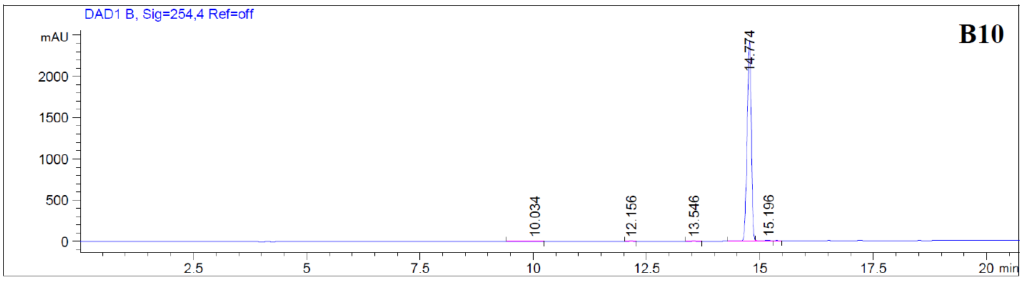

Signal 2: DAD1 B, Sig=254,4 Ref=off

Peak #	Retention time [min]	Type	Peak Width [min]	Peak area [mAU*s]	Peak height [mAU]	Peak area %
1	10.034	BB	0.2525	30.25274	1.53133	0.1932
2	12.156	BB	0.0870	23.09680	4.14447	0.1475
3	13.546	BB	0.1171	8.88728	1.04665	0.0567
4	14.774	BV R	0.0961	1.55343e4	2443.01953	99.1830
5	15.196	VV E	0.1545	65.72705	6.58203	0.4197

Total amount: 1.56623e4 2456.32401

Figure S148: HPLC spectrum of B10.
